# Temporal constraints on enhancer usage shape the regulation of limb gene transcription

**DOI:** 10.1038/s41467-025-66055-6

**Published:** 2026-01-12

**Authors:** Raquel Rouco, Antonella Rauseo, Fabrice Darbellay, Guillaume Sapin, Olimpia Bompadre, Lucille Lopez-Delisle, Guillaume Andrey

**Affiliations:** 1 Department of Genetic Medicine and Development, Faculty of Medicine, University of Geneva, Geneva, Switzerland; 2 Institute of Genetics and Genomics in Geneva (iGE3), University of Geneva, Geneva, Switzerland

**Keywords:** Gene regulation, Pattern formation, Embryogenesis

## Abstract

Enhancer repertoires orchestrate gene expression during embryonic development, shaping organ structure and function. Individual enhancers can act in overlapping or distinct spatial domains, but their temporal specificity and coordinated action over time remain poorly understood. Here, we identify temporally restricted enhancer repertoires at multiple loci involved in mouse limb development. To capture their dynamic roles, we introduce the regulatory trajectory framework comprising initiation, maintenance, and decommissioning of gene expression. Using a transgenic recorder at the *Shox2* locus, we demonstrate that early enhancers initiate transcription, while late enhancers maintain it. Additionally, we found that changes in 3D topology associate with enhancer activities and that loss of enhancer-promoter contacts occurs during decommissioning. Finally, we show that *Shox2* regulatory decommissioning can be driven by *Hoxd13*, a known antagonist of *Shox2* expression. Overall, our work uncovers how temporally restricted enhancers generate complex expression patterns and sheds light on the dynamics of enhancer-promoter interactions.

## Abstract (web summary)

This study reveals how temporally restricted enhancers control gene expression over time, highlighting their role in transcription dynamics and enhancer–promoter interactions during mouse limb development.

## Introduction

Organ and tissue patterning depend on the spatiotemporally-defined onset and silencing of gene transcription, which is regulated by transcriptional enhancers^1,2^. Enhancers serve as platforms for transcription factors (TFs) that convey cellular environmental information to drive specific transcriptional outputs^3^. Within the same chromatin domains, known as Topologically Associating Domains (TADs), genes can interact with multiple enhancers, referred to as enhancer repertoires, that collectively shape their expression patterns^2,4–8^.

While multiple studies have explored how individual enhancers act with spatially distinct or overlapping activities^1,2,9,10^, some have reported stage-specific activities^11–14^. However, the prevalence of stage-specific enhancers during organ development and how their sequential activities are integrated to form gene expression patterns over time remain largely unexplored. In the *Drosophila* bithorax locus (BX-C), the sequential activation of enhancers within restricted genomic regions drives gene transcription initiation and maintenance over time^15^. Initiator elements act first to induce gene transcription, displaying activity in undifferentiated parasegments, where they open *cis*-regulatory domains containing maintenance elements. In turn, these elements, which bear cell-type-specific activities, sustain transcription in differentiating daughter cells^15–17^. In mammals, where enhancers are spread over large regulatory landscapes and not in compact domains, it is so far unknown if a similar class of enhancers enables to adapt to the changing cellular and signaling environments of a growing organ to build gene expression patterns over time. Finally, the initial steps that associate with the shutdown of multi-enhancer landscapes in vivo have received relatively little attention, despite being potentially a crucial mechanism for establishing gene expression patterns.

Many complex regulatory landscapes have been dissected using the limb model system^1,18–20^. Fore- and hindlimbs are budding from the lateral plate mesoderm at E9.5 and E10.0, respectively^21^. Initially, the limb is predominantly composed of undifferentiated mesenchyme, capable of forming all proximodistal segments. However, as development progresses, the ability to form more proximal segments gradually diminishes as cells set their positional identity^22^. Mechanistically, it has been proposed that as limb progenitor cells begin expressing patterning genes, they can either commit to the associated specific limb segment or maintain their progenitor status  eventually activating more distal factors and repressing the more proximal ones. Thus, once a proximodistal patterning gene is activated in limb progenitors, its expression could either be maintained during lineage commitment or inhibited to permit differentiation into a different, more distal limb segment^23–25^. Therefore, the limb is an adequate model system to define how transcriptional regulation can be maintained or repressed over time.

In this study, we aimed to characterize the regulatory mechanisms controlling the temporal dynamics of gene expression during limb development. Specifically, we sought to determine whether distinct regulatory elements control regulatory initiation and subsequent regulatory maintenance over time, and to shed light on the processes leading to regulatory decommissioning and gene repression. To allow for the functional dissection of these mechanisms, we are using the *Shox2* gene locus that bears an essential role in proximal limb development. *Shox2* expression starts in the limb around E9.5/E10.0 and continues to be expressed in proximal connective tissues and cartilage, while it is excluded from the handplate, playing a crucial role in the growth of the humerus and the femur, two proximal limb bones^26–34^.

The regulatory landscape of *Shox2* is embedded within a 1.1 Mb TAD that splits into a centromeric side featuring a 500 kb gene desert and a telomeric side, largely composed of the introns of the *Rsrc1* gene. Numerous limb enhancers have been identified on both the telomeric and centromeric sides, collectively contributing to *Shox2* limb expression^8,9,35,36^. Although these enhancer regions have been shown to drive expression in the limb and other *Shox2*-expressing tissues, the specific time windows of their activity have not been determined.

Given that *Shox2* expression is initiated in the early, undifferentiated limb bud and gradually becomes restricted to more proximal limb mesodermal derivatives, its regulation must involve mechanisms of maintenance and repression via precise enhancer activation and decommissioning, respectively. To accurately track descendants of *Shox2*-expressing cells within the complex and diversely populated developing limb, we have devised a conceptual and experimental framework to follow *Shox2* locus activity. This approach enables the isolation of cells at distinct phases of *Shox2* transcriptional regulation, offering insights into the dynamic control of gene expression during limb development.

## Results

### Characterization of temporal enhancer repertoire changes at limb development-associated *loci*

To examine whether *Shox2*, and more generally limb developmental genes, could rely on distinct enhancer repertoire over time during limb development, we first reanalyzed transcriptomic and epigenomic data from the E10.5 and E13.5 limb^37^. Specifically, we selected 90 genes relevant for limb development, including *Shox2*, which are expressed at both stages. We then mapped putative enhancers of these genes using H3K27ac ChIP-seq enrichment within their contact domains, as defined by promoter Capture-C in the limb^37,38^ (Supplementary Data 1).

**Fig. 1:**
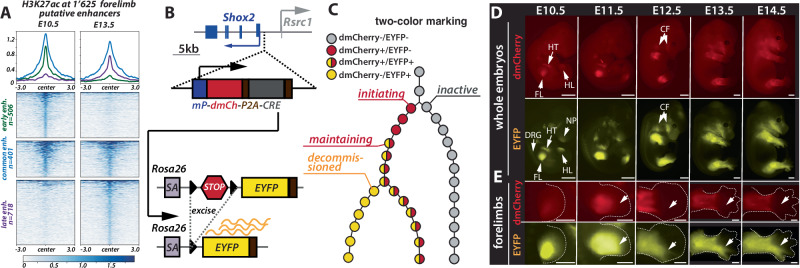
A transgenic reporter system to characterize the *Shox2* regulatory trajectory. **A** Distribution of H3K27ac ChIP-seq signal^37^ across the 1625 putative enhancers (Supplementary Data 1) identified within 90 loci of limb-expressed genes at E10.5 and E13.5. The tornado plots are split into early-, common-, and late-acting enhancers and are centered on the enhancer peaks, with a 3 kb region on either side. **B** A double fluorophore approach to monitor *Shox2* locus activity over time; mP minimal β-globin promoter, dmCh mCherry gene with a destabilized PEST sequence, P2A ribosomal skipping sequence, CRE CRE recombinase gene, SA splice acceptor, STOP floxed 3x SV40pA STOP signal, EYFP EYFP gene. **C** Schematic of the double fluorophore approach that enables tracking of *Shox2* regulatory trajectory: different color combinations correlate with different phases of the trajectory. **D** Imaging of dmCherry and EYFP fluorescence in *Shox2*^*trac*^ (*Shox2*^*dmCherry/+*^; *Rosa26*^*loxEYFP/+*^) embryos (scale: 1 mm); HT heart, FL forelimbs, HL hindlimbs, NP nasal process, DRG dorsal root ganglia, CF craniofacial structures. **E** Imaging of dmCherry and EYFP fluorescence in developing *Shox2*^*trac*^ forelimbs (scale bars: 500 μm). Note that cells in digits 4 and 5 (white arrows) are positive for EYFP but negative for dmCherry. In (**D**, **E**), the same embryos were used, and in total, 7 E10.5, 10 E11.5, 8 E12.5, 11 E13.5, and 10 E14.5 embryos were imaged and showed similar patterns.

Across the 90 investigated contact domains, we observed a total of 1625 putative enhancers, defined by H3K27ac peaks outside of promoter regions. Of these, 401 were active at both E10.5 and E13.5 (25%, termed common-acting (or common) enhancers), 506 were specifically active at E10.5 (31%, termed early-acting (or early) enhancers), and 718 at E13.5 (44%, termed late-acting (or late) enhancers) (Fig. 1A). A large majority of these contact domains (76 of them, 84%), including *Shox2*, contained all three types of enhancers, a smaller fraction (13%) presents at least two types while only two loci display putative enhancers belonging to a unique category (Supplementary Fig. 1A). This observation, which is exemplified in Supplementary Fig. 1B by *Shox2* and two well-known limb-associated loci, *Sox11* and *Sox9*, suggests that within the limb context, the usage of putative enhancer repertoires generally shift over time. Therefore, the *Shox2* locus, which displays 13 early, 2 common, and 4 late putative enhancers (Supplementary Fig. 1B), is a representative example of limb developmental genes to study how enhancer repertoires control gene transcription over time.

### A transgenic reporter system to characterize the *Shox2* regulatory trajectory

To carefully analyze the regulatory dynamics of gene expression across extended developmental periods, we conceptually divide it into different states that collectively define a regulatory trajectory. Such a trajectory begins from a poised state, from which a locus may transit towards an active state via regulatory *initiation*, or towards a repressed state via *repression* (Supplementary Fig. 1C)^39–41^. After regulatory *initiation*, it is eventually followed by a *maintenance* phase where gene transcription is sustained over time. Finally, a *decommissioning* phase progressively leads to gene repression (Supplementary Fig. 1C).

To monitor the *Shox2* regulatory trajectory and sort cells undergoing these distinct phases, we have developed a dual-color transgenic reporter system. First, we inserted in mouse embryonic stem cells (mESCs), 1 kb upstream of the *Shox2* transcriptional start site, a regulatory sensor cassette constituted by a minimal *β-globin* promoter, a mCherry reporter open reading frame (ORF) followed by a destabilizing PEST sequence, a P2A ribosomal skipping sequence and the CRE recombinase ORF (*Shox2*^*dmCherry*/+^) (Fig. 1B)^42–46^. These *Shox2*^*dmCherry/+*^ cells were then retargeted to integrate, at the *Rosa26* locus, a cassette with a splice acceptor followed by a floxed 3x SV40pA STOP signal and the EYFP ORF (*Shox2*^*dmCherry/+*^; *Rosa26*^*loxEYFP/+*^ or *Shox2*^*trac*^) (Fig. 1B)^47^. With this system, *Shox2*-expressing cells will induce dmCherry-P2A-CRE transcription and also continuously express *EYFP*, even after the silencing of *Shox2* and dmCherry-P2A-CRE transcription. Here, cells undergoing regulatory *initiation* should correspond to early limb *Shox2* expression and dmCherry-positive cells (Fig. 1C). Cells with dmCherry and EYFP signals will indicate regulatory *maintenance*, and cells with EYFP only will have undergone regulatory *decommissioning* after an earlier active transcriptional phase, while cells that remained *inactive* will have no fluorescent labeling (Fig. 1C).

Embryos were then obtained from these *Shox2*^*trac*^ mESCs by tetraploid complementation^48^. First, to assess any potential regulatory impact of the dmCherry-P2A-CRE cassette, we performed RNA-seq on *Shox2*^*trac*^ and wild-type E12.5 forelimbs and observed no changes in *Shox2* transcription (Supplementary Data 2). At E10.5, we detected dmCherry and EYFP signals in the fore-(FL) and hindlimbs (HL) as well as in the heart (HT) (Fig. 1D). We also detected EYFP signal in the nasal process (NP) and dorsal root ganglia (DRG). While the dmCherry signal in hindlimbs was weak at E10.5 compared to forelimbs, it markedly increased by E11.5, reflecting the expected developmental delay of hindlimbs compared to forelimbs^21^. Subsequently, at E12.5, additional craniofacial (CF) structures exhibited fluorescence from both dmCherry and EYFP (Fig. 1D). These expression patterns closely match the known expression profile of *Shox2* (Supplementary Fig. 1D, E)^32,49–51^. Intriguingly, within the limbs, we observed that digits 4 and 5, located in the posterior autopod, were exclusively marked by EYFP and not by dmCherry (Fig. 1E, Supplementary Fig. 1E). This indicates that part of the distal posterior limb originates from progenitor cells that had expressed *Shox2* at earlier stages, but whose regulatory landscape was subsequently decommissioned.

### Single-cell insights into *Shox2* transcriptional dynamics trace early *decommissioned* cells in the distal limb

**Fig. 2:**
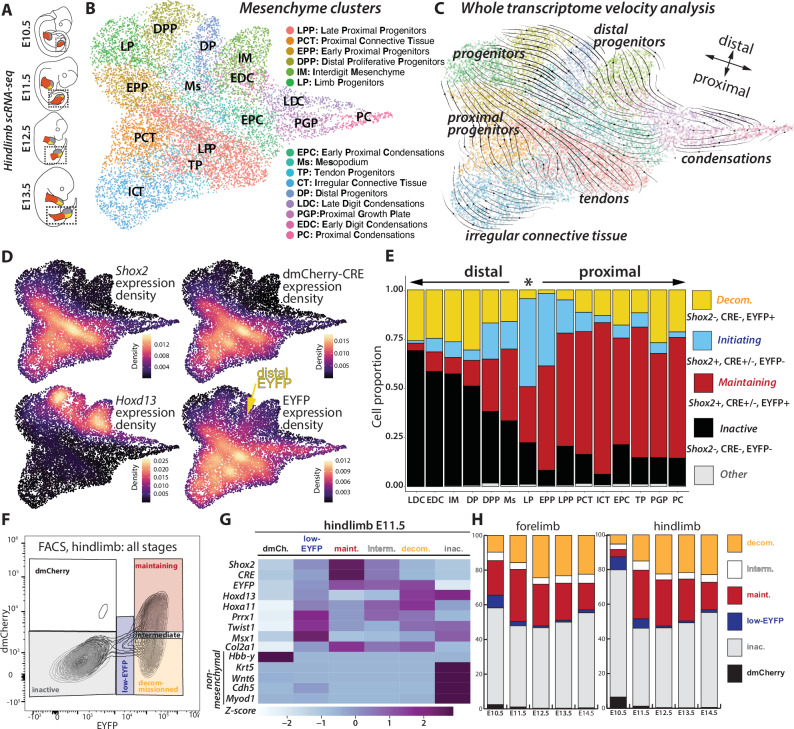
Characterizing the dual-color reporter approach across distinct phases of *Shox2* regulatory trajectory. **A** Illustration of single-cell preparation from micro-dissected *Shox2*^*trac*^ hindlimbs at E10.5, E11.5, E12.5, and E13.5. **B** UMAP visualization of re-clustered mesenchymal cells from all merged datasets. **C** RNA-velocity analysis across mesenchymal cell clusters. **D** Analysis of gene expression density for *Shox2* (proximal marker), dmCherry-P2A-CRE, *Hoxd13 (distal marker)*, and EYFP in mesenchyme cells. Note the EYFP expression in distal clusters, in contrast to *Shox2* and dmCherry-CRE. **E** Distribution of cell proportions categorized by the different *Shox2* regulatory phases: *initiating* (*Shox2*+, dmCherry-P2A-CRE+/−, EYFP−), *maintaining* (*Shox2*+, dmCherry-P2A-CRE+/−, EYFP+), *decommissioned* (*Shox2*−, dmCherry-P2A-CRE−, EYFP+), *inactive* (*Shox2*−, dmCherry-P2A-CRE−, EYFP−) or other cells (that where not included in any of the previously mentioned class) across mesenchymal clusters. Clusters are ordered according to their distal and proximal identity and to developmental time, with the limb progenitor cluster highlighted by a star. Source data are provided in the Source data file. **F** FACS plot of *Shox2*^*trac*^ hindlimbs dissociated cells illustrating the distribution of cells based on dmCherry and EYFP fluorescence in all the developmental stages investigated. **G** Heatmap profiles of representative marker genes in different sorted populations from E11.5 hindlimbs. dmCh. dmCherry, maint. maintaining, interm. intermediate, inac. inactive. *Z*-score scale derived from normalized FPKMs provides a normalized measure by rows of gene expression levels enabling comparison across samples. Source data are provided in the Source data file and in Supplementary Data File 4. **H** Proportions of each sorted population at E10.5, E11.5, E12.5, E13.5, and E14.5 in both forelimbs and hindlimbs, showcasing the dynamic changes in population sizes over developmental time. *Inactive* (EYFP-, dmCherry-, in gray), *maintaining* (EYFP+, dmCherry+, in red), *decommissioned* (EYFP+, dmCherry−, in yellow), blood (EYFP−, dmCherry+, in black), low-EYFP (low EYFP+, low dmCherry+, in blue), intermediate (high EYFP+, intermediate dmCherry+, in white). Source data are provided in the Source data file.

To gain a detailed understanding of transcriptional dynamics during limb development, and of *Shox2* regulatory phases over time, we produced single-cell RNA-seq transcriptomes from *Shox2*^*trac*^ hindlimbs at four different embryonic stages: E10.5, E11.5, E12.5, and E13.5 (Fig. 2A). We then specifically focused our analyses on mesenchymal cells (*Prrx1*+, *Prrx2*+, *Twist1*+), where *Shox2* is expressed (Supplementary Fig. 2A, B). Merging the four timepoints investigated together, we identified 15 distinct clusters (Fig. 2B, Supplementary Data 3), from limb progenitors (LP, *Hoxd9/10/11*+, *Tfap2c*+, *Msx1*+, *Sall3/4*+) to more differentiated clusters at later stages (Supplementary Fig. 2C–E). To obtain insights into differentiation trajectories, we then ran a velocity analysis^52,53^ that predicted the E10.5 limb progenitor cluster to differentiate into both distal and proximal clusters (Fig. 2B, C). In proximal clusters, which are marked by *Shox2* expression (Supplementary Fig. 3A, B), progenitor pools (EPP and LPP) were predicted to differentiate into proximal condensations (PGP, EPC, and LPC) and connective tissues (PCT, ICT, and TP) (Fig. 2B, C). In distal clusters marked by *Hoxd13* expression (Supplementary Fig. 3B), progenitor pools (EDP and LDP) were predicted to differentiate into interdigit mesenchyme (IM) and distal condensations (EDC and LDC). The Mesopodium (Ms), the presumptive wrist domain, is predicted to derive directly from limb progenitors, expressing both proximal (*Shox2*) and distal markers (*Hoxd13*) at low levels (Fig. 2B, C, Supplementary Fig. 3B).

We then mapped within this clustering *dmCherry-P2A-CRE* and *EYFP* expressing cells from E10.5 onwards (Fig. 2D, Supplementary Fig. 2E and 3A). As expected from imaging data (Fig. 1E, Supplementary Fig. 1D), we observed a high correlation between *Shox2* and *dmCherry-P2A-CRE* transcription (correlation coefficient = 0.808, *p* = 0.00024, where the *p* value is the probability for the correlation coefficient to be negative^54^), indicating that the dmCherry-P2A-CRE cassette recapitulates *Shox2* expression. Interestingly, *EYFP* expression was observed in both proximal and distal limb clusters, though less frequently in the latter (Fig. 2D, Supplementary Fig. 3A), matching with our previous imaging observations (Fig. 1E, Supplementary Fig. 1E).

Leveraging the transcript levels of *Shox2*, *EYFP*, and *dmCherry-P2A-CRE* we then categorize cells into distinct phases of the *Shox2* regulatory trajectory. Given the low transcription levels of *dmCherry-P2A-CRE* in comparison to *EYFP* and *Shox2* (for technical reasons, see “Methods”), we primarily depended on the latter two genes’ expression to annotate the locus activity. We first observed that *Shox2-initiating* (*Shox2*+, *dmCherry-P2A-CRE*+*/−, EYFP-*) cells were primarily found in progenitor clusters (LP and EPP) (Fig. 2E, Supplementary Fig. 3C). This finding is also supported by a higher ratio of immature *Shox2* transcripts as measured by velocity analysis^52^ (Supplementary Fig. 3D). Cells in the *maintaining* (*Shox2*+, *dmCherry-P2A-CRE*+*/−, EYFP*+) phase were located in proximal limb clusters (Fig. 2E). *Decommissioned* (*Shox2−*, *dmCherry-P2A-CRE−, EYFP*+) cells were in both proximal and distal clusters supporting the whole transcriptome velocity analysis (Fig. 2C and E) and the existence of distal limb cells originating from *Shox2*-expressing limb progenitors. Finally, *inactive* (*Shox2−*, *dmCherry-P2A-CRE−, EYFP−*) cells were predominantly observed in distal clusters (Fig. 2E, Supplementary Fig. 3C). Over time, the proportion of *initiating* cells decreased concomitantly with an increase of *maintaining* and *decommissioned* cells (Supplementary Fig. 3E). In summary, while proximal clusters maintain *Shox2* expression initiated in limb progenitors, distal clusters increasingly silence the locus transcription.

### The dual-color reporter approach allows sorting of *inactive*, *maintaining*, and *decommissioned* cells over time

To characterize the dynamics of the *Shox2* regulatory landscape, we utilized fluorescence-activated cell sorting (FACS) on *Shox2*^*trac*^ fore- and hindlimb cells from E10.5 to E14.5 (Fig. 2F, Supplementary Fig. 4A). Before conducting chromatin analyses, we evaluated through bulk RNA-seq whether sorted cells matched the *Shox2* regulatory phases previously identified in the single-cell approach (Supplementary Data 3 and 4). We identified three major, distinct cell populations. First, *inactive* cells (*EYFP*-, *dmCherry*-) displayed no *Shox2* expression at E11.5 but expressed non-mesenchymal (*Krt5*, *Wnt6*, *Chd5*, *Myod1*) and mesenchymal (*Twist1, Msx1*) markers with a distal identity *(Hoxd13)* (Fig. 2F, G, Supplementary Data 4). Second, *maintaining* cells (*EYFP*+, *dmCherry*+) expressed high levels of *Shox2* and displayed a mesenchymal identity signal already at E11.5 (*Prrx1*, *Twist1*). Third, *decommissioned* cells (*EYFP*+, *dmCherry*-) expressed a residual level of *Shox2* and bear a mesenchymal identity (Fig. 2F, G, Supplementary Data 4). We also identified three minor cell populations. First, dmCherry-only cells, which were classified as blood cells (Fig. 2F, G). The second group, termed “low-EYFP,” exhibited low levels of *dmCherry* and *EYFP*, weak *Shox2* expression, and a mesenchymal progenitor identity (*Msx1*) (Fig. 2F, G). The third group, termed “intermediate,” displayed high EYFP and intermediate *dmCherry* levels, expressed *Shox2* and had a mesenchymal (*Prrx1*) identity (Fig. 2F, G). We did not find a distinct group of *Shox2*-*initiating* cells, suggesting they may be dispersed among the identified populations due to delayed *dmCherry* and *EYFP* translation.

Since *inactive*, *maintaining*, and *decommissioned* cell populations made up 83–95% of the cells analyzed, we focused on these three groups (Fig. 2H, Supplementary Fig. 4B, Supplementary Data 4). *Inactive* cells made up 56% of forelimb and 73% of hindlimb cells at E10.5, initially showing a proximal (*Hoxa11*+, *Hoxd13*−) and progenitor (*Irx3*+, *Msx1*+) identity. As development progressed, *inactive* cells dropped to 50%, shifting to a distal identity (*Hoxa11*−, *Hoxd13*+) with increased expression of chondrogenic and digit markers (*Col9a2*+, *Irx1*+). *Maintaining* cells made up 20% of forelimb and 4% of hindlimb cells at E10.5, increasing to 25–30% at E11.5–E12.5, then decreasing to 15% at later stages. These cells expressed proximal markers (*Hoxa11*+) and differentiated into chondrogenic (*Col9a3*+, *Acan*+) and connective tissue (*Osr1*+, *Lum*+, *Col3a1*+) lineages. Finally, *decommissioned* cells are rare at E10.5 (10% forelimb, 5% hindlimb) with a proximal progenitor identity and later differentiated into connective tissue and cartilage (*Lum*+, *Col9a2*+), in both distal and proximal segments (*Hoxa11*+, *Hoxd13*+, *Irx1*+) (Supplementary Fig. 4B). Of note, the higher proportion of *maintaining* cells in early forelimbs vs hindlimbs reflects their developmental advance. Moreover, discrepancies with single-cell analysis (Supplementary Fig. 3E) likely stem from the inherent FACS technique sensitivity. These results show that *Shox2* regulatory *initiation* depletes *inactive* cells while increasing the *maintaining* and *decommissioned* ones, and that early *Shox2* regulatory *decommissioning* coincides with the emergence of distal limb progenitors.

### *Shox2* regulatory *maintenance* is associated with distinct enhancer repertoires acting over time

**Fig. 3:**
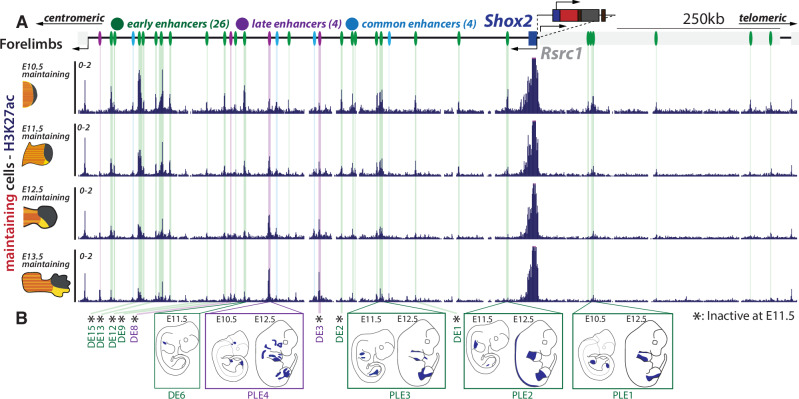
Regulatory *maintenance* associated with changes in enhancer repertoires. **A** H3K27ac ChIP-seq profiles at the *Shox2* locus (mm39: chr3:66,190,000-67,290,000) of FACS-sorted *maintaining* (dmCherry+/EYFP+) cells across E10.5, E11.5, E12.5, and E13.5 forelimbs. Putative enhancers are shown by color-coded lines: green for early, light blue for common, and purple for late enhancers, as detailed in Supplementary Data 5. The light gray box next to the *Shox2* gene is the *Rsrc1* gene. Centromeric and telomeric arrows indicate chromosomal directions. **B** Schematic representation of the pattern displayed by enhancers previously validated through in vivo LacZ reporter assays^8,9^.

Both single-cell analyses and RNA-seq data from FACS experiments have demonstrated dynamic changes in the limb cell types in which *Shox2* is transcriptionally active, progressing from limb progenitors to chondrogenic and connective cell types (see Fig. 2). To support its transcription through these transitions and adapt to this changing environment, the *Shox2* regulatory landscape likely employs distinct sets of enhancers (see Fig. 1). To delineate the stage-specific enhancer repertoires of *Shox2*, we leveraged our reporter system to focus on the chromatin status of *Shox2 maintaining* cells. We generated H3K27ac ChIP-seq profiles, an active enhancer associated mark, in E10.5, E11.5, E12.5, E13.5 forelimbs *maintaining* FACS-sorted cells (dmCherry+, EYFP+) (Fig. 3A)^38^. Here, we identified 34 H3K27ac-marked putative enhancers within the *Shox2* TAD (mm39, chr3:66190000-67290000). While a majority of these enhancers displayed an activity enriched to early stages of limb development (early enhancers: 26/34), others showed consistent H3K27ac coverage across all stages (common enhancers: 4/34) or were enriched to late stages (late enhancers: 4/34) (Fig. 3A, Supplementary Data 5). H3K27ac ChIP-seq profiles from E11.5, E12.5, and E13.5 hindlimbs *maintaining* cells showed an identical distribution of enhancers and a highly similar temporal restriction of their activities (Supplementary Fig. 5). The difference in the number of enhancers identified compared to the bulk analysis in Supplementary Fig. 1B likely originates from the increased H3K27ac enrichment in sorted cells. Additionally, the shift of category of some of the enhancers is likely due to the use of four data points in this sorted-cell analysis compared to two in the bulk analysis (see “Methods”). Prior studies have tested 13 of these enhancers in vivo, with five driving limb reporter activity at specific embryonic stages, validating our approach for enhancer identification (Fig. 3B, Supplementary Data 5)^8,9^. These findings suggest that a high number of early enhancers is required to initiate and maintain *Shox2* transcription during the early stages of limb budding, while fewer, including late enhancers, are necessary to sustain expression at later stages.

### The 3D locus topology of *Shox2* mirrors temporal enhancer repertoire shifts

**Fig. 4:**
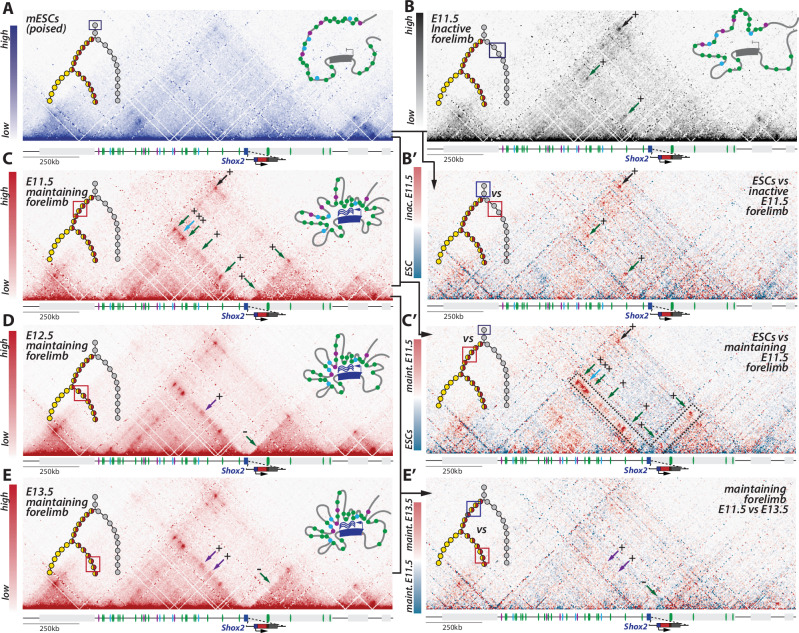
*Shox2* locus 3D topology associates with active enhancer–promoter interactions in forelimbs. In all Capture-HiC (C-HiC) maps (mm39: chr3:65,885,132-67,539,263), the upper left illustration represents the position of the investigated cells in the regulatory trajectory, and the upper right one a model of the average 3D locus structure. The light gray box next to *Shox2* is the *Rsrc1* gene. **A** C-HiC maps of the *Shox2* locus in *Shox2*^*trac*^ mESCs. Note a large TAD with few focal interaction points. **B** C-HiC maps of the *Shox2* locus in E11.5 forelimb FACS-sorted *inactive* cells. Note the formation of specific contacts with two early enhancers (green arrows; as defined with vC in Supplementary Fig. 7) and increased loop contact between the two TAD borders (black arrow). **B’** Subtraction C-HiC map between *Shox2*^*trac*^ E11.5 forelimb FACS-sorted *inactive* cells and *Shox2*^*trac*^ mESC. **C-E** C-HiC maps of the *Shox2* locus in **C** E11.5, **D** E12.5, and **E** E13.5 forelimb FACS-sorted *maintaining* cells. **C’** C-HiC subtraction maps between *Shox2*^*trac*^ mESCs and *Shox2*^*trac*^ E11.5 forelimb FACS-sorted *maintaining* cells. **E’** C-HiC subtraction maps between E12.5 and E13.5 *Shox2*^*trac*^ FACS-sorted forelimb *maintaining* cells. Changes in enhancer-*Shox2* interactions are marked by colored arrows at each stage: green for early enhancers, purple for late enhancers, and light blue for common enhancers (as defined with vC in forelimb and hindlimb: see Supplementary Fig. 7). A plus sign (+) denotes a gain of interaction, and a minus sign (−) indicates a loss of interaction relative to the previous stage. Also note the increased separation between the two subTADs at the position of the *Shox2* gene body. Maps coordinates mm9; chr3:65,781,633-67,435,852. Maint. maintaining, inac. inactive.

Recent studies have demonstrated that changes in chromatin architecture associate with the activity of enhancers and promoters^2,55^. We sought to investigate whether the temporal shifts in enhancer activities observed at the *Shox2* locus are also associated with temporal changes in the 3D chromatin organization. To tackle this question, we generated capture-HiC (C-HiC) maps across different phases and stages of the *Shox2* regulatory trajectory. We started by comparing *Shox2*^*trac*^ embryonic stem cells (mESCs) with *inactive* (*dmCherry*-, *EYFP*-) fore- and hindlimb FACS-sorted cells at E11.5. Notably, while the poised *Shox2* locus in mESCs^39^ exhibited a relatively relaxed structure, with few focal interactions, *inactive* cells displayed increased contacts between the TAD boundaries and between *Shox2* and three of its early enhancers (Fig. 4A, B, B’, Supplementary Fig. 6). Using Virtual capture-HiC (vC) we saw that the gain of enhancer interactions precisely occurred with the *Shox2* promoter (Supplementary Fig. 7). We then studied the transition from a poised state towards an active one, by comparing *Shox2*^*trac*^ mESCs and fore- and hindlimb FACS-sorted *Shox2 maintaining* cells at E11.5, E12.5, and E13.5. We observed in E11.5 *maintaining* cells a gain of interactions originating from *Shox2* throughout its entire TAD reflected by an increased contact stripe (Fig. 4A, C–E, C’ and Supplementary Fig. 6). VC analyses revealed that this increase was more pronounced at early and common enhancers, but not at late ones (Supplementary Fig. 7). This was accompanied by a pronounced segregation of the locus into two subTADs in-between the *Shox2* and *Rsrc1* promoters (Fig. 4A, C–E, C’ and Supplementary Fig. 6). Although the maps of *maintaining* cells of the three investigated stages were quite similar among each other, subtle but consistent interaction changes were visible (Fig. 4C–E, E’). We indeed noted that one early enhancer showed decreased interactions with *Shox2* at E13.5, while two late enhancers showed increased interactions (Fig. 4C–E, E’, Supplementary Figs. 6 and 7). Thus, these observations show that the 3D structure of the *Shox2* locus accompanies a shift between early and late enhancer repertoires.

### *Shox2* enhancers act in a stage-specific manner to control *Shox2initiation* and *maintenance*

**Fig. 5:**
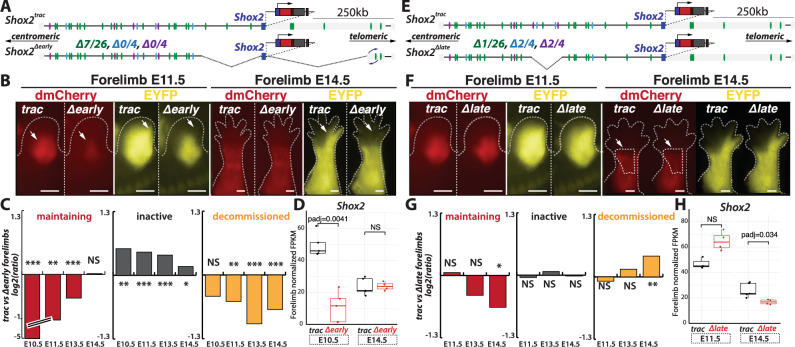
Partial early and late enhancer deletion induces stage-specific alterations. **A** Illustration detailing the *Shox2*^*Δearly*^ deletion allele, in cis with the mCherry-P2A-CRE cassette, lacking 7 out of 26 early putative enhancers (in green), while late (in purple) and common (light blue) putative enhancers remain intact. Centromeric and telomeric arrows indicate chromosomal directions. **B** Fluorescent imaging comparing *Shox2*^*trac*^ and *Shox2*^*Δearly*^ forelimbs at E11.5 and E14.5 (scale bars: 500 μm). White arrows indicate a reduction in dmCherry and EYFP signals at E11.5 and a complete loss of EYFP signal in distal limbs at E14.5. In total, 10 *Shox2*^*trac*^ E11.5, 3 *Shox2*^*Δearly*^ E11.5, 10 *Shox2*^*trac*^ E14.5, 3 *Shox2*^*Δearly*^ E14.5 embryos were imaged and showed similar patterns. **C** Log2 ratio of the proportion of *maintaining*, *inactive*, and *decommissioned* cell populations, identified by flow cytometry analyses, in *Shox2*^*trac*^ versus *Shox2*^*Δearly*^ forelimbs at E10.5, E11.5, E13.5, and E14.5. Source data are provided in the Source data file and analyses in Supplementary Data 6. **D** Normalized FPKM of *Shox2* expression in E10.5 and E14.5 *Shox2*^*trac*^ and *Shox2*^*Δearly*^ entire forelimbs, with comprehensive DESeq2 analyses in Supplementary Data 7. Source data for this plot are provided in the Source data file. (*N* at E10.5 *Shox2*^*trac*^ = 5, *N* at E10.5 *Shox*2 ^*Δearly*^ = 5, *N* at E14.5 *Shox2*^*trac*^ = 5, *N* at E14.5 *Shox2* ^*Δearly*^ = 4). **E** Schematic representation of the *Shox2*^*Δlate*^ deletion allele lacking 2 out of 4 late (in purple), 2 out of 4 common (in light blue), and 1 out of 26 early putative enhancers. **F** Fluorescent imaging of *Shox2*^*trac*^ and *Shox2*^*Δlate*^ forelimbs at E11.5 and E14.5 (scale bars: 500 μm). White arrows and delimited area indicate a loss of dmCherry signal in the central limb section at E14.5. In total, 10 *Shox2*^*trac*^ E11.5, 4 *Shox2*^*Δlate*^ E11.5, 10 *Shox2*^*trac*^ E14.5, 9 *Shox2*^*Δlate*^ E14.5 embryos were imaged and showed similar patterns. **G** Log2 ratio of the proportion of *maintaining*, *inactive*, and *decommissioned* cell populations, identified by flow cytometry analyses, *Shox2*^*trac*^ versus *Shox2*^*Δlate*^ forelimbs at E11.5, E13.5, and E14.5. Source data are provided in the Source data file and analyses in Supplementary Data 6. **H** Normalized FPKM of *Shox2* in E10.5 and E14.5 *Shox2*^*trac*^ and *Shox2*^*Δlate*^ entire forelimbs, detailed DESeq2 analyses in Supplementary Data 7. Source data for this plot are provided in the Source data file. (*N* at E11.5 *Shox2*^*trac*^ = 3, *N* at E11.5 *Shox2*^*Δlate*^ = 4, *N* at E14.5 *Shox2*^*trac*^ = 5, *N* at E14.5 *Shox2* ^*Δlate*^ = 4). In (**C**, **G**): two-sided *t*-tests were utilized to calculate *p* values from replicates (Supplementary Fig. 9). NS non-significant, *p < 0.05, **p < 0.01, and ***p < 0.001. In (**D**, **H**) boxplots, boxes indicate the first and third quartiles, the whiskers indicate ±1.5× interquartile range, and the horizontal line within the boxes indicates the median. Statistical test used: DESeq2 Wald test, *p*adj is the FDR-corrected *p* value from DESeq2.

To functionally assess the role, timing and interdependency of identified early and late enhancers, we investigated how deletions of these enhancers affect the reporter system as well as *Shox2* expression. We first assessed the role of several early enhancers by generating two consecutive deletions, one 172 kb on the centromeric side of *Shox2* and another of 308 kb on its telomeric side, in the *Shox2*^*trac*^ background, solely removing early-acting enhancers, while leaving the common and late enhancers intact (Fig. 5A). Because of the size of this double deletion we produced C-HiC in deleted and control E12.5 forelimbs to assay possible changes in chromatin topology. While we did not notice any unexpected change in the TAD borders or in enhancer–promoter interactions, we noted that the telomeric 308 kb deletion breakpoint *in cis* with the dmCherry-P2A-CRE cassette allele retained an inverted 44 kb fragment at its most telomeric breakpoint (Fig. 5A, Supplementary Fig. 8A, B). As a result, the dmCherry-P2A-CRE reporter allele was associated with a loss of 7 early enhancers, while the other *Shox2* allele lost 8 early enhancers. This deletion allele was referred to as *Shox2*^*Δearly*^ *(Shox2*^*dmCherry;Δearly264kb/+;Δearly308kb*^; *Rosa26*^*loxEYFP/+*^*)* and examined using fluorescent imaging, quantification of the proportion of *inactive*, *maintaining*, and *decommissioned* cells, as well as RNA-seq. At E11.5 *Shox2*^*Δearly*^ embryos displayed a limb-wide decreased in dmCherry and EYFP signals compared to control *Shox2*^*trac*^ forelimbs (Fig. 5B). We also observed the loss of the distal limb EYFP-only signal. By E14.5, dmCherry changes were no longer apparent, but the loss of EYFP signal in distal segments persisted. Quantification of cell proportions from flow cytometry experiments in *Shox2*^*Δearly*^ fore- and hindlimbs at E10.5, E11.5, E13.5, and E14.5 showed a complete loss of *maintaining* cells at E10.5 (33 fold less, from 19% in *Shox2*^*trac*^ forelimb cells to 0.6% in *Shox2*^*Δearly*^) that gradually returned to control levels at later stages (Fig. 5C, Supplementary Figs. 9A, B and 10A, B, Supplementary Data 6). Additionally, we observed an increase in *inactive* cells, especially at early stages (1.5 fold more: from 56% in *Shox2*^*trac*^ forelimbs to 81% in *Shox2*^*Δearly*^), implying the inability of these cells to initiate the gene transcription. Concomitantly, we noted a decrease in *decommissioned* cells reflecting the observed loss of distal EYFP signal (for instance at E13.5 2 fold less: from 23.3% in *Shox2*^*trac*^ forelimbs to 11.7% in *Shox2*^*Δearly*^, Fig. 5B, C, Supplementary Fig. 9A, B, Supplementary Data 6). RNA-seq of E10.5 and E14.5 forelimbs revealed a decrease in *Shox2* expression only at E10.5, aligning with the changes observed in dmCherry fluorescence (Fig. 5D, Supplementary Data 7). These findings indicate that the removal of early enhancers leads to the transient inability of cells to initiate *Shox2* transcription in early limb progenitor cells. While transcription in more proximal lineages is gradually restored by the remaining enhancers, cells destined for distal posterior limb segments do not regain *Shox2* expression (see Fig. 2).

Conversely, we hypothesized that late enhancers, in conjunction with common ones, are crucial for maintaining *Shox2* expression during the late stages of limb development, without significantly contributing to the early phase. To test this hypothesis, we engineered a targeted deletion spanning 84 kb, *Shox2*^*Δlate*^ *(Shox2*^*dmCherry/+;Δlate/Δlate*^; *Rosa26*^*loxEYFP/+*^*)*, that eliminates 2 out of 4 late enhancers (50% of late), 2 out of 4 common enhancers (50% of common) and 1 out of 26 early enhancers (4% of early) (Fig. 5E). At E11.5, no discernible differences in dmCherry or EYFP fluorescence were observed in forelimb tissues compared to controls (Fig. 5F). However, a slight decrease in dmCherry signal (but not EYFP) was noted in the central section of E14.5 forelimbs (Fig. 5F). Flow cytometry analyses of cell proportions in *Shox2*^*Δlate*^ forelimbs at E11.5, E13.5, and E14.5 revealed a specific reduction in *maintaining* cells solely at E14.5 (1.6 fold less: from 15% in *Shox2*^*trac*^ to 10% in *Shox2*^*Δlate*^, Fig. 5G, Supplementary Fig. 9C, Supplementary Data 6). RNA-seq analysis at E11.5 and E14.5 confirmed a loss of *Shox2* expression exclusively at E14.5 (Fig. 5H, Supplementary Data 7). Unlike the early enhancer deletion, the late enhancer deletion did not impact the proportion of *inactive* cells but only led to an increase in *decommissioned* cells (1.3 fold more: from 22% in *Shox2*^*trac*^ to 30% in *Shox2*^*Δlate*^), indicating that previously ongoing transcription was halted due to the absence of late regulatory elements, entering in a premature *decommissioning* phase (Fig. 5G, Supplementary Fig. 9C, Supplementary Data 6). Consistent with this, we observed lower *Shox2* expression in sorted *maintaining* and *decommissioned* cells of *Shox2*^*Δlate*^ compared to *Shox2*^*trac*^ E14.5 forelimbs, further supporting the requirement of late enhancers to maintain high expression levels (Supplementary Data 7). Of note, although the effects measured by bulk and sorted-cell RNA-seq as well as flow cytometry are of relatively small magnitude, all approaches consistently demonstrate a significant loss of late-stage regulatory activity in the limb. In contrast, hindlimb analyses showed no significant changes, possibly due to the developmental delay between fore- and hindlimbs (Supplementary Fig. 10C, D, Supplementary Data 6). Together, these deletion experiments demonstrate that *Shox2* transcription is regulated by different enhancer repertoires operating transiently, in a temporally specific manner during limb development. Moreover, it shows that as early enhancers induce transcription from a transcriptionally inactive locus, late enhancers act to maintain transcription in cells with ongoing transcription.

### Enhancer–promoter disconnection marks *Shox2decommissioning*

**Fig. 6:**
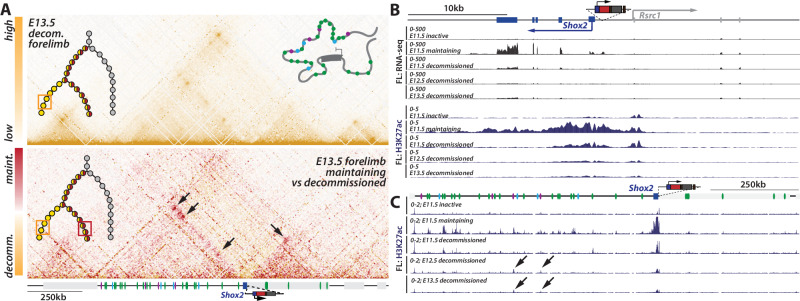
*Shox2* locus *decommissioning* associated with the disconnection between enhancers and promoters. **A** C-HiC of E13.5 forelimb FACS-sorted *decommissioned* cells (above) and subtraction map with E13.5 forelimb FACS-sorted *maintaining* cells (See Fig. 5C); in each panel, the upper left illustration represents the position of the investigated cells in the regulatory trajectory; the upper right one is a model of the average 3D locus structure (maps coordinates mm39; chr3:65,885,132-67,539,263). Black arrows indicate strong losses of enhancer–promoter interactions **B** RNA-seq and H3K27ac ChIP-seq tracks of early (E11.5) forelimb *inactive*, *maintaining*, *decommissioned*, and late (E12.5-E13.5) *decommissioned* FACS-sorted cells at the *Shox2* and *Rsrc1* promoter region (mm39: chr3:66,870,000-66,910,000). **C** Forelimb H3K27ac ChIP-seq tracks in early (E11.5) forelimb *inactive*, *maintaining*, *decommissioned*, and late (E12.5-E13.5) *decommissioned* FACS-sorted cells over the *Shox2* regulatory landscape (mm39: chr3:66,190,000-67,290,000). Note the overall loss of H3K27ac at enhancers in *decommissioned* cells while two of the four late enhancers show activity in *decommissioned* cells (black arrows).

Despite their fundamental contribution to the formation of gene expression patterns, the features associated with the termination of a locus’ transcriptional activity remain poorly characterized. To define how *Shox2* transcription is terminated, we examined the changes in locus topology, transcription, and activities of regulatory elements within *decommissioned* cells. First, we generated C-HiC maps of FACS-sorted *decommissioned* cells from *Shox2*^*trac*^ E12.5 and E13.5 fore- and hindlimbs. Compared to stage-matched *maintaining* cells, we observed a notable reduction in enhancer–promoter interactions and a decrease in the segregation of the two subTADs (Fig. 6A, Supplementary Fig. 11A–C). We noticed that this *decommissioned* structure was very similar to the one seen in *inactive* cells (as shown in Fig. 4B, Supplementary Fig. 11D). Looking at the locus regulatory activity, we observed a significant decrease in *Shox2* expression and H3K27ac coverage of the gene’s promoter (Fig. 6B, Supplementary Fig. 12A). At the enhancer level, we noted a depletion of H3K27ac coverage, especially visible at early and common enhancers (Fig. 6C, Supplementary Fig. 12B). Intriguingly, by the late E13.5 stage, two late enhancers retained H3K27ac coverage in *decommissioned* cells. The same regions exhibited some activity in late E12.5 and E13.5 *inactive* cells as well (Supplementary Fig. 12C). This suggests that these two enhancers, although significantly contributing to *Shox2* *maintenance* (see Fig. 5E–H), are insufficient to initiate or maintain *Shox2* expression by themselves. We also noted that in distal limbs, despite the absence of *maintaining* cells and the presence of approximately one third of *decommissioned* cells, the ATAC-seq open chromatin profile of the locus closely resembled that of transcriptionally-active proximal limbs (Supplementary Fig. 12D)^56^. This suggest that *Shox2* enhancers stay open irrespective of *Shox2* locus transcriptional state in the limb.

As regulatory activity diminishes, the emergence of facultative heterochromatin could drive locus repression. To investigate this, we assessed the coverage of the PRC2-associated H3K27me3 repressive mark across different cell categories. In E13.5 hindlimbs, we found that the *Shox2* promoter was covered by H3K27me3 in *inactive* cells, depleted in *maintaining* cells, and displayed only weak enrichment in *decommissioned* cells (Supplementary Fig. 12E). This suggests that regulatory *decommissioning* results from the shutdown of enhancer activities rather than from PRC2-mediated repression at enhancers or promoter. Taken together, these findings indicate a disconnection of the majority of enhancer–promoter contacts, accompanied by a reduction in their active chromatin coverage during locus *decommissioning*.

### An *Ulnaless-*like allele triggers ectopic *Shox2* locus *decommissioning*

The rapid disconnection of enhancer–promoter contacts and the decline in enhancer activities upon *Shox2* locus *decommissioning* imply a targeted repressive activity at enhancer regions, potentially mediated by the binding of specific TFs. Furthermore, the presence of many *decommissioned* cells in the distal limb domain suggests the involvement of distal limb TFs in this process (Fig. 2). HOXA/D13 TFs have been involved in *Shox2* repression in distal limbs, and both factors are binding several identified *Shox2* enhancers (Supplementary Fig. 13A)^57,58^. Notably, out of 34 *Shox2* putative limb enhancers, HOXA/D13 proteins were found to bind to 16 of them in distal forelimbs at E12.5^57^. This positions HOXA/D13 as candidate TFs for controlling *Shox2* regulatory *decommissioning* by inhibiting its enhancers in distal limb progenitors.

**Fig. 7:**
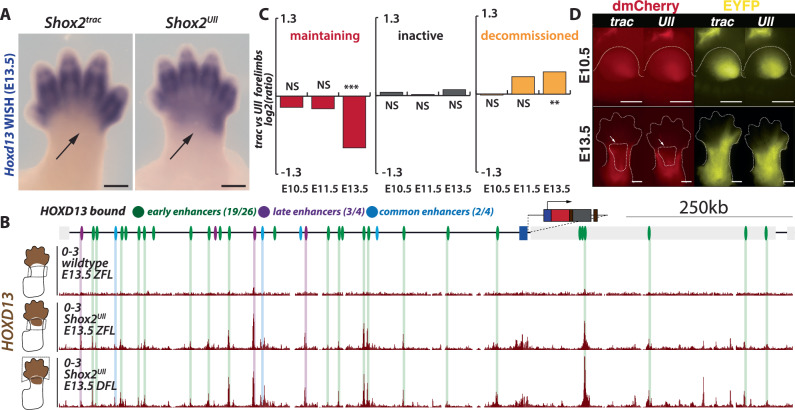
HOXD13 gain-of-function suggests it can induce *Shox2* locus *decommissioning*. **A** *Hoxd13* WISH in control and *Shox2*^*Ull*^ (*Ulnaless*-like) forelimbs at E13.5 (scale bars: 500 μm, 4 wildtype and 2 *Shox2*^*Ull*^ embryos were used). Note how the *Ulnaless*-like inversion allele induces proximal expression of *Hoxd13* in forelimbs (black arrow). **B** HOXD13 CUT&RUN in wildtype, *Shox2*^*Ull*^ E13.5 zeugopod forelimb (ZFL) and distal forelimb (DFL). **C** Log2 ratio between the proportion of forelimb *Shox2*^*trac*^ and *Shox2*^*Ull*^, *initiating*, *maintaining*, *inactive* and *decommissioned* cell populations, identified by flow cytometry at E10.5, E11.5, and E13.5. Two-sided *t*-tests were utilized to calculate *p* values from replicates (See supplementary Fig. 13). NS non-significant, *p < 0.05, **p < 0.01, and ***p < 0.001. Source data are provided in the Source data file and analyses in Supplementary Data 6. **D** Fluorescent imaging of *Shox2*^*trac*^ and *Shox2*^*Ull*^ forelimbs at E10.5 and E13.5 (scale bars: 500 μm). Note the decreased dmCherry signal in the central part of the limb (white arrow). Maint. maintaining, decomm. decommissioned. In total, 7 *Shox2*^*trac*^ E10.5, 3 *Shox2*^*Ull*^ E10.5, 11 *Shox2*^*trac*^ E13.5, 10 *Shox2*^*Ull*^ E13.5 embryos were imaged and showed similar patterns.

To explore this hypothesis, we employed a limb-specific gain-of-function allele known as *Ulnaless*, which induces *Hoxd13* expression in proximal limbs in a domain partially overlapping the one of *Shox2*^59,60^. Here, we re-engineered the *Ulnaless* inversion (*Ulnaless-like (Ull)*) in the dual-color tracking background: *Shox2*^*Ull*^ (*Shox2*^*dmCherry/+;*^; *Rosa26*^*loxEYFP/+*^; *HoxD*^*Ull/+*^) (Supplementary Fig. 13B). As expected, *Shox2*^*Ull*^ displayed a gain of *Hoxd13* transcripts in the proximal region of E13.5 forelimbs (Fig. 7A). We could confirm this gain of expression using RT-qPCR upon micro-dissection of the zeugopod forelimbs (ZFL) (Supplementary Fig. 13C). We further investigated the binding patterns of HOXD13 at the *Shox2* locus in ZFL using CUT&RUN. Unlike in wildtype ZFL, where no HOXD13 binding was detected, in *Shox2*^*Ull*^ ZFL, HOXD13 was found pervasively binding the genome, including to 24 of the 34 *Shox2* enhancers, without preference for early-, common- or late enhancers (Fig. 7B). Although the CUT&RUN pattern of HOXD13 binding differed slightly from the ChIP-seq reported by ref. ^57^ it matched the CUT&RUN pattern observed in distal *Shox2*^*Ull*^ forelimbs (DFL), reflecting differences in limb stages, antibody affinity and technique (Fig. 7B). Moreover, the similarity in HOXD13 binding between *Shox2*^*Ull*^ ZFL and DFL demonstrates its ability to bind the same genomic regions in both limb contexts.

If *Hoxd13* acts as a decommissioning factor, we would expect to observe a decrease in *maintaining* cells and an increase in *decommissioned* cells, without affecting *inactive* cells. Quantification of such cell proportion by flow cytometry in both fore- and hindlimbs precisely revealed a decrease in *maintaining* cells (1.8 fold less, from 21% of forelimb cells in *Shox2*^*trac*^ to 12% in *Shox2*^*Ull*^) and an increase in *decommissioned* cells (1.3 fold more, from 23% in *Shox2*^*trac*^ forelimb to 30% in *Shox2*^*Ull*^) at the late E13.5 stage, but not at earlier stages (Fig. 7C, Supplementary Fig. 13D–F). Additionally, fluorescent microscopy revealed a mild reduction of dmCherry signal in the center of E13.5 forelimbs but not at E10.5 (Fig. 7D). These findings mirror the effects observed by late enhancer loss (See Fig. 5) and demonstrate that HOXD13 can induce locus *decommissioning*, likely through binding active enhancers, promoting the transition of *maintaining* cells into the *decommissioning* pool without affecting the proportion of *inactive* cells.

## Discussion

Developmental genes exert their influence through spatially and temporally restricted expression patterns^61–63^. Building upon previous studies on enhancer dynamics, which explored enhancer activation and decommissioning, we investigated the behavior of an entire gene regulatory landscape over time^15,64,65^. Using *Shox2* as a model, we show that early limb cells either remain inactive, contributing mainly to distal limb populations, or become transcriptionally active in limb progenitors, with expression maintained in differentiating proximal connective tissue and cartilage. After *initiation* and *maintenance*, some cells, including those contributing to distal limb segments, undergo *Shox2* regulatory *decommissioning*, possibly driven by distally expressed TFs such as HOXA/D13.

In mammals, several studies have identified stage-specific enhancers^11–14^. Here, we show that such enhancers are widely used during limb bud patterning and explore their functional role in shaping gene expression in vivo using a transgenic recorder approach. We classify limb enhancers into three categories, early, common, and late, based on their H3K27ac dynamics. While many of these stage-specific regions exhibit enhancer activity, some may represent other types of regulatory elements^66,67^. Stage specificity may arise from an enhancer’s intrinsic activation timing in response to signaling or an activity limited to cell types that are themselves temporally restricted during development. Unlike common enhancers, which likely contribute to transcription consistently, early and late enhancers appear to have distinct roles.

Early enhancer activity is likely restricted to progenitor cells present during early limb development or responsive to transient signaling cues, such as retinoic acid^68^. Deletion experiments demonstrate that these enhancers initiate *Shox2* expression in early limb development, resembling the initiator elements described at the BX-C locus in *Drosophila*^15–17^. Yet, despite the significant delay and loss of *Shox2* expression following early enhancer deletion, late expression is eventually restored, suggesting that remaining early, common, and late enhancers sustain *Shox2* expression at later stages. However, whether this restoration results from a collective effect of all enhancer classes or specific ones, and whether late enhancers alone can compensate for early enhancer loss, remains unresolved.

The deletion of 50% of late and common enhancers led to an increase in *decommissioned* cells at later stages without affecting *inactive* cell proportions, contrasting sharply with early enhancer deletions. This indicates that late and/or common enhancers function to maintain expression in a previously activated *Shox2* locus, akin to maintenance elements described at the BX-C locus in *Drosophila*^15–17^, rather than initiating transcription in *inactive* cells. This is further supported by the presence of H3K27ac on late enhancers in *inactive* cells, suggesting that these enhancers act only in cells already imprinted with early enhancer-driven patterning cues. Since *Drosophila* maintenance elements are cell-type specific, a conserved mechanism would suggest that late limb enhancers sustain transcription only in specific daughter cells.

After regulatory active phases, gene expression can shut down to establish sharp expression borders critical for defining anatomical transitions. In the case of *Shox2*, its widespread expression in the early limb bud becomes restricted to proximal limb segments, with a sharp expression boundary at the mesopodium level opposing the one of *Hoxa/d13*^33^. In *Ulnaless-like* embryos, we observed premature *Shox2* *decommissioning*, correlating with the proximal shift of *Hoxd13* expression (Fig. 7A) and the known role of HOXA/D13 in repressing *Shox2*^58^. Our CUT&RUN data shows that HOXD13 binds *Shox2* enhancers in both its ectopic zeugopod and normal distal domains, suggesting it could repress the locus via an enhancer decommissioning mechanism^65^. Similar to pluripotency exit, where FOXD3 recruits LSD1 and HDACs to dismantle active pluripotency enhancers through H3K4me2 demethylation and H3K27ac deacetylation^64,69^, HOXA/D13 proteins may recruit these factors to decommission *Shox2* enhancers in distal limbs. This could lead to the disassembly of its 3D structure and loss of enhancer–promoter interactions observed in *decommissioned* cells.

Our tracking approach and early enhancer deletion reveal that early *Shox2* *decommissioned* cells contribute to distal limb segments, including the mesopodium and *Hoxd13*-expressing digit progenitors. This early-limb cell population co-expresses limb progenitor (*Msx1*), proximal (*Irx3/5*), and distal (*Shh*) markers resembling a recently described limb progenitor population co-expressing proximal and distal markers^24,33,70–72^. Therefore, this conflicting identity of early *decommissioned* cells is likely to extend to other proximodistal markers and suggests that early repression of proximal gene activity by distal-expressed factors could be a central mechanism for limb distalisation.

At the onset of *Shox2* transcription, enhancer–promoter contacts increase sharply, coinciding with the formation of a subTAD boundary at the *Shox2* promoter. As development progresses, interactions shift from early to late enhancers, reflecting their sequential activities. Finally, during *decommissioning*, enhancers disconnect from *Shox2* alongside a reduction in H3K27ac coverage, a process previously observed in cell differentiation assays^73,74^. This dynamic structure is unlikely to be driven by CTCF, as the protein does not bind the *Shox2* promoter at any stage and shows only minimal binding differences in the centromeric regulatory landscape (Supplementary Fig. 14)^37^. Instead, two CTCF-independent mechanisms may explain this dynamic locus topology. One possibility, as recently proposed, is that cohesin is loaded onto enhancers via RNAPII and extrudes the local domain until reaching the *Shox2* promoter, forming a self-reinforcing structural loop^75^. Alternatively, the contact might be independent of cohesin and the loop extrusion process, but relying on the recruitment of looping factors at the various active enhancers, as recently shown in a growing number of reports^76–79^.

Overall, our findings describe the existence of temporally restricted enhancer repertoires with distinct *initiating* or *maintaining* regulatory functions, as well as their synchronized shutdown during locus *decommissioning*, and the significant structural transformations accompanying these regulatory events. These insights are potentially applicable to dozens of other limb-associated loci (see Fig. 1) and are likely extendable to genes involved in other morphogenetic processes.

### Limitations of the study

A key challenge in this study was the inability to sort a distinct *initiating* population, likely due to the very short time window during which dmCherry is expressed and the floxed STOP site has not yet been removed. This issue prevented a detailed analysis of the *Shox2* regulatory landscape during the *initiation* phase and hindered determining the precise order of activation of early enhancers, which have the capacity to initiate transcription. Therefore, further investigations are necessary to establish whether *Shox2* regulatory *initiation* is influenced by all identified enhancers or only a subset.

## Methods

### Animal procedures

Animal work performed in Geneva complied with all relevant ethical regulations of the University of Geneva and followed procedures approved by the animal care and experimentation authorities of the Canton of Geneva, Switzerland (animal protocol numbers GE/89/19 and GE192A).

### CRISPR/Cas9 engineered alleles

Alleles genetically engineered were edited using CRISPR/Cas9 and following a similar procedure as ref. ^80^. In brief, sgRNAs were designed using the online software Benchling (https://benchling.com/) and were chosen based on predicted on-target and off-target scores. All the sgRNAs used for this study, their CRISPR/Cas9 genomic target location, and genotyping primers can be found in Supplementary Data 8. Each sgRNA was cloned into the plasmid pX459 (Addgene, #48139), and 8 µg of each vector was used during mESCs transfection following the standard procedure for mESCs culture and genomic editing^81^. To construct the *Shox2*^*dmCherry/+;RosaEYFP/+*^ or *Shox2*^*trac*^ mESC clone, two rounds of targeting on G4 male cells^82^, obtained from the Nagy laboratory (http://research.lunenfeld.ca/nagy/?page=mouse%20ES%20cells), were performed, first to insert the dmCherry-CRE cassette at the *Shox2* locus (*Shox2*^*dmCherry*/+^), followed by a second round of targeting to insert the EYFP cassette at the *ROSA26* locus (*Shox2*^*trac*^*)*. To integrate the dmCherry-CRE cassette, cells were transfected with 8 µg of the corresponding sgRNA and 4 µg of an in-house-designed cassette containing unbalanced homology arms (1.7 kb and 0.5 kb), minimal β-globin promoter, dmCherry reporter sequence, PEST sequence, P2A ribosomal skipping sequence, CRE protein sequence, and a bGH-PolyA terminator. To integrate the EYFP cassette, the EYFP sensor from ref. ^47^ was modified by shortening the homology arms (final length of unbalanced arms 1.4 kb and 0.85 kb) and by removing the PGK promoter, the Neo/Kan selection cassette, the polyA of the PGK, and the first spacer before the first SV40 poly(A). In-house-designed and -modified cassettes were synthesized by Azenta Genomics/GENEWIZ. Alleles containing deletions or inversions were created in subsequent rounds of targeting performed using the *Shox2*^*trac+*^ mESC clone as the starting clone.

### Allele characterization

All alleles were genotyped by PCR (primers were designed using Primer3 software) using genomic DNA extracted from mESCs and embryos with Monarch Genomic DNA Purification Kit (#T31010L) (Supplementary Data 8)^83^.

*For the insertion* of dmCherry-P2A-CRE and floxed-SV40pASTOP-EYFP cassettes, two pairs of primers were designed at each side of the insertion breakpoint, with one primer outside the homology arm and another primer inside the cassette to confirm insertion at the locus from both sides. Non-inserted alleles were detected by PCR using the primers outside the homology arms. In the case of heterozygous insertion, both the wild-type band and the band including the cassette were extracted from the gel and sequenced.

*For deletions*, primers were designed to amplify a product only if the deletion was present, by designing two primers located at least 200 bp outside of the expected breaking point. The PCR band was sequenced to characterize the breakpoint. Additionally, each one of these primers was combined with a second primer located at least 200 bp of distance from the breaking point inside the deleted interval. With these primer combinations, wildtype fragments could be amplified at each CRISPR/Cas9 cutting site and therefore heterozygous deletions excluded. The zygosity of deletions was further confirmed using qPCR on 10 ng/µl genomic DNA with primers described in Supplementary Data 8 using Go Taq qPCR Master Mix (Promega A600A) and StepOnePlus Real-Time PCR System from Applied Biosystems.

For the *Shox2*^*Δlate*^ mutant, qPCR was performed with three primer pairs within the deleted interval, including two designed precisely at the putative *Late3* and *Late4* enhancers, confirming the homozygous loss of the 84 kb fragment.

For *the Shox2*^*Δearly*^ mutant, qPCR was performed with three primer pairs within the deleted intervals, including two designed precisely at the putative *Early 20* and *Early 25* enhancers, and showed a partial homozygous loss of the 308 kb fragment. The *Shox2*^*Δearly*^ mutant was further characterized by comparing C-HiC of *Shox2*^*trac*^ and *Shox2*^*Δearly*^ forelimbs, which revealed that the telomeric breakpoint of the expected 308 kb deletion retained a 44 kb fragment in one allele, while PCR and Sanger sequencing confirmed that the other allele carried the full deletion. To determine whether the deletion was *in cis* with the dmCherry-P2A-CRE cassette, Virtual Capture-HiC (vC) was performed following the C-HiC pipeline described in https://github.com/lldelisle/scriptsForAmandioEtAl2021/blob/main/cHi-C/ and using a customized genome assembly GRCm39/mm39^84^ incorporating the dmCherry-P2A-CRE inserted at its endogenous location at the *Shox2* locus, which we named GRCmm39/mm39_Shox2_dsmCherry_P2A_CRE_bGHpA_KI genome and that is available on https://zenodo.org/records/14865689. By running this vC approach using the cassette as viewpoint (mm39_Shox2_dsmCherry_P2A_CRE_bGHpA_KI; chr3:66890192-66892123), we confirmed the presence of the 44 kb fragment in *cis* with the cassette (See Supplementary Fig. S8B). Moreover, from the C-HiC data, we noticed that the 44 kb fragment was inverted, thereby explaining why we did not detect a wild-type PCR amplicon for the 308 kb telomeric CRISPR/Cas9 cutting site. We confirmed this observation by PCR-amplifying and sequencing the breakpoints of the inverted 44 kb sequence (Supplementary Data 8).

### Aggregation of mESCs clones and embryo collection

Mouse (*Mus musculus*) embryos were obtained following the tetraploid complementation procedure of the genetically-engineered male G4 (129/sv x C57BL/6 F1 hybrid) mESCs^48,82^. In brief, 2 days before the aggregation procedure, desired clones were thawed, seeded on male and female CD1 feeders, and grown. Donor tetraploid embryos were provided from in vitro fertilization using C57BL6J x B6D2F1 backgrounds. Aggregated embryos were transferred into CD1 or B6CBA foster females. Animals were obtained from Janvier Laboratories or from in-house crosses. Embryos were collected in 1X DPBS (Gibco, 14190-094) at the desired stage depending on the downstream protocol. The presence of desired mutations in embryos was confirmed by PCR genotyping (Supplementary Data 8).

### Embryo imaging

Embryos were imaged in 1X DPBS (Gibco, 14190-094) in a petri dish on a Zeiss Axio Zoom V16 using the Axiocam 506 color camera for brightfield images or the Axiocam 506 mono camera for fluorescent images, after fluorescent laser stimulation (Illuminator HXP 200C) using filter 46 HE YFP (excitation BP 500/25, emission BP 535/50) for capturing EYFP signal and filter 63 HE mRFP (excitation BP 572/25, emission BP 629/62) for capturing dmCherry signal. Images were taken using the Zen Blue Software v3.6. Adjustment of brightness was performed using Adobe Lightroom v6.4.

### Light sheet microscopy imaging

E12.5 embryo was fixed overnight in 4% PFA and stored in 1x PBS at 4 °C until the clearing procedure started. The entire embryo was cleared using a passive CLARITY-based clearing method. Tissue was first incubated for 3 days at 4 °C in a Bis-free Hydrogel X-CLARITY™ Hydrogel Solution Kit (C1310X, Logos Biosystems) to allow hydrogel solution diffusion into the tissue. This was followed by polymerization in a Logos Polymerization system (C20001, Logos Biosystem) at 37 °C for 3 h. The SDS-Clearing solution was prepared by dissolving 24.73 g of boric acid (Sigma B7660 or Thermofisher B3750) and 80 g of sodium dodecyl sulfate (Brunschwig 45900-0010, Acros 419530010, or Sigma L3771) in dH2O to make 2 L of 4% SDS solution, adjusting the pH to 8.5. Samples were then washed twice for 30 min in PBS, immersed in the SDS-based clearing solution at 37 °C for 48 h, followed by two PBS washes with 0.1% TritonX. Finally, tissue was placed in a Histodenz© based refractive index-matching solution (Histodenz Sigma D22158, PB + Tween + NaN3 pH 7.5 solution, 0.1% Tween 20, 0.01% NaN3, in 0.02 M phosphate buffer, final solution pH 7.5). Imaging was performed with a home-built mesoscale single-plane illumination microscope, the mesoSPIM microscope is described in ref. ^85^. In brief, the sample was excited with 488 nm, 561 nm, and 647 nm lasers. The beam waist was scanned using electrically tunable lenses (ETL, Optotune EL-16-40-5D-TC-L) synchronized with the rolling shutter of the sCMOS camera. This produced a uniform axial resolution across the field-of-view of 5 μm. EYFP signal was filtered with 530/43 nm, dmCherry signal with 593/40, and far-red signal with LP663 bandpass filter (BrightLine HC, AHF). Z-stacks were acquired at 5 μm spacing with a zoom set at ×1.25, resulting in an in-plane pixel size of 5.26 μm. Background and autofluorescence signal were subtracted using the 561 nm excitation channel during image pre-processing. This step, together with subsequent normalization and filtering of the images, was performed with the Amira 2020.1 software. 3D videos and images were captured using the Imaris 9.6.0 software.

### Whole mount in situ hybridization (WISH)

#### Probes design and production

*Hoxd13* probe was produced by amplification from mouse wildtype DNA using primers located at the 3′UTR of the desired genes. Primers were designed with Prime3 Software v 0.4.0 (using default parameters except: product size range 400–600 bp; primer size 15–19 bp; primer Tm 60–62°), extended with either SP6 or T7 primer sequence (*Hoxd13* probe forward primer: CAAGCTATTTAGGTGACACTATAGTGCTGCCCAATCCGACT; *Hoxd13* probe reverse primer: GAACTGTAATACGACTCACTATAGGGCGTGCCTTCAACCTCCAA). The *Shox2* probe was extracted from the plasmid donated by John Cobb^86^ by digestion with NcoI. After PCR amplification or plasmid digestion product was purified with Monarch PCR clean-up kit (NEB, #T1030s) and used to produce the DIG-labeled single-stranded RNA probes using the DIG RNA labeling kit (Roche, #11175025910). Probes were then cleaned with the MegaClear Kit (Thermo Fisher, #AM1908).

#### WISH staining protocol

Embryos from the desired stage and genotype were fixed overnight in 4% PFA/PBS. Subsequently, the embryos were washed in PBST (PBS with 0.1% Tween), followed by dehydration in MetOH/PBST solutions of increasing concentrations (30%, 50%, and 70%), with final storage at −20 °C in 100% methanol. For the WISH protocol, on the first day, embryos were bleached in 6% H_2_O_2_/MetOH for 1 h at room temperature (RT), followed by rehydration in reverse methanol/PBST steps, then washed in PBST. Embryos were then treated with 2 µg/ml proteinase K (Promega, V3021)/PBST for 3 min, incubated in 2 mg/ml glycine/PBST, washed again in PBST, followed by three 30 min washes in RIPA buffer (5 M NaCl; 10% NP-40; 10% Deoxycholate; 20% SDS; 500 mM EDTA pH8; 1 M Tris-HCl pH8) and finally refixed for 20 min with a 4% PFA. Following five additional PBST washing steps, embryos were incubated at 68 °C in L1 buffer (50% De-ionized formamide; SSC 5X pH4.5; 1% SDS; 0.1% Tween 20) for 10 min. Next, embryos were incubated for 2 h at 68 °C in hybridization buffer 1 (L1 Buffer, tRNA 100ug/ml, heparin 50 μg/ml), followed by overnight incubation at 68 °C in hybridization buffer 1 containing 150–200 ng/ml of digoxygenin probe, previously denaturalize 10 min at 80 °C in hybridization buffer 1. On the second day, unbound probe removal involved three washes of 30-min at 68 °C with L1 buffer, three of L2 buffer (50% De-ionized formamide; SSC 2X pH4.5; 0.1% Tween 20), and one of 15 min with L3 buffer (SSC 2X pH4.5; 0.1% Tween 20), followed by 40 min incubation at RT to let the buffer cool down. After, embryos were washed three times in TBST (TBS with 1% Tween) and pre-incubated with blocking solution (10% serum/TBST) for 2 h, before overnight incubation at 4 °C in blocking solution containing a 1/5000 dilution of anti-digoxigenin-alkaline phosphatase (Roche, # 11093274910). On the third day, unbound antibody was removed through a series of 30-min washes at RT with TBST, followed by overnight incubation at 4 °C. On the fourth day, staining was initiated by washing at RT with NTMT solution (100 mM NaCl; 100 mM Tris, pH 9.5; 1% Tween; 50 mM MgCl_2_), followed by staining with BM Purple (Sigma #11442074001). *Shox2* expression was assessed by WISH at E12.5 in *Shox2*^*dmCherry*/+^ mouse embryos. *Hoxd13* expression was assessed by WISH at E13.5 in *Shox2*^*Ull*^ and CD1 control mouse embryos. Images were taken using a petri dish with a thin top layer of 1% agarose on a Zeiss Axio Zoom V16 using the Axiocam 506 color camera, the Zen Blue Software v3.6. WISH experiments were also performed in duplicates, with only one replicate imaged. All replication attempts were successful.

### Tissue collection and single-cell dissociation

Forelimb or Hindlimb buds of E10.5, E11.5, E12.5, E13.5, or E14.5 control (*Shox2*^*dmCherry/+;RosaEYFP/+*^*)* or mutant embryos were micro-dissected in 1X DPBS (Gibco, 14190-094) and placed in 1.5 ml tubes. For E10.5, E11.5, E12.5, at least two pairs of limbs were pulled together; for E13.5, only one pair of limbs was dissociated per tube, and for E14.5 embryos, each limb was processed individually. After DPBS removal, each tube containing pairs of limb buds were incubated with 400 μl trypsin-EDTA 0.25% (Thermo Fischer Scientific, 25300062) supplemented with 40 μl of 5% BSA in PBS (Sigma-Aldrich, A7906-100G), during 8–9 min for small embryos (E10.5 and E11.5) or 12–15 min for larger embryos (E12.5, E13.5 or E14.5) at 37 °C in a Thermomixer with a resuspension step after the first 6 min and at the end of the rest of the incubation time. After Trypsin inactivation with one volume of 2.5% BSA, cells were passed through a 40 μm cell strainer, and another volume of 2.5% BSA was added to wash the cell strainer. Cells were spun at 400 × *g* for 5 min at 4 °C and resuspended in 1%BSA in PBS (5 mM Na-Butyrate was added in case the cells were processed to be sorted and later used for downstream ChIP experiments). The single-cell suspension obtained from this process was later used for subsequent flow cytometry experiments or single-cell library preparation. In the latter case, cells were then counted using an automatized cell counter, and a 1% BSA 700 cells/μl suspension was prepared.

### Single-cell RNA-seq library preparation

Chromium Single Cell 3′ GEM, Library & Gel Bead Kit v3 (10X Genomics, #PN-1000075) or v3.1 (10X Genomics, #PN-1000121) was used to prepare single-cell libraries following 10X Genomics manufacturer’s protocol by the iGE3 Genomic Platform. On average, 7000 cells were loaded on the Chromium Chip. In brief, Single-Cell 3’ Gel Beads were combined with the Master Mix containing single cells, and Partitioning Oil onto Chromium Chip B, to generate Gel Beads in Emulsion (GEMs). Full-length cDNA was produced, during bead incubation, from the poly-adenylated mRNA barcoded. Right after, gel beads were dissolved, and cDNA was amplified via PCR. This cDNA was used to construct the library and then sequenced on an Illumina HiSeq 4000 or an Illumina NovaSeq 6000. On average, between 45000-58000 mean reads were obtained.

### Flow cytometry sorting and analysis

FACS was used to isolate cell populations based on the dmCherry and EYFP fluorescent signal by using the BD FACS Aria fusion with a blue laser (488 nm, filter 530/30) for the EYFP signal and with a YG laser (561 nm, filter 610/20) for the dmCherry signal. When sorting of cells was not required, and only recording of cell population proportion was needed, we used the Beckman Coulter Cytoflex analyzer with a blue laser (488 nm, filter 525/40) for the EYFP signal and with a YG laser (561 nm, filter 620/20) for the dmCherry signal. At least 10^5^ cells were recorded for each sample. In both cases, a first FSC/SCC gating was set between 30/40 and 210/240 to exclude debris, followed by dead cells removal using a viability dye (DAPI, AppliChem, #A10010010 for the BDAria or DRAQ7, Invitrogen, D15106 for the Cytoflex). Then, doublets were excluded before establishing the final gating. Flow cytometry analysis from Cytoflex recorded data was performed with the FlowJo™ Software (version 10.9.0) to apply a homogeneous gating to the different experiments, to extract cell proportions, and perform a comparative analysis across the different datasets. A statistical two-sided *t*-test for pair-wise comparison of cell proportion changes was performed using R and ggplot2 (version 3.4.4). At least two replicates were performed for each condition. R code used for statistical test and boxplot representation can be found https://github.com/RROUCO/Scripts_for_Rouco_et_al_2025/.

### RNA extraction

For each experimental condition, total RNA was extracted from frozen pellets of biological replicates, using the RNeasy Micro Kit (Qiagen, 74004). FACS sorting frozen pellets, containing between 3.5 × 10^4^ and 1.5 × 10^5^ cells, were obtained by spinning down the cells after sorting at 1000 × *g* for 5 min at 4 °C, removing the supernatant, and snap freezing at −80 °C. In the case of entire limb samples for bulk RNA-seq experiments or distal and proximal dissected samples for RT-qPCR experiments, samples were dissociated as described above, and 0.5–1 × 10^6^ cells were frozen. At least two replicates were performed for each condition, except for the sample HL_Shox2trac_E105_DECOM_rep1, which, due to technical limitations, could not possible to process a second replicate. Quantification of total RNA was performed with Qubit 2.0 (LifeTechnologies) and the RNA High Sensitivity Assay (Q32852).

### Reverse transcription and qPCR

Isolated RNA from proximal and distal dissected forelimbs of CD1 wildtype and *Shox2*^*Ull*^ mice was reverse transcribed following the SuperScriptII RT (Invitrogen #18064-014) protocol, starting from 200 ng of RNA, using random hexamer primers (Thermo Scientific #S0142), DTT (Invitrogen Y00147), and RNaseOUT (Invitrogen 100000840). *Hoxa13* RT-qPCR primers shown in Supplementary Data 8 were designed using NIH Primer-Blast, while *Hoxd13* and *Rps9* primers were the same as described in ref. ^87^. cDNA amplification was performed using PowerUP™ SYBR Green Master Mix (Applied Biosystems A25742) and run in the QuantStudio 1 RT-PCR System from Applied BioSystems. Averaged CT values were normalized to the *Rps9* house-keeping gene. Values for 2^−ΔCT^ were analyzed using a *t*-test. R code used for statistical test and boxplot representation can be found in https://github.com/RROUCO/Scripts_for_Rouco_et_al_2025/

### RNA-seq library preparation

RNA-seq libraries were prepared by the iGE3 Genomic Platform using the SMART-Seq v4 kit (Clontech, 634893) for the reverse transcription and cDNA amplification, starting from 5 ng of total RNA. For library preparation, 200 pg of cDNA were used with the Nextera XT kit (Illumina, FC-131-1096). Library molarity and quality were assessed with the Qubit and TapeStation using a DNA High Sensitivity chip (Agilent Technologies). Libraries were pooled at 2 nM and loaded for clustering on a Single-read Illumina Flow cell for an average of 35 million reads/library. Reads of 50 bases were generated using the TruSeq SBS chemistry on an Illumina HiSeq 4000 or Illumina NovaSeq 6000 sequencer.

### Cell preparation for ChIP-seq and C-HiC

After FACS sorting, cells were centrifuged at 1500 × *g* for 5 min at 4 °C, and the supernatant was discarded. Cells were resuspended in 10% FCS/PBS and fixed either with 1% PFA (Sigma-Aldrich #252549) for ChIP-seq or with 2% PFA for C-HiC for 10 min rolling. Fixation was halted by adding 1.425 M glycine, followed by centrifugation at 1000 × *g* for 8 min at 4 °C. Then, cells were lysed in cold lysis buffer (10 mM Tris-HCl pH 7.5, 10 mM NaCl, 5 mM MgCl_2_, 1 mM EGTA, with Roche Protease Inhibitor #04693159001) and incubated on ice for 10 min to extract nuclei. Nuclei were then centrifuged at 1000 × *g* for 5 min at 4 °C, washed in cold 1× PBS, and centrifuged again 1000 × *g* for 1 min at 4 °C. PBS was removed and nuclei frozen at −80 °C.

### ChIP-seq immunoprecipitation and library preparation

To prepare ChIP-seq libraries, 0.5 × 10^6^ fixed frozen nuclei were used per sample. One replicate was performed for each sample. Prior to sonication of frozen nuclei, 30 μl of magnetic Protein G beads (Invitrogen 10003D) or Protein A beads (Invitrogen 10001D) were pre-washed in 0.25%BSA/DPBS and resuspended in 1 ml of L3 sonication buffer (10 mM TrisCl pH 8.0, 100 mM NaCl, 1 mM EDTA, 0.5 mM EGTA, 0.1% Na-Deoxycholate, 0.5% N-Laroylsarcosine, filtered and with Roche Protease Inhibitor #04693159001) with 1% Triton. In the case of H3K27Ac antibody (Diagenode Cat# C15410174, RRID:AB_2716835), 2.5 µl was added to Protein G, while in the case of H3K27me3 antibody (Milipore Cat# 07-449, RRID:AB_310624) 3 µl was added to Protein A, and beads were left to rotate at 4 °C for a minimum of 4 h. In parallel, fixed nuclei (an average of 5 × 10^5^ cells) were sonicated 8 pulses (30 s ON / 30 s OFF) to 200–500 bp fragments using a Bioruptor Pico sonicator (Diagenode) in 200 µl of L3 buffer. After sonication 1% Triton was added to the samples, which were centrifuged 10 min at 4 °C. Subsequently, chromatin was combined with the magnetic beads from which unbound antibodies were previously removed, and 1.1 ml of fresh L3 buffer was added. Samples were subjected to overnight rotation at 4 °C. The following day, unbound chromatin was removed through seven washes in RIPA buffer (1% NP-40, 0.7% Na-Deoxycholate, 1 mM EDTA, 50 mM HEPES-KOH, pH 7.55, and 0.5 M LiCl with Roche Protease Inhibitor #04693159001) and one in TE buffer (1 mM EDTA, 10 mM Tris, pH 8). Chromatin was then eluted and subjected to de-crosslinking overnight with the addition of 5 μL Proteinase K (10 mg/mL, Promega, V3021) at 65 °C. This was followed by treatment with RNase A (4 μL, 10 mg/mL) at 37 °C for 30 min, phenol:chloroform:IAA extraction, followed by a chloroform cleaning step, and precipitation (1/10 NaAc, 3 μl of glycogen and 2.5 volumes of EtOH 100%) at −80 °C for 30 min. Chromatin was then washed with 1 ml EtOH 80%. Finally, the chromatin was eluted in 100 μL H_2_O. Na-Butyrate (5 mM) was added to all buffers. Then, libraries were prepared by the iGE3 Genomic Platform. Briefly, ChIP-enriched DNA (<10 ng) was used to prepare libraries with the Illumina TruSeq ChIP kit, following the manufacturer’s guidelines. Libraries were validated on a Tapestation 2200 (Agilent) and a Qubit fluorimeter (Invitrogen – Thermofisher Scientific). Libraries were pooled at 2 nM and loaded for clustering on a Single-read Illumina Flow cell. Reads of 50 bases were generated using the TruSeq SBS chemistry on an Illumina HiSeq 4000 or an Illumina NovaSeq 6000 sequencer.

### C-HiC and library preparation

To prepare C-HiC libraries, 1 × 10^6^ fixed frozen nuclei were used either per FACs-sorted sample or entire dissociated forelimbs. One replicate was performed for each sample. Nuclei were taken up in 520 µl of 1× DpnII buffer (NEB, R0543M) and incubated with 7.5 µl of 20% SDS for 1 h shaking at 600 r.p.m. at 37 °C. Subsequently, 75 µl of 20% Triton X-100 was added and incubated by shaking at 600 r.p.m. at 37 °C for another hour. A 40-µl aliquot was preserved as a control for undigested chromatin (stored at −20 °C). Chromatin digestion was initiated using 600 µl of DpnII buffer and 400 Units of DpnII, shaking at 600 r.p.m. at 37 °C for 6 h; then, 400 Units of DpnII were added, and the samples were incubated overnight, shaking at 600 r.p.m. at 37 °C. The following morning, 200 Units more of DpnII were added, and the samples were incubated 4 h, shaking at 600 r.p.m. at 37 °C. An 80-µl aliquot was extracted to assess digestion efficiency (stored at −20 °C). DpnII restriction enzyme was subsequently inactivated at 65 °C for 25 min. Next, the digested chromatin was diluted and religated in 5.1 ml H2O, 700 µl of 10× ligation buffer (1 M Tris-HCl pH 7.5; 500 mM DTT; 500 mM MgCl2; 100 mM ATP), and 100 Units (Weiss units) T4 DNA ligase (Thermo Fisher Scientific, #EL0013), incubated at 16 °C for 4 h. The ligated samples were further incubated for 30 min at RT. De-crosslinking of samples and test aliquots occurred overnight by adding 30 µl and 5 µl proteinase K (10 mg/ml, Promega, V3021), respectively, and incubating at 65 °C. On the following day, 30 µl or 5 µl of 10 mg/ml RNase was added to the samples and test aliquots, respectively, and incubated for 45 min at 37 °C. Chromatin was then precipitated by adding 1 volume of phenol-chloroform to the samples and test aliquots, vigorously shaking them, followed by centrifugation at 2200 × *g* at RT for 15 min. The upper phase containing the chromatin was transferred to a new tube. Samples were then prepared for precipitation by adding 7 ml of H_2_O, 1 ml of 3 M NaAc pH 5.2, and 35 ml of 100% EtOH and incubated over the weekend at −20 °C. The precipitated chromatin was isolated by centrifugation at 2200 × *g* for 45 min at 4 °C. The chromatin pellet was washed with 70% ethanol and further centrifuged at 2200 × *g* for 15 min at 4 °C. Finally, the 3 C library chromatin pellet was dried at RT and resuspended in 150 μL of 10 mM Tris-HCl, pH 7.5. Quantification of total religated product was performed using the Qubit High Sensitivity DNA Assay (Q32851). To assess the 3C library, 5 μl of the religated sample was loaded on a 1.5% agarose gel along with the undigested and digested aliquots. Then, libraries were prepared by the iGE3 Genomic Platform. Briefly, the 3C library was then sheared using a Covaris sonicator (duty cycle: 10%; intensity: 5; cycles per burst: 200; time: 6 cycles of 60 s each; set mode: frequency sweeping; temperature: 4–7 °C). Adapters were added to the sheared DNA and amplified according to the manufacturer’s instructions for Illumina sequencing (Agilent). Subsequently, the library was hybridized to custom-designed SureSelect beads and indexed for sequencing (50–100 bp paired-end) following the manufacturer’s instructions (Agilent). Libraries were sequenced on an Illumina HiSeq 4000 or Illumina NovaSeq 6000 sequencer. *Shox2* C-HiC SureSelect library was designed using the GOPHER Java desktop application, version 0.5.7^88^, for the genomic interval mm39:chr3:65103500-68603411 covering the *Shox2* locus and adjacent TADs.

### CUT & RUN cell processing and library preparation

Proximal and distal forelimbs from CD1 wildtype and *Shox2*^*Ull*^ E13.5 embryos were micro-dissected (see Fig. 7B) and dissociated following the same procedure described above. After the final centrifugation step, cells were processed following the CUT&RUN protocol with some modifications^89^. Cells were resuspended in 1 ml of cold Wash Buffer (20 mM HEPES-KOH pH 7.5, 150 mM NaCl, 0.5 mM spermidine prepared with EDTA-free Protease Inhibitor Cocktail Roche #04693132001) and centrifuged at 500 × *g* for 2 min at 4 °C, repeating this wash once. Cells were then resuspended in 1 ml of Wash Buffer, counted, and 250,000 cells were isolated for further binding to Concanavalin A-coated beads. For bead mix preparation, 10 µl of Con A-coated BioMag® Plus beads (86057) per sample were washed in 1 ml of Binding Buffer (20 mM HEPES-KOH pH 7.5, 10 mM KCl, 1 mM CaCl₂, 1 mM MnCl₂), followed by a 0.5 ml wash and final resuspension in 10 µl of Binding Buffer. Cells were incubated with 10 µl of bead mix at 4 °C for 10 min with rotation. Bound cells were magnetically separated and resuspended in 100 µl of Antibody Solution (8 µl of 0.5 M EDTA in 2 ml of Dig-Wash Buffer) containing 0.5 µl of HOXD13 antibody (Abcam AB229234). Dig-Wash Buffer was prepared by adding 200 µl of 5% digitonin (Apollo APOBID3301) to 50 ml of Wash Buffer, for a final concentration of 0.02%. Stock of 5% digitonin was prepared by dissolving 50 mg of powder in 1 ml of hot water (95 °C with shaking for 10 min). Once the samples were in the Antibody Solution were transferred into PCR tubes for overnight incubation at 4 °C, rotating in 50 ml tubes. After the incubation, samples were transferred back to a 1.5 ml tubes, magnetically separated, washed in 1 ml and then 500 µl of Dig-Wash Buffer, and resuspended in 100 µl of pA-MN Dig-Wash Buffer (0.5 µl of pA-MNase per 100 µl). pA-MNase was kindly provided by the Duboule lab (produced from Addgene pAG/MNase #123461). Samples were incubated for 1 h on ice with taping every 15 min. Following magnetic separation, cells were washed twice in Dig-Wash Buffer and resuspended in 500 µl of Dig-Wash Buffer. After 5 min incubation on ice, samples were resuspended in 200 µl of Lo-Ca Buffer (4 µl of 100 mM CaCl_2_ in 196 µl of Dig-Wash Buffer) and incubated on ice for 30 min with periodic tapping. Digestion was then stopped by adding 200 µl of 2xSTOP Buffer (340 mM NaCl, 4 mM EGTA, 20 mM EDTA, 0.02% digitonin, 50 µg RNAse A, 50 µg Glycogen) and incubating for 5 min on ice. Fragments were released by a 30 min incubation at 37 °C. Samples were then magnetically separated, and 400 µl of supernatant was transferred to a phase-lock tube. DNA was extracted using phenol:chloroform:IAA, centrifuged for 5 min, followed by aqueous phase precipitation using 1/10 volume NaAc, 3 µl of glycogen, and 2.5 volumes of 100% ethanol at −20 °C for 1 h. After 15 min centrifugation at 4 °C, samples were washed twice with 70% ethanol. Finally, pellets were dried and resuspended in 50 µl of H_2_O. Libraries were prepared by the iGE3 Genomic Platform following starting with 5 ng of CUT&RUN-enriched DNA as starting material and processed with the Illumina TruSeq ChIP Library Preparation Kit and the IDT TruSeq RNA UD Indexes v2. No size selection was performed. Library molarity and quality were assessed with the Qubit (Thermofisher Scientific) and Tapestation (Agilent Technologies - DNA High sensitivity chip). Libraries were sequenced on a NovaSeq 6000 Illumina sequencer for PE 50 reads.

### Custom and regular genomes used for NGS analyses

ChIP-seq, RNA-seq, and scRNA-seq datasets generated in this study were aligned to a customized version of the GRCm39/mm39 assembly^84^ incorporating the dmCherry-P2A-CRE and floxed-SV40pASTOP-EYFP cassettes as artificial chromosomes, which we termed as GRCmm39/ mm39_dsmCherry_P2A_CRE_EYFP genome, and it is available on https://zenodo.org/records/14865689. NGS datasets downloaded from GEO and reanalyzed in this study^37,57^ were aligned to the regular GRCm39/mm39 assembly^84^. For annotation, GTF files sourced from ENSEMBL GRCm39 release 104^84^ were used, with a filtering process applied to exclude read-through/overlapping transcripts. Only transcripts annotated as “protein-coding” for their respective genes were retained, while those flagged as “retained_intron,” “nonsense-mediated decay,” etc., were discarded. This filtration aimed to retain only unambiguous exons, mitigating potential quantitative biases during data analysis conducted using STAR/Cufflinks^90^. This GTF file can also be found on https://zenodo.org/records/14865689.

### Single-cell analysis

#### Processing of sequenced reads

Demultiplexing, alignment, filtering of barcodes, and UMI counting of two replicates for each stage of interest E10.5, E11.5 and E12.5, except from E13.5 that only one replicate was produced, were executed using the 10× Genomics Cell Ranger software (version 6.1.2) in accordance with the manufacturer’s guidelines, default settings and custom genome GRCmm39/ mm39_dsmCherry_P2A_CRE_EYFP built following using the cellranger mkref pipeline. Cell Ranger output files for each dataset were further processed using the velocyto run10x command from the velocyto.py tool (version 0.17.17)^52^ in Python (version 3.9.12) with our custom genome GTF, available on https://zenodo.org/records/14865689, and the UCSC genome browser repeat masker.gtf file to mask expressed repetitive elements to generate a loom file for each sample. Each resulting loom matrix, comprising spliced/unspliced/ambiguous reads, was individually imported into R (version 4.1.2) using the Read Velocity function from the Seurat Wrappers package (version 0.3.0). Simultaneously, feature-filtered output matrices obtained from Cell Ranger were loaded into R separately through the Read10X function of the Seurat package (version 4.2.1)^91^. Subsequently, the spliced, unspliced, ambiguous, and RNA feature data were combined into a single matrix for each dataset. Following this, each matrix was transformed into a Seurat object using the Seurat package. Consequently, for each sample, a single Seurat object was obtained, encompassing four assays. Three of these assays (spliced, unspliced, and ambiguous) were used for downstream RNA-velocity estimations, while the RNA feature assay was employed for subsequent gene expression analysis among the samples, as detailed below.

#### Quality control and filtering

Quality control and pre-processing of each Seurat object for our samples were conducted based on the following criteria. Cells expressing fewer than 200 genes or exhibiting more than 7500 features were excluded from the analysis. Additionally, we calculated the proportion of reads mapping to the mitochondrial genome, filtering out cells with a mitochondrial content exceeding 5%, as elevated levels of mitochondrial mRNA have been linked to cell death. Conversely, cells with a mitochondrial content lower than 0.5% were also excluded, as our observations suggest that these cells likely originate from blood cells, possibly due to the dissection protocol. After this step, we decided to continue the analysis with only one replicate per stage. The replicate with the best quality control for each stage was selected.

#### Individual dataset normalization, scaling, and dimensional reduction

After filtering, each dataset was individually normalized using the default parameters provided by Seurat for the LogNormalize method and applying it to the RNA features assay. Subsequently, we calculated the most variable features, excluding the *CRE*, *EYFP*, and *dmCherry* artificial genes added to our custom genome, from the list of variable genes to avoid that they drive the principal component analysis (PCA). Then, scaling was performed via linear transformation, and the scaled data were then employed for PCA, utilizing the default 50 principal components. Additionally, non-linear dimensional reduction was conducted using Uniform Manifold Approximation Projection (UMAP)^92^, with 1:50 dimensions utilized as input.

#### Cell doublet identification and features annotation

Pre-processed and normalized datasets were individually examined to detect putative doublet cells. Doublets identified in each dataset were subsequently excluded using the DoubletFinder R package (version 2.0.3)^93^. The doublet rate (nExp parameter) utilized was estimated based on the number of cells captured, and the pK parameter was estimated following the strategy defined in the package, resulting in the following values: *Shox2*^*trac*^ Hindlimb E10.5, nExp = 89, pK = 0.3; E11.5 nExp = 98, pK = 0.16; E12.5 nExp = 71, pK = 0.25; E13.5 nExp = 88, pK = 0.1. After removing doublets, counts for *CRE*, *dmCherry*, *Shox2*, and *EYFP* per cell were estimated. Given the low counts for *dmCherry* (likely a limitation due to the 10X single-cell technique, where transcripts are only sequenced from the 3′ polyA end, which does not allow for adequately cover the *dmCherry* sequence), we proceeded with further cell classification using only *Shox2*, *EYFP*, and *CRE* counts. Moreover, from that point on, we referred to *CRE* counts as *dmCherry-P2A-CRE*, since we assumed that it could be used as a proxy for both *dmCherry* and *CRE* genes. Of note, due to the shared SV40polyA tail between *dmCherry-P2A-CRE and EYFP*, many reads were ambiguous, leading to a limited number of reads assignable to *dmCherry-P2A-CRE*, which we anticipated to be lower in expression than *EYFP* (as constitutively expressed from the *ROSA26* promoter). Cells were then classified as positive for each of these genes if they had at least one count, and negative otherwise. Cells positive for *Shox2*, negative for *EYFP*, and either positive or negative for *dmCherry-P2A-CRE* were classified as *initiating*. Those positive for both *Shox2* and *EYFP*, regardless of *dmCherry-P2A-CRE* status, were classified as *maintaining*. Cells negative for both *Shox2* and *dmCherry-P2A-CRE* but positive for *EYFP* were considered *decommissioned*. Cells negative for all three genes were marked as inactive, and any remaining combinations of gene expression fell under the class “other.” A new metadata column containing the classification of the cells was then created.

#### Merge of all datasets and normalization

All datasets were then merged into a single Seurat object without undergoing integration, allowing for ensemble downstream analysis of the four datasets. Subsequently, no batch effect was observed in this merged dataset. A new column of the metadata was created at this step to label samples based on the stage, to keep this information for downstream analysis. Afterward, we applied the SCTransform normalization protocol^94^ to our newly merged Seurat object, utilizing default parameters, over the spliced assay.

#### Cell-cycle scoring and cell-cycle and stage regression

Since we observed, during individual dataset analysis, that a portion of the variance was attributable to cell-cycle genes, we assigned cell-cycle score using the CellCycleScoring function implemented in Seurat. As we also observed that sample variance by a stage effect, we regress out the cell-cycle heterogeneity and stage variability by applying the SCTransform normalization method to our merged object, using the spliced assay as the source, and incorporating the calculated cell-cycle scores (S.Score and G2M.Scores) and the stage metadata information as variables to regress, in addition to the default settings. Subsequently, we excluded *dmCherry*, *CRE*, and *EYFP*, if they were present from the variable genes to avoid that they drive the PCA.

#### Clustering

Following the regression step, cells were clustered using the standard steps of the SCTransform Seurat workflow. Briefly, PCA (npcs = 50), UMAP (dims = 1:50, n.neighbors = 50), and nearest neighbors were calculated. Clusters were identified using the Seurat FindClusters function with default parameters and a resolution of 0.7, resulting in the definition of 21 clusters. Cluster identity was determined by assessing the expression difference of each gene between each cluster and the rest of the clusters using the FindMarkers function. Clusters presenting similar features profiles were combined, reducing the final number of clusters identified to 6 clusters (Supplementary Fig. 2A). The mesenchyme (comprising 13 out of the 21 clusters), epithelium (consisting of 3 out of 21), muscle (comprising 2 out 21), and endothelium, immune Cells, and blood Cells clusters each represented by only 1 cluster. The presence of expected identity markers in the new clustering was confirmed by running the FindMarkers function with default parameters and using grouping.var = “stage” and only.pos = TRUE.

#### Subsetting and re-clustering

Given the focus of this study on populations expressing *Shox2*, we subsetted and re-clustered the mesenchyme cluster. To do, after applying the subset function to the “Mesenchyme” UMAP embedding, was computed with the following parameters: dims = c(1:10), n.neighbors = 30 L, min.dist = 0.5, metric = “euclidean,” spread = 1, while keeping all other parameters at their default values. Subsequently, the cluster resolution after finding neighbors was set at 1.1 to reveal subpopulations. We observed 18 mesenchyme subpopulations, each named based on their identity genes. Identity markers were identified using the FindMarkers function on the RNA assay, with grouping.var = “stage,” only.pos = TRUE, logfc.threshold = 0.3, min.diff.pct = 0.1, and all other parameters set to default values. Clusters presenting similar feature profiles were combined, reducing the final number of clusters identified to 15 clusters (Fig. 2B), late proximal progenitors (comprised 3 out of the 18 clusters), and irregular connective tissue (contained 2 out of the 18). The other clusters remained represented by 1 cluster. Final identity markers for the new clustering were assessed by running the FindMarkers function on the RNA assay only.pos = TRUE, logfc.threshold = 0.5, pseudocount.use = 0, min.diff.pct = 0.1, and all other parameters set to default values (Supplementary Data 2).

#### RNA-velocity analysis

For the RNA-velocity analysis, we used the unspliced (immature) and spliced (mature) abundances calculated for each replicate of our datasets, as described earlier (see “Methods,” “Single-cell analysis,” “Processing of sequenced reads”). We then performed RNA-velocity analysis on all combined datasets by exporting the Seurat object as h5Seurat files using the SeuratDisk package (version 0.0.0.90) and using it as input in Scvelo (version 0.2.5)^53^ in Python (version 3.9.16). Then the standard protocol described in scVelo was followed, with the exception of using npcs = 10 and n.neighbors = 30, to match the parameters used for UMAP embedding in Seurat. *Shox2* velocity was computed by running the velocyto package for R (version 0.6)^52^ with default parameters on the Seurat Object to generate an embedding file from which *Shox2* was only plotted using ggplot2 (version 3.4.4).

#### Graphical plots

FeaturePlot and VlnPlot were generated from the RNA assay of the Seurat objects. FeaturePlot and Dimplot were produced using default Seurat parameters. Density UMAP plots were produced using the Nebulosa v1.4.0 package^95^. Cell proportions were calculated using the prop.table tool from the base R package (version 4.1.2), followed by plotting using ggplot2 (version 3.4.4). The scripts used to perform this analysis can be found at https://github.com/RROUCO/Scripts_for_Rouco_et_al_2025/.

### RNA-seq analysis

FASTQ files from FACS-sorted cells or entire limbs generated in this study were processed using CutAdapt v1.18 to trim NextSeq adapter sequences and low-quality bases^96^, employing the adapter sequence -a CTGTCTCTTATACACATCTCCGAGCCCACGAGAC with a quality cutoff of 30 (-q30) and a minimum length requirement of 15 bases (-m15). In the case of the samples from GEO datasets that we wanted to reanalyze^37^ CutAdapt was used to trim TruSeq adapter sequences and low-quality bases, using the following parameters -a GATCGGAAGAGCACACGTCTGAACTCCAGTCAC, -q30, and -m15. Unstranded reads were then mapped on the customized genome GRCm39/ mm39_dsmCherry_P2A_CRE_EYFP, in the case of the datasets produced in this study, or to the GRCm39/mm39, in the case of the reanalyzed dataset. The STAR version 2.7.2b^97^ was then used together with the filtered GTF file generated for this study (see “Custom and regular genomes for NGS analyses” in this “Methods” section) for accurate gene quantification using tailored settings (--outSAMstrandField intronMotif --sjdbOverhang “99” --sjdbGTFfile $gtfFile --quantMode GeneCounts --outFilterType BySJout --outFilterMultimapNmax 20 --outFilterMismatchNmax 999 --outFilterMismatchNoverReadLmax 0.04 --alignIntronMin 20 --alignIntronMax 1000000 --alignMatesGapMax 1000000 --alignSJoverhangMin 8 --alignSJDBoverhangMin 1). FPKM values were then determined by Cufflinks version 2.2.1^98^ using the filtered GTF file generated for this study (see “Custom genome for NGS analyses” in this “Methods” section) and tailored settings (--max-bundle-length 10000000 --max-bundle-frags 100000000 -- multi-read-correct --library-type “fr-firststrand” --no-effective-length-correction -M MTmouse.gtf). Then, Normalized FPKM were computed by determining coefficients extrapolated from a set of 1000 house-keeping genes stably expressed across the series of compared RNA-seq datasets^99^. Differential expression analysis was performed using DEseq2^100^ R package (version 1.34.0) with the Wald test for comparisons across samples and multiple test correction using the FDR/Benjamini-Hochberg test. The scripts used to perform this analysis can be found at https://github.com/RROUCO/Scripts_for_Rouco_et_al_2025/.

### ChIP-seq analysis

#### ChIP-seq reads processing

Reads from ChIP-seq sequencing, either from datasets generated for this study or from GEO datasets that we wanted to reanalyze^37,57^ were processed first using CutAdapt version 1.18^96^ to trim TruSeq or NextSeq adapter sequences and low-quality bases, specifying the adapter sequence with -a GATCGGAAGAGCACACGTCTGAACTCCAGTCAC (for TruSeq) or -a CTGTCTCTTATACACATCTCCGAGCCCACGAGAC (for NextSeq), a quality threshold of 30 with -q30, and a minimum length of 15 bases with -m15. Then reads were mapped to our GRCm39/mm39_dsmCherry_P2A_CRE_EYFP customized mouse genome, in the case of the datasets generated for this study, or to the GRCm39/mm39 in the case of the reanalyzed datasets, using Bowtie2 version 2.3.5.1^101^ with its default settings. Subsequently, only reads with a mapping quality score (MAPQ) of 30 or higher were retained, as filtered with SAMtools view version 1.10 ^102^. For coverage and peak analysis, reads were extended by 200 base pairs and processed using MACS2 version 2.2.7.1^103^ with the parameters --broad --nolambda --broad-cutoff 0.05 --nomodel --gsize mm --extsize 200 -B 2 for broad peak calling, in the case of H3K27ac and H3K27me3 ChIP-seq data produced from the FACS-sorted cells for this study. For reanalyzed datasets coverage and peak analysis, reads were extended by 200 base pairs and processed using MACS2 version 2.2.7.1^103^ with the parameters --call-summits --nomodel --extsize 200 -B 2 for narrow peak calling. The coverage normalization was performed by MACS2, adjusting for the total millions of tags used in the analysis. The scripts used to perform this analysis can be found at https://github.com/RROUCO/Scripts_for_Rouco_et_al_2025/.

#### ChIP-seq reads visualization

For the reprocessed HOXA13 and HOXD13 ChIP-seq^57^, when a peak with a score >100 (MACS2) overlaps one of our enhancers, we considered the region bound.

#### Early, common, and late putative enhancers classification on entire forelimb datasets

RNA-seq FASTQ files from two replicates of entire forelimbs at E10.5 and E13.5^37^ were reanalyzed following the RNA-seq pipeline previously described (see “RNA-seq analysis” in this “Methods” section). Genes related to limb development were selected for downstream analysis. The average of normalized FPKM values was calculated and used to compute the ratio between E10.5 and E13.5 datasets. Then, since we were interested in genes having stable expression between E10.5 and E13.5, they were filtered to keep those with FPKM values greater than 5, at both stages, and we excluded genes having a fold change larger than 3 between the two stages. By applying this filtering, 90 genes were selected (Supplementary Data 1). To analyze putative enhancers, ChIP-seq H3K27Ac datasets of entire forelimbs at E10.5 and E13.5^37^ were first reanalyzed following the ChIP-seq pipeline previously described in this “Methods” section. H3K27ac MACS2 narrowpeaks were then restricted within the interaction domain defined by promoter Capture-C^37^ of the 90 filtered genes, using bedtools (version v2.30.0) intersect function^104^. Then, H3K27Ac peaks around gene promoters were excluded by filtering against a −2 kb/500 bp window centered at the transcription start site of coding genes using again bedtools intersect. At the *Shox2* locus, the peak called at the alternative *Veph1* promoter was manually excluded. H3K27Ac peaks were then classified as putative common enhancers when present in both E10.5 and E13.5 using bedtools intersect. H3K27Ac peaks present only in the E10.5 dataset were classified as putative early enhancers, while H3K27Ac peaks present only in the E13.5 dataset were classified as putative late enhancers. Putative enhancers were then assigned to the gene interaction domain (Supplementary Data 1). In those cases, where putative enhancers were within the overlapping region of two domains putative enhancers were assigned to the two loci. H3K27Ac peaks classified in the three categories were then processed to calculate scores using the compareMatrix function from bedtools. This matrix was then used to produce a heatmap (tornado plot) of the H3K27Ac peaks per category using the plotHeatmap function from bedtools.

### Early, common, and late putative enhancers classification on FACS-sorted, maintaining forelimb datasets

MACS2 BroadPeak files from FACS-sorted, maintaining cells from forelimbs at E10.5, E11.5, E12.5, and E13.5, were used to build bed files. These files were used to merge peaks within 600 bp of each other using bedtools (version v2.30.0)^104^. Then, bedops (version 2.4.41)^105^ --merge operation was used to flatten all disjoint, overlapping, and adjoining element regions into contiguous, disjoint regions peaks among the four different stages. Subsequently, peaks were extended by ±300 bp, using bedtools’ slope function. Since we wanted only to explore the putative enhancers of the *Shox2* locus using bedtools intersect, we selected only the region with the following coordinates: mm39 chr3:66,190,000-67,290,000. In that way, we created a list of peaks of interest. Peaks falling on gene promoters were manually excluded. Then, as we wanted to establish our new early, late, and common enhancer classification, taking into consideration the scores assigned to each peak for each stage analyzed, we used deeptools^106^ multiBigwigSummary function to compute the average scores for each peak in our curated list at each stage. Subsequently, peaks with a coverage lower than 0.3 and peaks smaller than 600 bp were excluded. Finally, we analyze the slope of H3K27ac coverage across the four stages, and we classified enhancers as early (<−0.06), common (>−0.06, <0.06), or late (>0.06). The scripts used to perform this analysis can be found at https://github.com/RROUCO/Scripts_for_Rouco_et_al_2025/.

### C-HiC analysis

The pre-processing and alignment of paired-end sequencing data, along with the filtering of mapped di-tags, were conducted using the HiCUP pipeline (version 0.6.1)^107^ using default parameters for the configuration file and adding Nofill: 1 parameter. Bowtie2 (version 2.3.4.2)^101^ was used by the pipeline for mapping. Subsequently, filtered di-tags were processed with Juicer Tools (v1.9.9)^108^ to generate binned contact maps (5 kb and 10 kb) from valid and unique read pairs with MAPQ ≥ 30 and normalized maps using Knights and Ruiz matrix balancing^109^. For binning and normalization, only the genomic region mm39:chr3:65103500-68603411 covering the *Shox2* locus and adjacent TADs was considered. Subtraction maps were produced from the KR normalized maps and scaled together across their subdiagonals. C-HiC maps of count values, as well as subtraction maps, were visualized as heatmaps in which values above the 99th percentile were truncated for visualization purposes. The scripts used to perform this analysis can be found at https://github.com/RROUCO/Scripts_for_Rouco_et_al_2025/.

### Virtual capture-HiC analysis

Virtual capture-HiC profiles were generated from the hicup.bam files obtained during C-HiC analysis prior to Juicer normalization. A 5 kb viewpoint for the *Shox2* promoter was defined (mm39:chr3:66885043-66890041) to be used for contact analysis over the captured region (mm39:chr3:65103500-68603411). A contact pair was considered when one interaction fragment was in the viewpoint, and its pair mate was outside of it. Reads were counted per restriction fragment, then binned to a regular 1-kb grid. If a fragment spanned more than one bin, the count value was distributed proportionally to the overlaps. Profiles were smoothed by averaging over a sliding window of five bins. Finally, coverage normalization was performed by dividing the profiles by the sum of counts in the enriched captured region on chr3 and multiplying by 10^3^.

### CUT & RUN analysis

Paired FASTQ files of each sample were processed to subsample 78 M read pairs using the seqtk toolkit with a random seed of 100. Reads from these subsampled FASTQ files were then processed first using CutAdapt version 4.9 to trim TruSeq adapter sequences using the option -a GATCGGAAGAGCACACGTCTGAACTCCAGTCAC and to trim low-quality ends using a threshold of 30 with option -q 30, and a minimum length of 15 bases with option -m 15^96^. Then reads were mapped to our GRCm39/mm39_dsmCherry_P2A_CRE_EYFP customized mouse genome using Bowtie2 version 2.5.4 with the following options --very-sensitive --no-unal --no-mixed --no-discordant --dovetail -X 1000 ^101^. Then reads with a mapping quality score (MAPQ) below 30 were removed, with SAMtools view version 1.21 (-q 30)^102^. Then, PCR duplicates were removed with the MarkDuplicates tool from Picard version 2.27.4 before the BAM conversion to BED using bamtobed bedtools version 2.31.1^104^. MACS2 version 2.2.9.1 was used to process the output BED file and obtain the coverage using the options --nomodel --keep-dup all --shift -100 --extsize 200 --call-summits -B 2^103^. The MACS2 output file was then normalized by a million reads. The scripts used to perform this analysis can be found at https://github.com/RROUCO/Scripts_for_Rouco_et_al_2025/.

### ATAC-seq datasets

Bigwig files from ATAC-seq proximal (GSM5828324, GSM5828325) and distal (GSM5828320, GSM5828321) forelimb datasets shown in Supplementary Fig. 12 were directly downloaded for visualization without further processing.

### Reporting summary

Further information on research design is available in the Nature Portfolio Reporting Summary linked to this article.

## Supplementary information

Supplementary Information

Description of Additional Supplementary Files

Supplementary Data 1

Supplementary Data 2

Supplementary Data 3

Supplementary Data 4

Supplementary Data 5

Supplementary Data 6

Supplementary Data 7

Supplementary Data 8

Reporting Summary

Peer Review file

## Source data

Source data

## Data Availability

The sequencing data generated in this study are available in the GEO repository under the accession number GSE262006: [https://www.ncbi.nlm.nih.gov/geo/query/acc.cgi?acc=GSE262006]. The entire forelimb Capture-C, RNA-seq, and ChIP-seq at E10.5 and E13.5 datasets were obtained from the GEO accession number GSE84795. The proximal and distal ATAC-seq E12.5 datasets were obtained from the GEO accession number GSE194114. HOXD13 E12.5 Distal Forelimb ChIP-seq datasets were obtained from GEO accession number GSE81358. RNA-seq replicates 1 and 2 of entire forelimb E12.5 embryos with a G4 control background were taken from previous lab publications, accession numbers GSM8093970 and GSM8093971. Source data are provided with this paper.

## Code availability

scRNA-seq, FACS analysis, RT-qPCRs, RNA-seq, ChIP-seq, C-HiC, and CUT & RUN processing scripts^110^ are available on GitHub https://github.com/RROUCO/Scripts_for_Rouco_et_al_2025/ and citable via the following 10.5281/zenodo.17143322. GRCm39/mm39_eGFP-SV40pA custom genome and the filtered GTF file are available on Zenodo: https://zenodo.org/records/14865689.
