# Supplementary material for: Temporal constraints on enhancer usage shape the regulation of limb gene transcription
Source: Nat Commun. 2026 Jan 12;17:5. doi: 10.1038/s41467-025-66055-6 (PMC12795824; doi:10.1038/s41467-025-66055-6)
Supplement: Supplementary file 1 — Supplementary Information [file 41467_2025_66055_MOESM1_ESM.pdf]

Supplementary Figures to :

Temporal constraints on enhancer usage shape the regulation of limb gene transcription

Raquel Rouco[1,2], Antonella Rauseo[1,2], Fabrice Darbellay[1,2], Guillaume Sapin[1,2], Olimpia Bompadre[1,2], Lucille Lopez-Delisle [1,2], and Guillaume Andrey[1,2]

[1]Department of Genetic Medicine and Development, Faculty of Medicine, University of Geneva, Geneva, Switzerland
[2]Institute of Genetics and Genomics in Geneva (iGE3), University of Geneva, Geneva, Switzerland

Correspondence: guillaume.andrey@unige.ch

Supplementary Figure 1

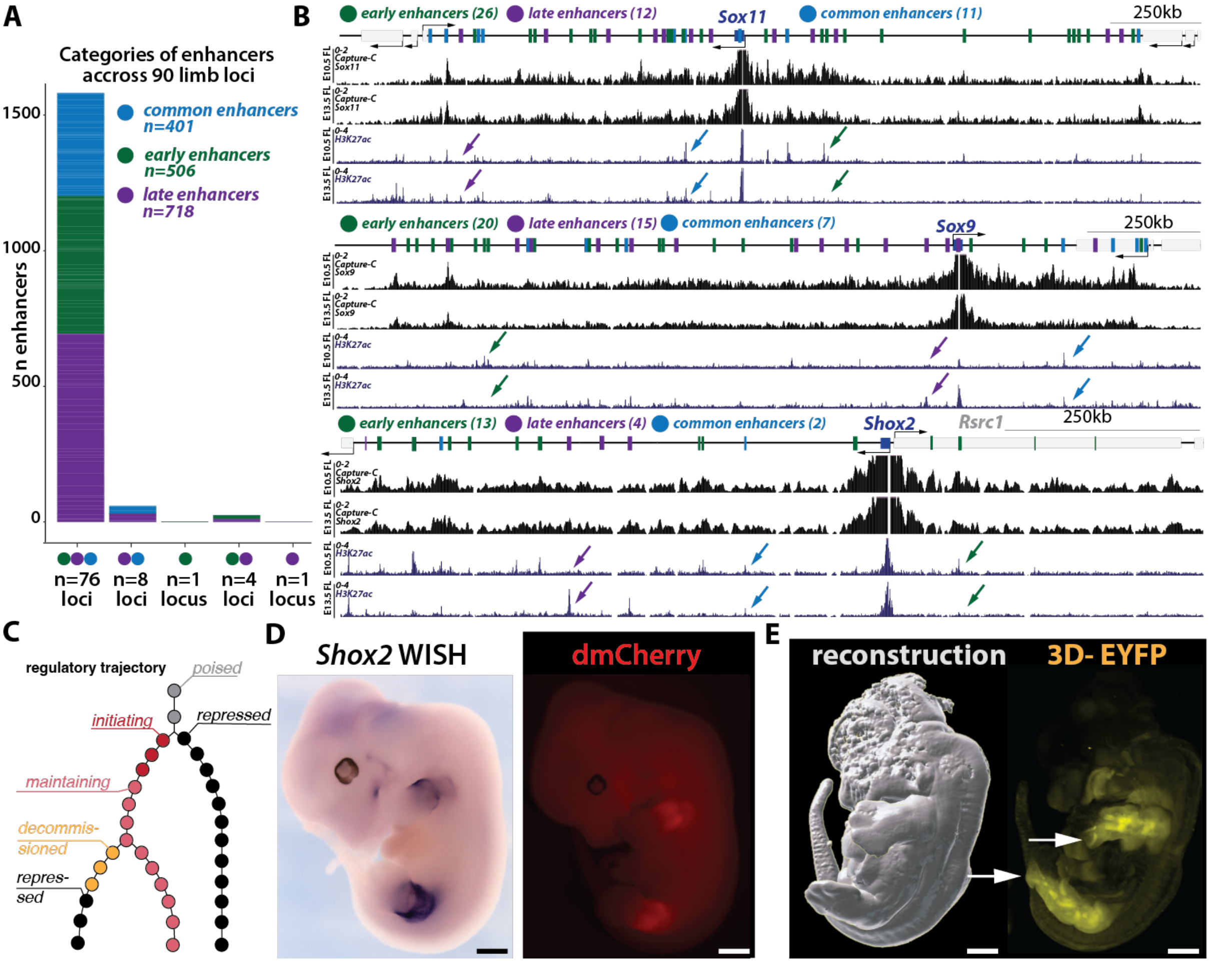

Supplementary Figure 1. A. Distribution of 90 limb-related loci based on the presence of putative early (green), common (light blue), and late (purple) enhancers defined by H3K27ac enrichment on forelimbs at E10.5 and E13.5 within their contact domain. Source data are provided in the Source Data file and in Supplementary Data file 1. **B**. Capture-C interaction profiles (from promoters) and H3K27Ac ChIP profile for examples of developmental loci: *Sox11*, *Sox9* and *Shox2* with early (green balls), common (light blue balls), and late (purple balls) putative enhancer regions based on[1]. Light grey box represents other genes. For each locus, arrows pinpoint a representative example of one early (green), one common (blue) and one late (purple) putative enhancers. **C**. A hypothetical regulatory trajectory begins from a poised state (grey) either towards an inactive state, therefore to locus repression (black) or towards and active state thereby to gene transcriptional initiation (dark red), followed by either transcriptional maintenance (light red) or decommissioning (orange). Eventually, repression (black) can shut down the locus. **D**. *Shox2* whole-mount RNA in situ hybridization (WISH) and dmCherry fluorescence in a *Shox2*$^{dmCherry/+}$ E12.5 embryo (scale bars: 1mm). In total, 2 wildtype and 2 *Shox2*$^{dmCherry/+}$ embryos were used. **E**. Light sheet microscopy reconstruction of EYFP signal in a *Shox2*^{trac} (*Shox2*$^{dmCherry/+}$; *Rosa26*$^{loxEYFP/+}$) E12.5 embryo (scale bars: 1mm). Note the EYFP signal in digit condensations (white arrows).

Supplementary Figure 2

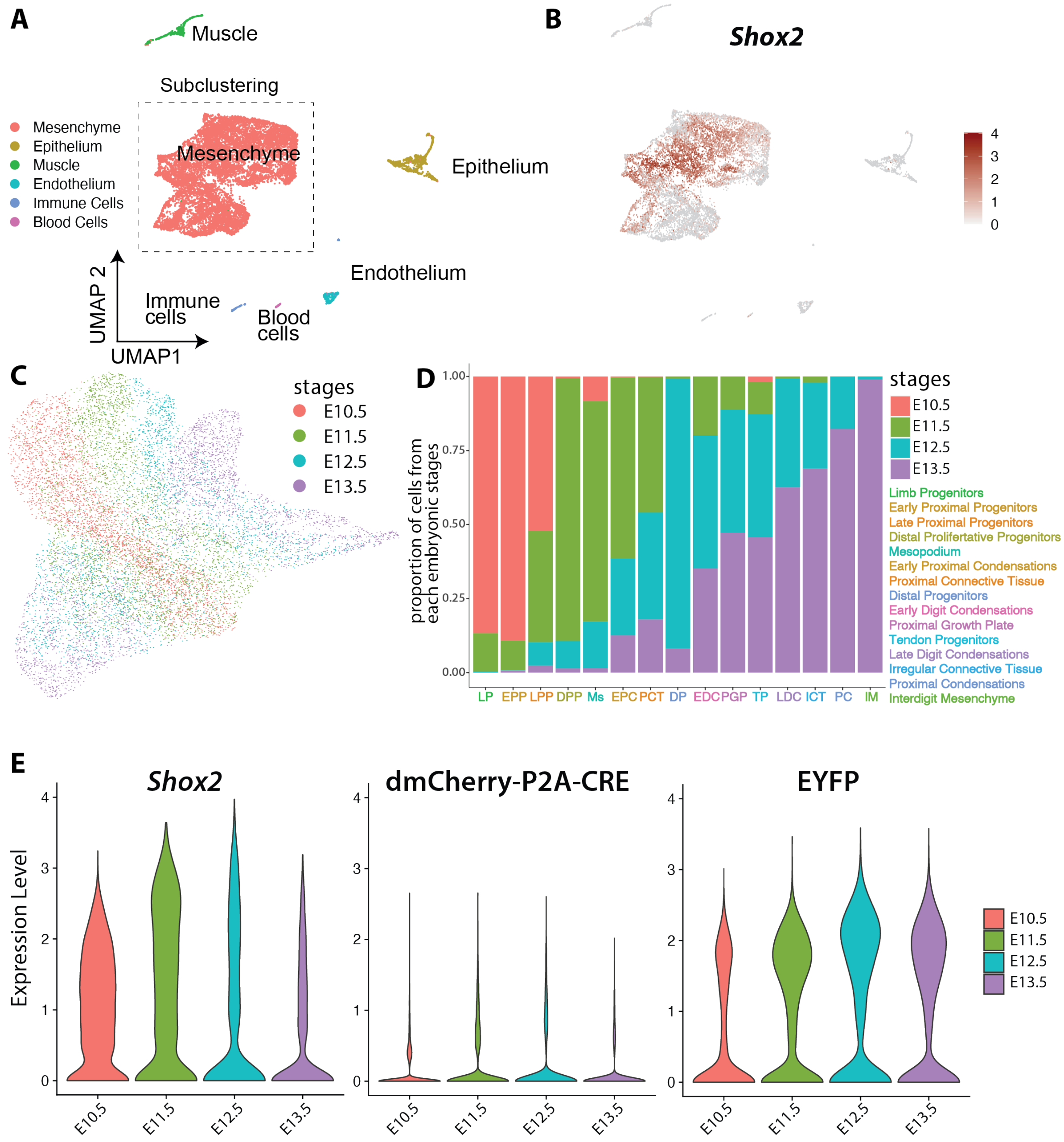

Supplementary Figure 2: A. UMAP clustering of *Shox2*^{trac} E10.5, E11.5, E12.5 and E13.5 hindlimb cells shows one mesenchymal cluster containing most cells as well as five non-mesenchyme satellite clusters. **B**. Expression of *Shox2* across all limb cell types. **C**. UMAP representation of mesenchyme split by development stages. **D**. Proportion of cell split by developmental stage in each mesenchymal cluster, ordered from early to late development. **E**. Violin plot of *Shox2*, dmCherry-P2A-CRE expression across stages and mesenchyme. Source data are provided in the Source Data file.

Supplementary Figure 3

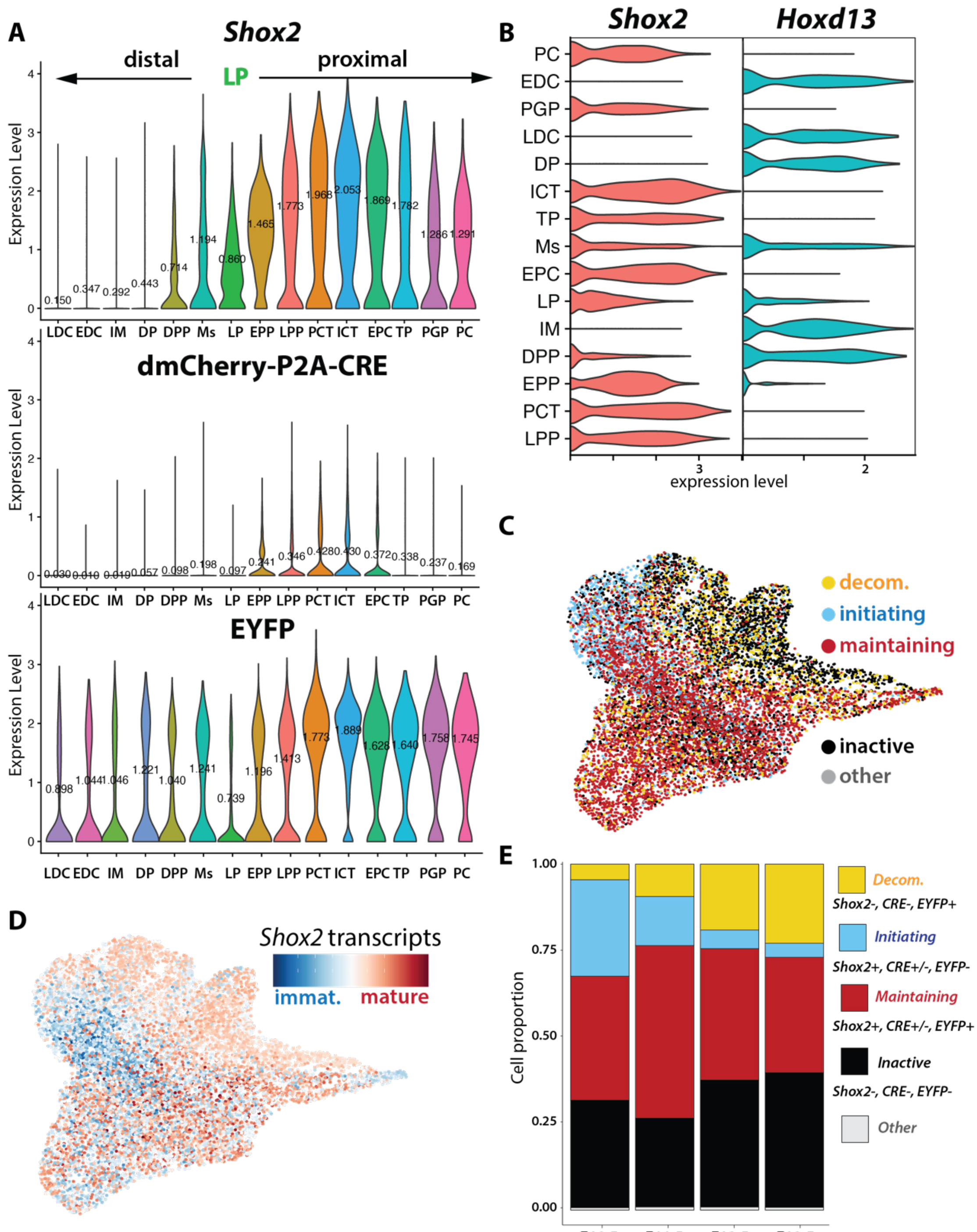

Supplementary Figure 3: **A**. Expression of *Shox2*, dmCherry-P2A-CRE and EYFP in each mesenchymal cluster, ordered according to their distal and proximal identity and to developmental time. Numbers represents average expression levels. **B**. Expression of *Shox2* and *Hoxd13* in each mesenchymal cluster. Note the mutual exclusion of both transcripts except for LP and Ms, and to a smaller extent for DPP. **C**. UMAP representing different *Shox2* cell phases: inactive (black), initiation (light blue), maintaining (red), decommissioned (yellow) and other (grey). **D**. Visualization of *Shox2* RNA-velocity: light blue signifies a higher fraction of immature transcripts (immat.), whereas red indicates a higher fraction of mature transcripts. **E**. Distribution of each Shox2 transcriptional phases: initiation (*Shox2*+, dmCherry-P2A-CRE +/-, EYFP-, light blue), maintaining (*Shox2*+, dmCherry-P2A-CRE +/-, EYFP+, red), decommissioning (*Shox2*-, dmCherry-P2A-CRE-, EYFP+, yellow), inactive (*Shox2*-, dmCherry-P2A-CRE-, EYFP-, black), or other cells (when where not included in any of the previously mentioned class) across developmental stages in all cells including non-mesenchyme clusters. Source data are provided in the Source Data file.

Supplementary Figure 4

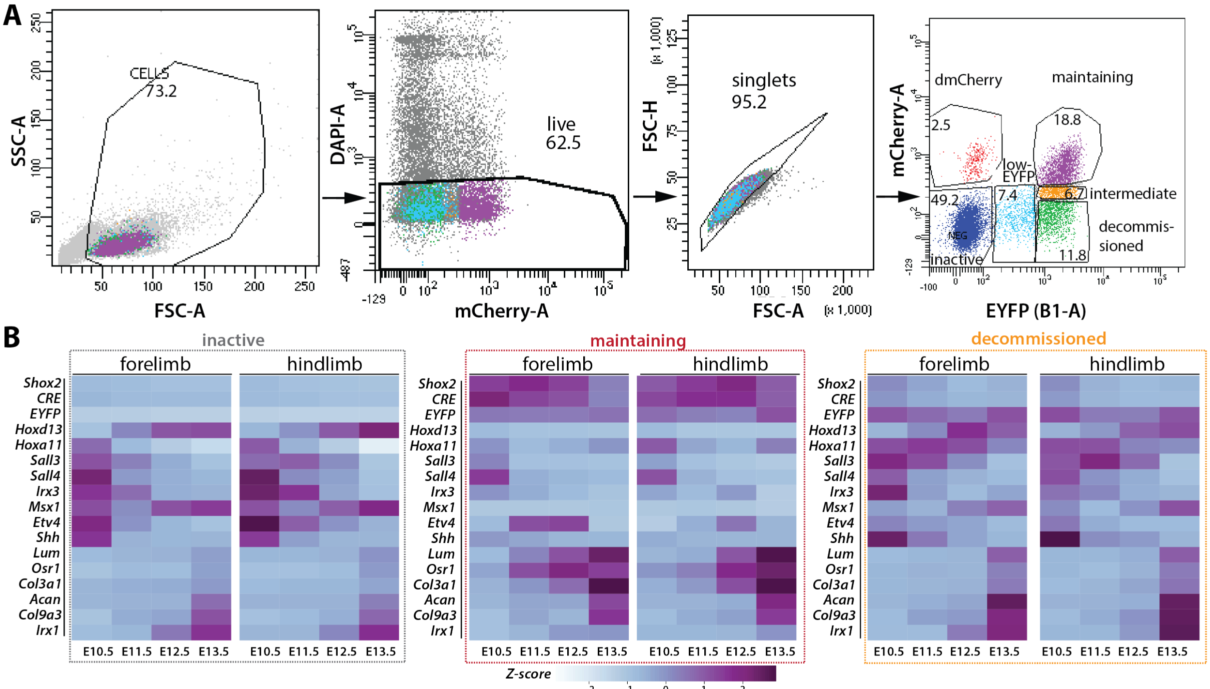

Supplementary Figure 4: A. Illustration of the gating strategy used to sort cell for biochemical analyses (here represented by one replicate of *Shox2^{trac}* E10.5 forelimbs). **B**. Heatmaps of gene expression analysis of marker genes in inactive, maintaining and decommissioned sorted cells throughout fore- and hindlimb development. Z-score scale derived from normalized FPKMs provides a normalized measure by rows of gene expression levels enabling comparison across samples. Source data are provided in the Source Data file and in Supplementary Data file 4.

Supplementary Figure 5

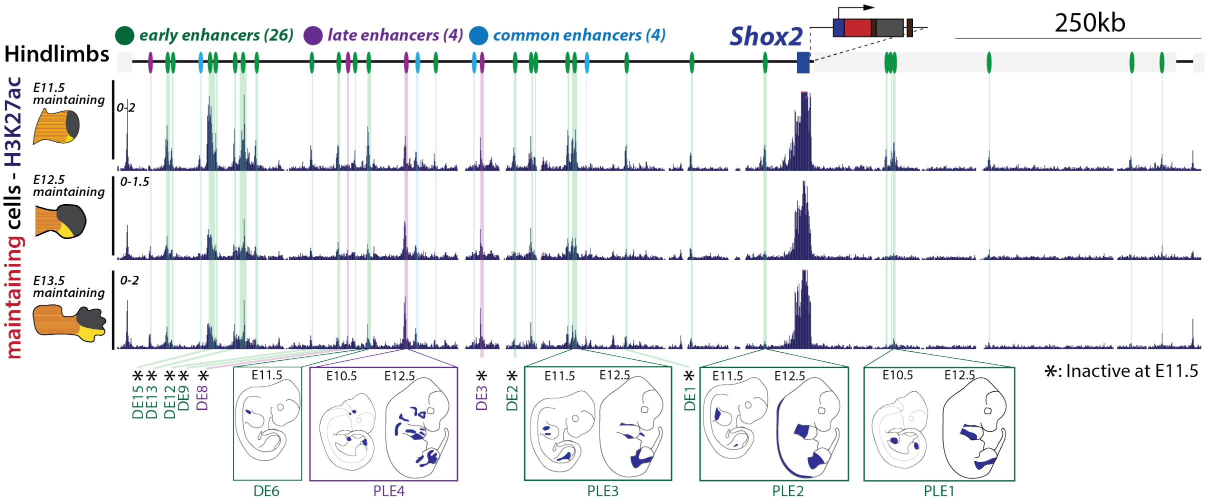

Supplementary Figure 5: H3K27ac ChIP-seq profiles of FACS sorted maintaining (dmCherry+/EYFP+) cells across E11.5, E12.5, and E13.5 hindlimbs (mm39: chr3:66,190,000-67,290,000). Putative enhancers are delineated by color-coded lines: green for early, light blue for common, and purple for late enhancers, as detailed in Supplementary Data S4. Bottom part shows a schematic representation of the pattern displayed by enhancers previously validated through *in vivo* LacZ reporter assays[2,3]. A complete list of the putative enhancers identified can be found in the Supplementary Data file 5.

Supplementary Figure 6

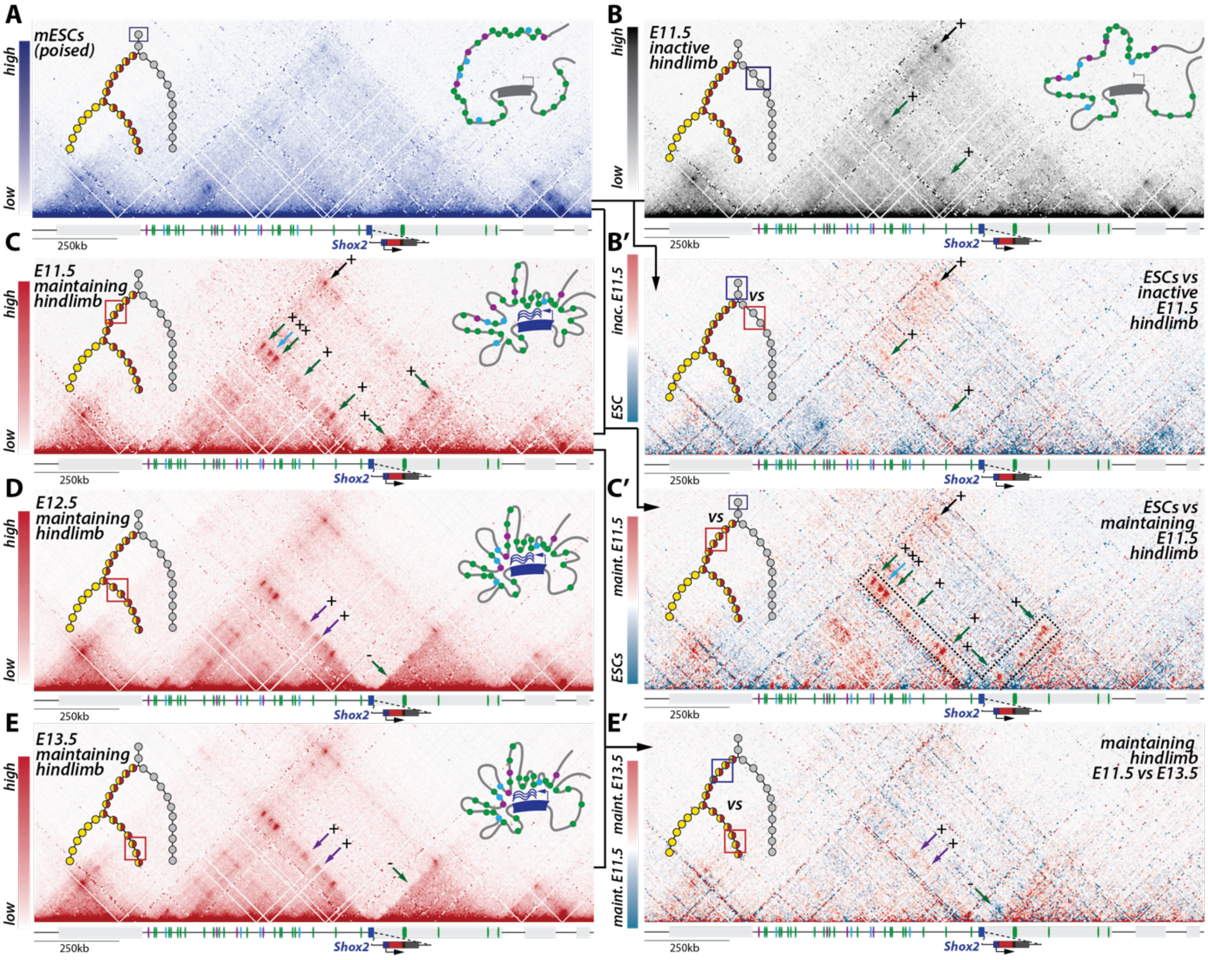

Supplementary Figure 6: *Shox2* locus 3D topology associates with active enhancer-promoter interactions in hindlimbs. In all Capture-HiC (C-HiC) maps (mm39: chr3:65,885,132-67,539,263), the upper left illustration represents the position of the investigated cells in the regulatory trajectory and the upper right one a model of the average 3D locus structure. The light grey box next to *Shox2* is the *Rsrc1* gene. **A**. C-HiC maps of the *Shox2* locus in *Shox2*^{trac} mESCs. Note a large TAD with few focal interaction points. **B**. C-HiC maps of the *Shox2* locus in E11.5 hindlimb FACS-sorted inactive cells. Note the formation of specific contacts with three early enhancers (green arrows) and increased loop contact between the two TAD borders (black arrow). **B'**. Subtraction C-HiC map between *Shox2*^{trac} E11.5 hindlimb FACS-sorted inactive cells and *Shox2*^{trac} mESC. **C-E**. C-HiC maps of the *Shox2* **C.** E11.5 **D**. E12.5, and E. E13.5 hindlimb FACS-sorted maintaining cells. **C'**. C-HiC subtraction maps between *Shox2*^{trac} mESCs and *Shox2*^{trac} E11.5 hindlimb FACS-sorted maintaining cells. **E'** C-HiC subtraction maps between E12.5 and E13.5 *Shox2*^{trac} FACS sorted hindlimb maintaining cells. Changes in enhancer-*Shox2* interactions are marked by colored arrows at each stage: Changes in enhancer-*Shox2* interactions are marked by colored arrows at each stage: green for early enhancers, purple for late enhancers, and light blue for common enhancers (as defined with vC in forelimb and hindlimb: see Supplementary Fig. 7). A plus sign (+) denotes a gain of interaction, and a minus sign (-) indicates a loss of interaction relative to the previous stage. Also note the increased separation between the two subTADs at the position of the *Shox2* gene body. Maps coordinates mm9; chr3:65,781,633-67,435,852. Maint. = maintaining; inac. = inactive.

Supplementary Figure 7

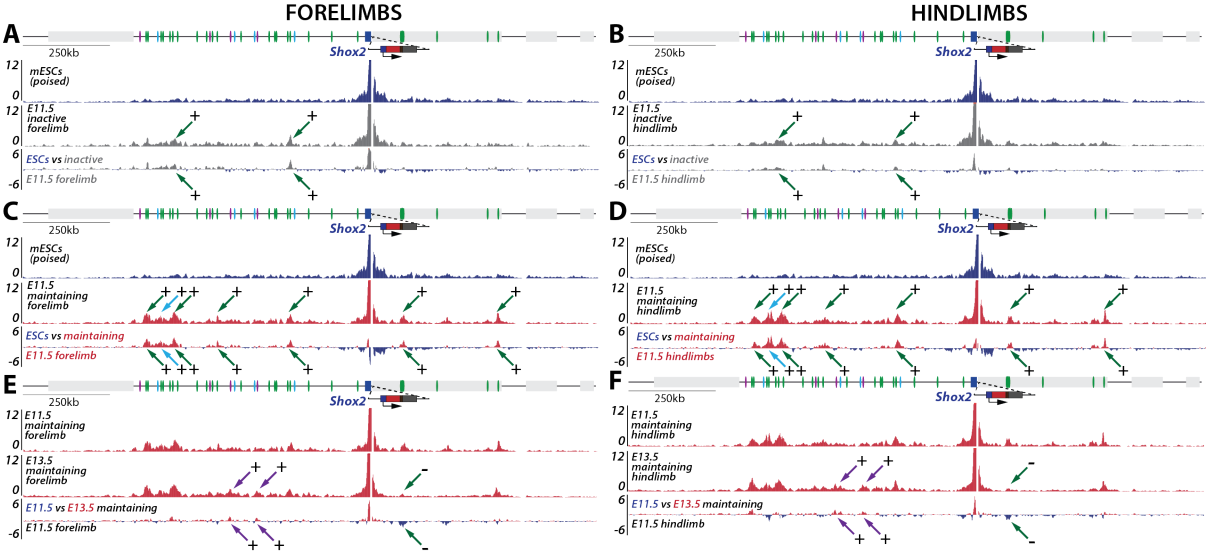

Supplementary Figure 7. Virtual capture-C (vC) from the *Shox2* promoter (mm39: chr3:66885043-66890041) in forelimbs (A-C-E) and hindlimbs (B-D-F). Interaction changes at enhancers are indicated with shadowed colored boxes. **A**. *Shox2* vC in mESC (top blue track), E11.5 inactive forelimb cells (middle grey track) and subtraction track (lower track). **B**. *Shox2* vC in mESC (top blue track), E11.5 inactive hindlimb cells (middle grey track) and subtraction track (lower track). **C**. *Shox2* vC in mESC (top blue track), E11.5 maintaining forelimb cells (middle red track) and subtraction track (lower track). **D**. *Shox2* vC in mESC (top blue track), E11.5 maintaining hindlimb cells (middle red track) and subtraction track (lower track). **E**. *Shox2* vC E11.5 maintaining forelimb cells (top red track), E13.5 maintaining forelimb cells (middle red track) and subtraction track (lower track). **F**. *Shox2* vC E11.5 maintaining hindlimb cells (top red track), E13.5 maintaining hindlimb cells (middle red track) and subtraction track (lower track). **A-F**: colored arrows pinpoint consistent interaction change between *Shox2* and putative enhancers in forelimb and hindlimb (green for early enhancers, purple for late enhancers, and light blue for common enhancers). A plus sign (+) denotes a gain of interaction, and a minus sign (-) indicates a loss of interaction relative to the previous stage. Coordinates of the region displayed mm39: chr3:65,885,132-67,539,263

Supplementary Figure 8

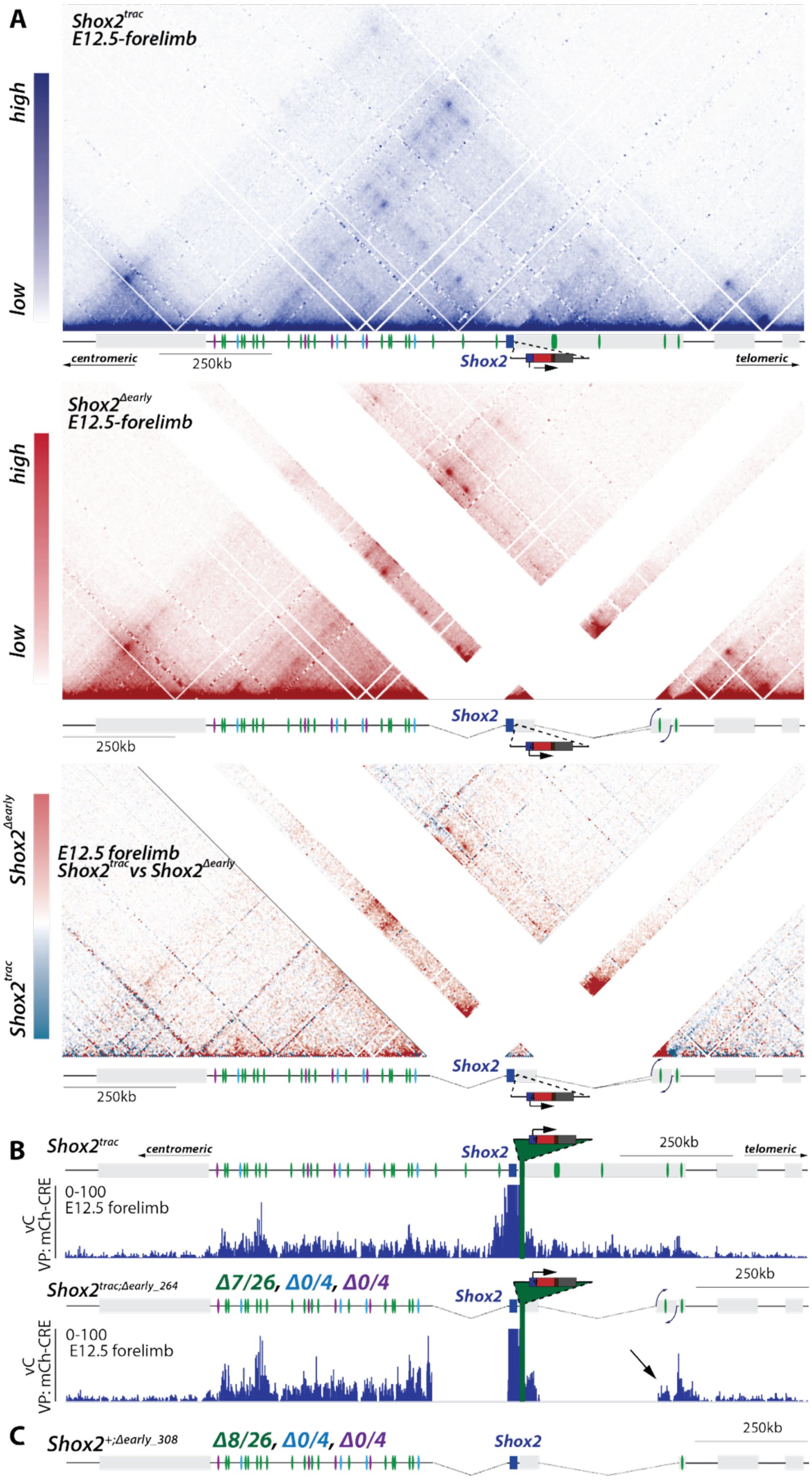

Supplementary Figure 8: **A**. C-HiC maps of *Shox2*^{trac} and *Shox2*$^{\Delta early}$ E12.5. Maps coordinates mm39: chr3:65,885,132-67,539,263. **B**. Virtual Capture-C (vC) analysis from the mCherry-P2A-CRE cassette (mm39_Shox2_dsmCherry_P2A_CRE_bGHpA_KI; chr3:66890192-66892123) in *Shox2*^{trac} and *Shox2*$^{\Delta early}$ E12.5 forelimbs revealed a 107kb centromeric deletion and a 264kb telomeric deletion) *Shox2*$^{+/\Delta early\_264)}$, with a remaining 44kb undeleted telomeric region (see black arrow). Further investigation showed the 44kb region is inverted (see Materials and Methods). **C**. The *Shox2*$^{+/\Delta early\_308}$ allele, in trans to the mCherry-P2A-CRE cassette, displays the expected deletion. **B-C** Coordinates of the region displayed mm39_Shox2_dsmCherry_P2A_CRE_bGHpA_KI; chr3:65,885,132-67,541,571

Supplementary Figure 9

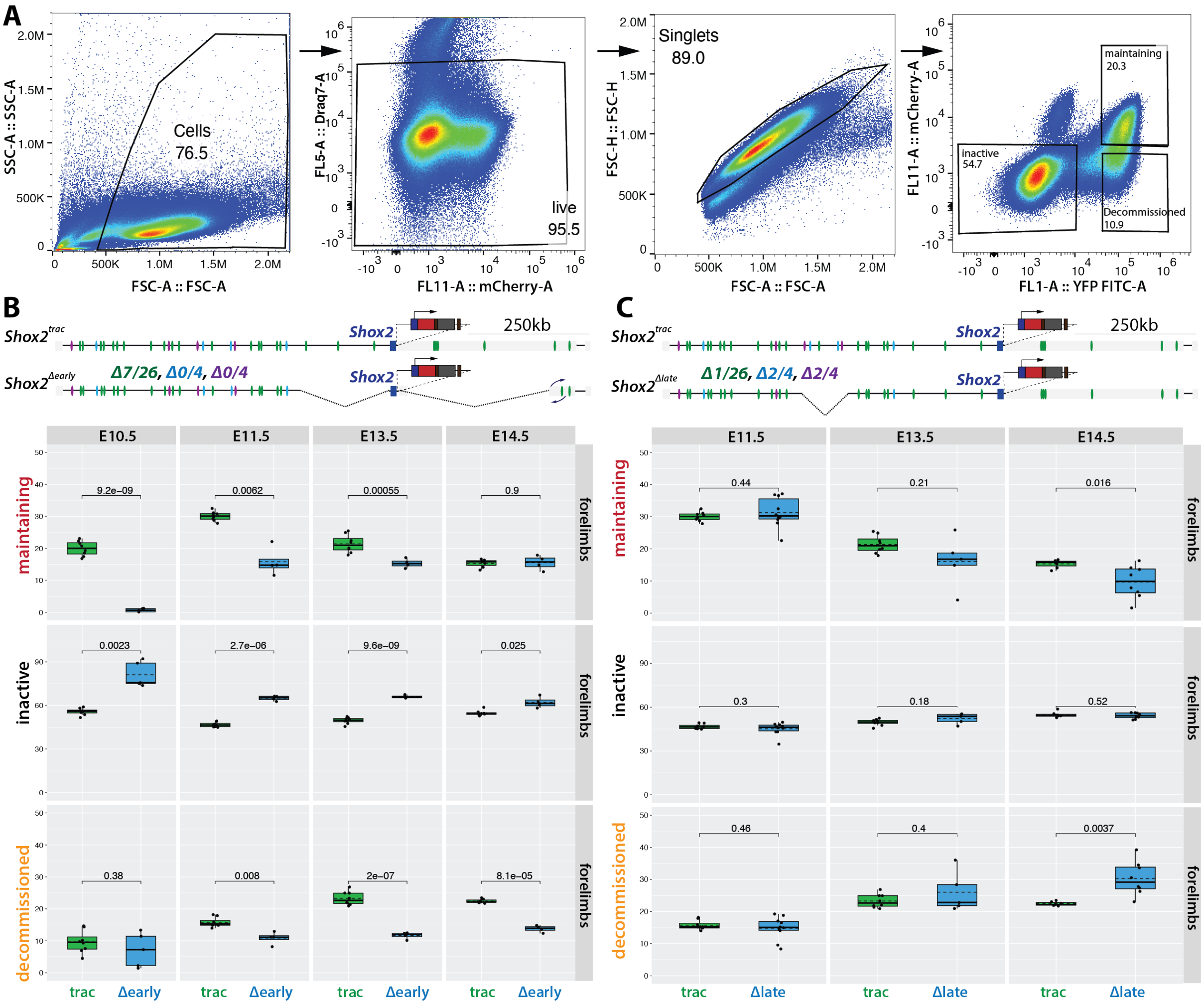

Supplementary Figure 9. A. Illustration of the gating strategy used to quantify cell groups (here represented by one replicate of *Shox2*^{trac} E10.5 forelimbs). **B**. Boxplot representation of maintaining, inactive, and decommissioned cell populations identified by flow cytometry analysis in *Shox2*^{trac} versus *Shox2*$^{\Delta early}$ forelimbs at E10.5, E11.5, E13.5, and E14.5. Upper schemes illustrate the *Shox2*$^{\Delta early}$ deletion allele lacking 7 out of 26 putative early enhancers (in green), while late (in purple) and common (light blue) putative enhancers remain intact. Each dot represents one replicate. **C.** Boxplot representation of maintaining, inactive, and decommissioned cell populations identified by flow cytometry analysis in *Shox2*^{trac} versus *Shox2*$^{\Delta late}$ forelimbs at E11.5, E13.5, and E14.5. Upper schemes illustrate schematic representation of the *Shox2*$^{\Delta late}$ deletion allele lacking 2 out 4 late (in purple), 2 out of 4 common (in light blue), 1 out of 26 early putative enhancers. Each dot represents one replicate. **B-C**: number of replicates for each forelimb genotype: *Shox2*^{trac} N at E10.5= 8, N at E11.5 = 8, N at E13.5 = 8, N at E14.5 = 7; *Shox2*$^{\Delta early}$ N at E10.5=5 , N at E11.5 = 4, N at E13.5 = 4 , N at E14.5 = 4; *Shox2*$^{\Delta late}$, N at E11.5 = 10, N at E13.5 = 5, N at E14.5 = 8. Boxplots: boxes indicate the first and third quartiles, the whiskers indicate ±1.5 × interquartile range, and the horizontal line within the boxes indicates the median. Statistical significance was assessed using two-sided T-tests on replicates. Source data are provided in Supplementary Data file 6.

Supplementary Figure 10

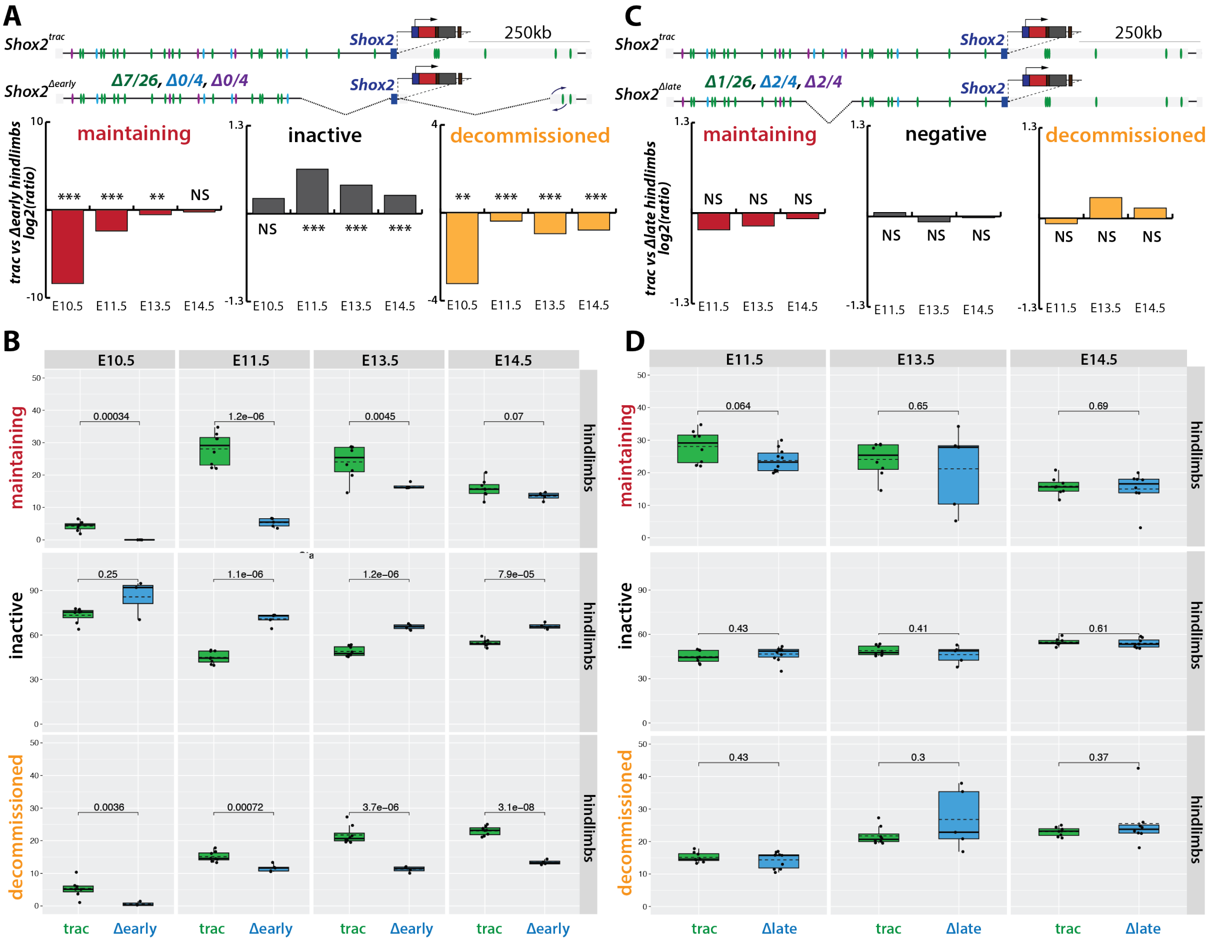

Supplementary Figure 10: A. Upper part of the panel illustrates *Shox2*$^{\Delta early}$ deletion allele lacking 7 out of 26 putative early enhancers (in green), while late (in purple) and common (light blue) putative enhancers remain intact. Bottom part shows log2 ratio of the proportion of maintaining, inactive, and decommissioned cell populations, identified by flow cytometry analysis, in *Shox2*^{trac} versus *Shox2*$^{\Delta early}$ hindlimbs at E10.5, E11.5, E13.5, and E14.5. NS= non-significant, *=p<0.05, **=p<0.01 and ***=p<0.001. **B**. Boxplot representation of maintaining, inactive, and decommissioned cell populations, identified by flow cytometry analysis, in *Shox2*^{trac} versus *Shox2*$^{\Delta early}$ hindlimbs at E10.5, E11.5, E13.5, and E14.5. Each dot represents one replicate. **C**. Upper part of the panel illustrates schematic representation of the *Shox2*$^{\Delta late}$ deletion allele lacking 2 out 4 late (in purple), 2 out of 4 common (in light blue), 1 out of 26 early putative enhancers. Bottom part shows log2 ratio of the proportion of maintaining, inactive, and decommissioned cell population, identified by flow cytometry analysis, in *Shox2*^{trac} versus *Shox2*$^{\Delta late}$ hindlimbs at E11.5, E13.5, and E14.5. NS= non-significant, *=p<0.05, **=p<0.01 and ***=p<0.001. **D**. Boxplot representation of maintaining, inactive, and decommissioned cell populations identified by flow cytometry analysis in *Shox2*^{trac} versus *Shox2*$^{\Delta late}$ hindlimbs at E11.5, E13.5, and E14.5. Each dot represents one replicate. **B-D**: number of replicates for each hindlimb genotype: *Shox2*^{trac} N at E10.5= 7, N at E11.5 =8, N at E13.5 = 8, N at E14.5 = 8; *Shox2*$^{\Delta early}$ N at E10.5 = 3 , N at E11.5 = 5, N at E13.5 = 4, N at E14.5 = 4; *Shox2*$^{\Delta late}$, N at E11.5 = 10, N at E13.5 = 5, N at E14.5 = 8. Boxplots: boxes indicate the first and third quartiles, the whiskers indicate ±1.5 × interquartile range, and the horizontal line within the boxes indicates the median. Statistical significance was assessed using two-sided T-tests on replicates. Source data are provided in Supplementary Data file 6.

Supplementary Figure 11

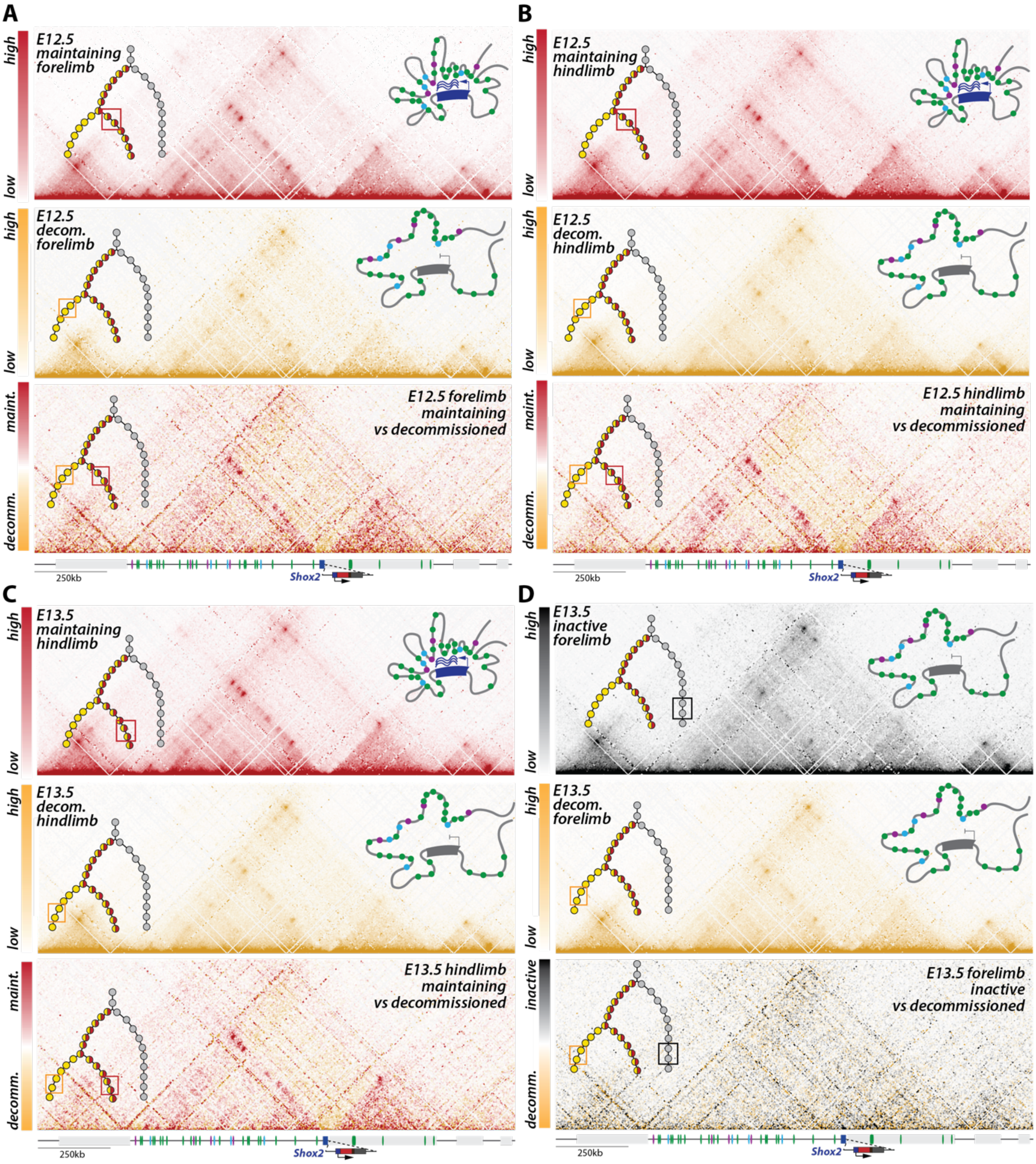

Supplementary Figure 11. In each panel: the upper left illustration represents the position of the investigated cells in the regulatory trajectory; the upper right one a model of the average 3D locus structure. Maps coordinates mm39: chr3:65,781,633-67,435,852. Maint. = maintaining; decomm. = decommissioned. **A-C**: C-HiC maps of FACS-sorted maintaining cells (top), decommissioned cells (middle) and subtraction map between both (bottom) in **A.** E12.5 forelimb, **B.** E12.5 hindlimb and **C.** E13.5 hindlimb. **D**. C-HiC maps of FACS-sorted hindlimb E12.5 maintaining cells (top), decommissioned cells (middle), and subtraction map between both (bottom).

Supplementary Figure 12

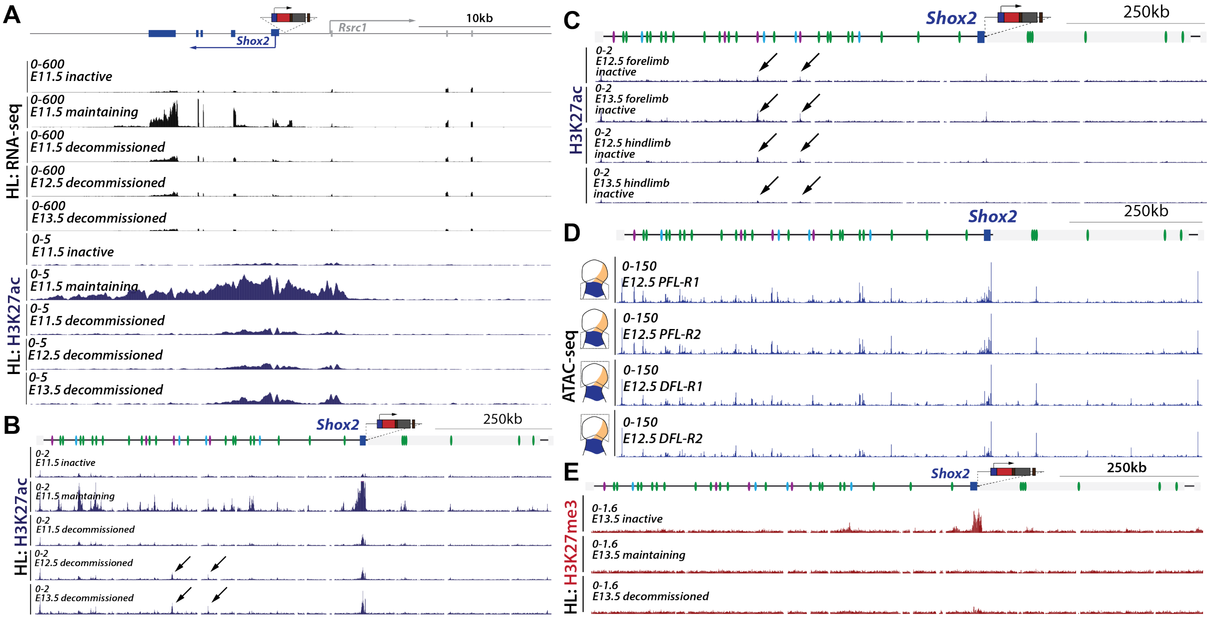

Supplementary Figure 12: A. RNA-seq, H3K27ac ChIP-seq tracks in early hindlimb inactive, maintaining, decommissioned and late decommissioned FACS-sorted cells at the *Shox2* and *Rsrc1* promoter regions (mm39: chr3:66,870,000-66,910,000). **B**. Hindlimb H3K27ac ChIP-seq tracks in early inactive, maintaining, decommissioned and late decommissioned FACS-sorted cells over the *Shox2* regulatory landscape (mm39: chr3:66,190,000-67,290,000). Note the loss of H3K27ac at enhancers in decommissioned cells. Note that two of the fours late enhancers show activity in decommissioned cells (black arrows). **C**. Fore and hindlimb H3K27ac ChIP-seq tracks in early and late inactive cells over the *Shox2* regulatory landscape (mm39: chr3:66,190,000-67,290,000). Note that two of the four late enhancers show activity in inactive cells (black arrows). **D**. Proximal (PFL) and distal (DFL) forelimb E12.5 ATAC-seq profiles from[4]. Each condition is in two replicates. Note the similarity between proximal (*Shox2*-active) and distal (*Shox2*-inactive) limbs. **E**. E13.5 hindlimb H3K27me3 ChIP-seq tracks in inactive, maintaining and decommissioned FACS-sorted cells over the *Shox2* regulatory landscape (mm39: chr3:66,190,000-67,290,000). Note the weaker H3K27me3 enrichment in decommissioned compared to inactive cells.

Supplementary Figure 13

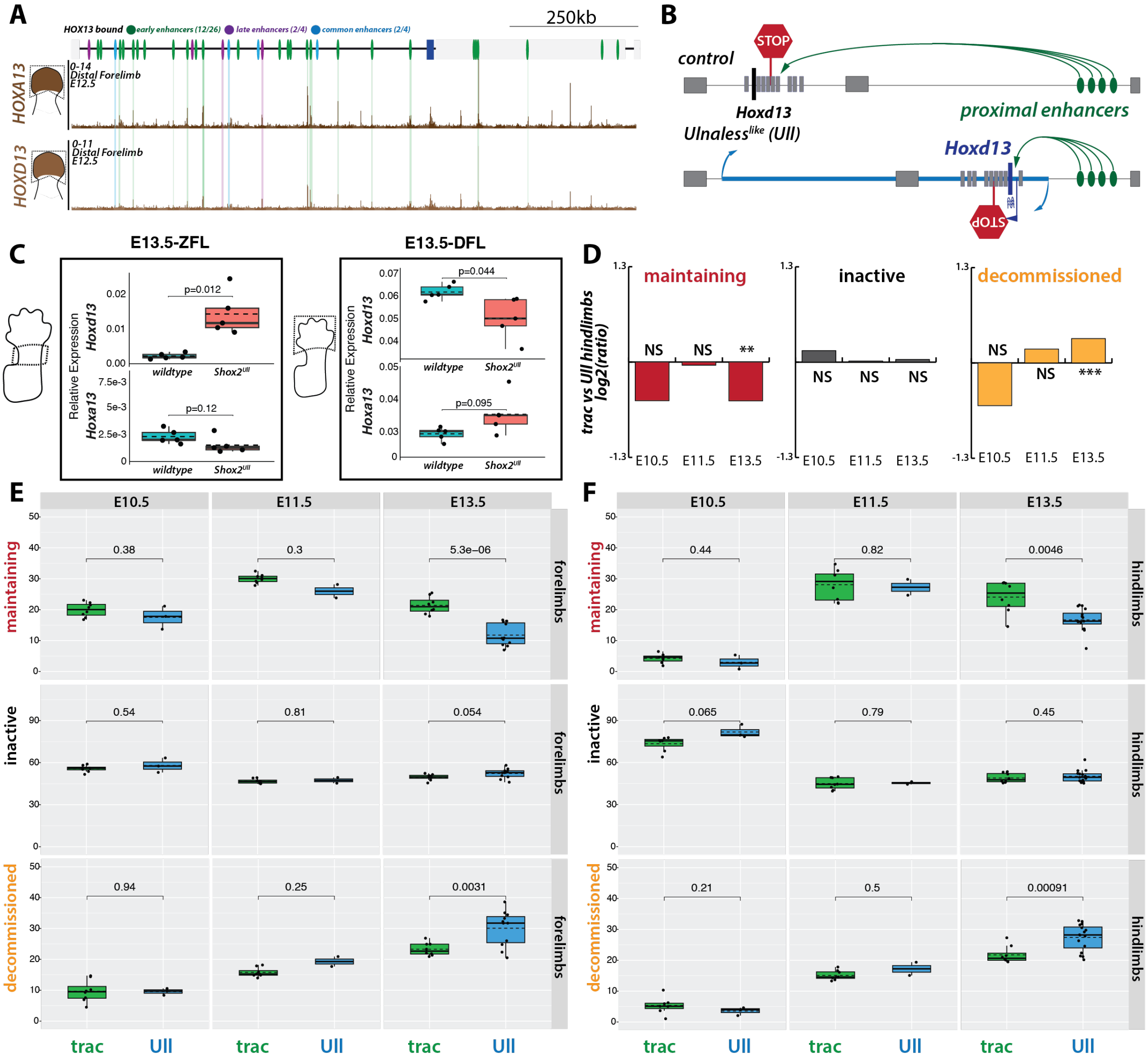

Supplementary Figure 13. A. *HOXA/D13* ChIP-seq in distal forelimb (DFL) at E12.5 from[5]. **B**. Schematic illustration of the engineered Ulnaless inversion allele at the Hoxd locus, showing how the inversion brings proximal enhancers into close proximity with *Hoxd13*, leading to its misexpression. **C**. *Hoxd13* and *Hoxa13* RT-qPCR in wildtype and $Shox2^{Ull}$ E13.5 micro-dissected zeugopod and distal forelimbs (ZFL and DFL). Note the increase of *Hoxd13* in ZFL, but not of *Hoxa13* that is used here as a marker of correct micro-dissection. *Hoxd13*, but not *Hoxa13*, slightly decreases in $Shox2^{Ull}$ DFL due to the disconnection to its distal enhancers. Paired t-tests were utilized to calculate p-values from replicates (N=5, each dot represent a replicate). Source data are provided in the Source Data file. **D**. Log2 ratio between the proportion of hindlimb $Shox2^{trac}$ and $Shox2^{Ull}$ initiating, maintaining and decommissioned cell populations, identified by flow cytometry analysis, at E10.5, E11.5 and E13.5. T-tests were utilized to calculate p-values from replicates (See panel F). NS= non-significant, *=p<0.05, **=p<0.01 and ***=p<0.001. **E**. Boxplot representation of maintaining, inactive, and decommissioned cell populations, identified by flow cytometry analysis, in $Shox2^{trac}$ versus $Shox2^{Ull}$ forelimbs at E10.5, E11.5 and E13.5. Each dot represents one replicate. **F**. Boxplot representation of maintaining, inactive, and decommissioned cell populations, identified by flow cytometry analysis, in $Shox2^{trac}$ versus $Shox2^{Ull}$ hindlimbs at E11.5, and E13.5. Each dot represents one replicate. **E-F**, number of replicates for each forelimb genotype: $Shox2^{trac}$ N at E10.5= 8, N at E11.5 = 8, N at E13.5 = 8; $Shox2^{Ull}$ N at E10.5= 3, N at E11.5 = 2, N at E13.5 = 11; and for each hindlimb genotype: $Shox2^{trac}$ N at E10.5= 7, N at E11.5 =8, N at E13.5 = 8; $Shox2^{Ull}$ N at E10.5= 3, N at E11.5 = 2, N at E13.5 = 16. Boxplots: boxes indicate the first and third quartiles, the whiskers indicate ±1.5 × interquartile range, and the horizontal line within the boxes indicates the median. Statistical significance was assessed using two-sided T-tests on replicates. Source data are provided in Supplementary Data file 6.

Supplementary Figure 14

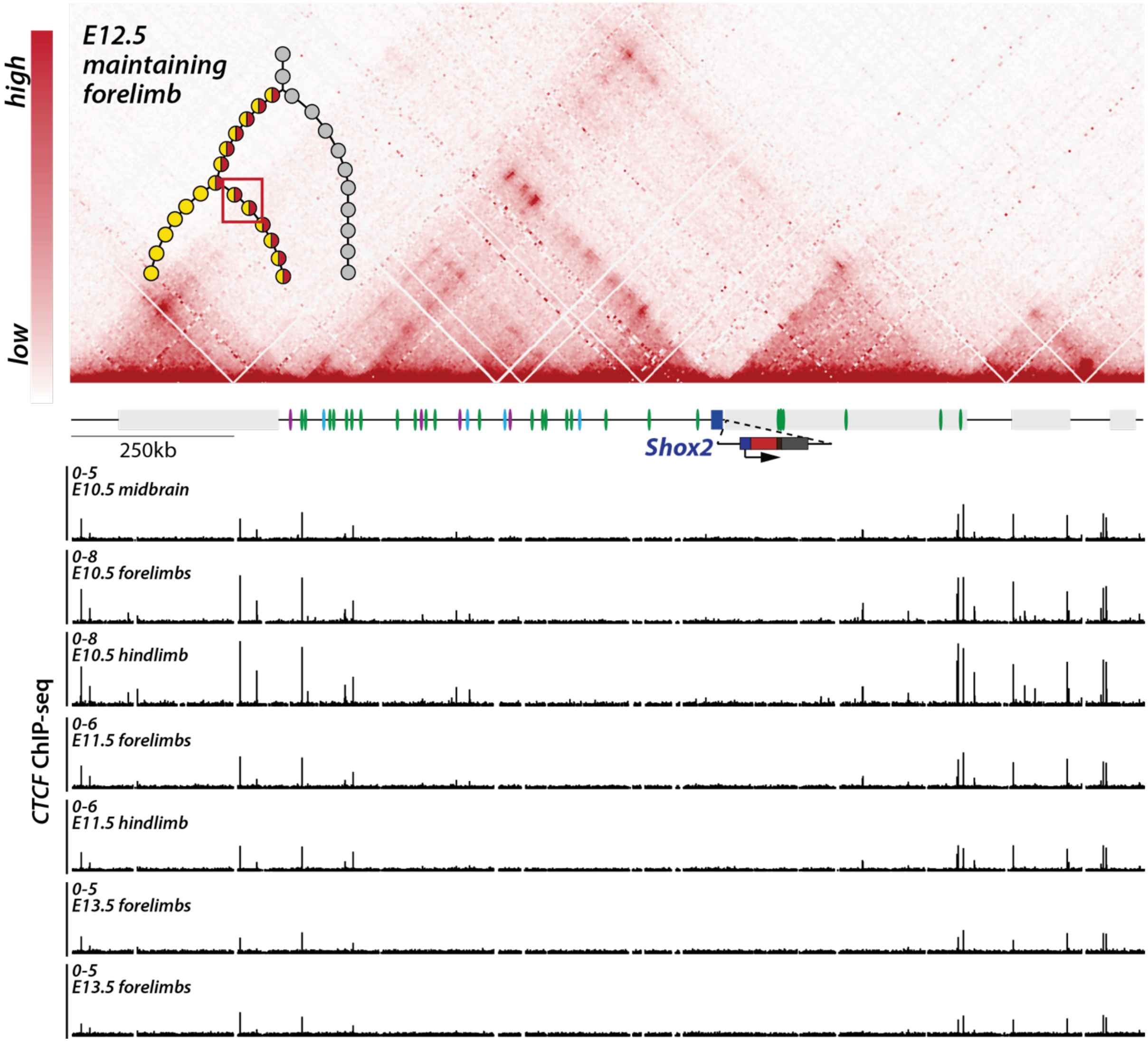

Supplementary Figure 14. Binding of *CTCF* at the *Shox2* locus. Above: C-HiC map of E12.5 FACS-sorted maintaining forelimb cells. Below: *CTCF* ChIP-seq tracks of E10.5 midbrain, E10.5, E11.5 and E13.5 fore and hindlimbs[1]. Maps coordinates mm39: chr3:65,781,633-67,435,852.

References

1. Andrey, G. *et al.* Characterization of hundreds of regulatory landscapes in developing limbs reveals two regimes of chromatin folding. *Genome Res* **27**, 223–233 (2017).
2. Abassah-Oppong, S. *et al.* A gene desert required for regulatory control of pleiotropic Shox2 expression and embryonic survival. *Nat Commun* **15**, 8793 (2024).
3. Osterwalder, M. *et al.* Enhancer redundancy provides phenotypic robustness in mammalian development. *Nature* **554**, 239–243 (2018).
4. Bolt, C.C. *et al.* Context-dependent enhancer function revealed by targeted inter-TAD relocation. *Nat Commun* **13**, 3488 (2022).
5. Sheth, R. *et al.* Distal Limb Patterning Requires Modulation of cis-Regulatory Activities by HOX13. *Cell Rep* **17**, 2913–2926 (2016).
