# Supplementary material for: Temporal constraints on enhancer usage shape the regulation of limb gene transcription
Source: Nat Commun. 2026 Jan 12;17:5. doi: 10.1038/s41467-025-66055-6 (PMC12795824; doi:10.1038/s41467-025-66055-6)
Supplement: Supplementary file 2 — Description of Additional Supplementary Files [file 41467_2025_66055_MOESM2_ESM.pdf]

File name: Supplementary Data 1
Description: Reanalysis of limb RNA-seq and ChIP-seq datasets from Andrey et al., 2017[1]. Includes FPKM values of selected genes in E10.5 and E13.5 forelimbs, identified enhancers in forelimbs, mm39 capture-C interaction domains, and the categories of enhancers within each domain.

File name: Supplementary Data 2
Description: Differential expression analysis of E12.5 *Shox2*^{trac} and wildtype control entire forelimbs. FPKM and normalized counts are provided. Pairwise DESeq2 was performed for differential analysis (*padj* is the FDR-corrected, two-tailed p-value using the Benjamini-Hochberg method).

File name: Supplementary Data 3
Description: scRNA-seq marker genes for subsetted and re-clustered mesenchymal clusters. Statistics were performed using two-sided Wilcoxon Rank Sum test, p-value adjustment was performed using Bonferroni correction based on the total number of genes in the dataset.

File name: Supplementary Data 4
Description: Combined results of the RNA-seq differential expression analyses in *Shox2*^{trac} fore and hindlimbs at different stages presented in Figure 2 and Supplementary Figure S4. Normalized FPKMs in each sorted cell categories (Maint: Maintaining, Decomm: Decommissioned, Inact: Inactive for stages E10.5, E11.5, E12.5 and E13.5) for all datasets are provided, followed by DESeq2 pairwise comparisons (*padj* is the FDR-corrected, two-tailed p-value using the Benjamini-Hochberg method).

File name: Supplementary Data 5
Description: Early, common and late putative enhancers classification on *Shox2*^{trac} FACS sorted maintaining forelimb datasets at the *Shox2* locus. Includes for each enhancer: categories, coordinates, size, normalized read count at E10.5, E11.5, E12.5 and E13.5, and overlap with previously published enhancers.

File name: Supplementary Data 6
Description: Proportion of each cell FACS analysed categories in various allelic configuration in fore and hindlimbs and at different stage. Includes *Shox2*$^{\Delta early}$: del(early), *Shox2*$^{\Delta late}$: del(late), *Shox2*^{Ull}: Ulnaless like. Ratios calculated based on this proportion and shown in Figures 5C, 5G, 7C and Supplementary Figure 13D are also included

File name: Supplementary Data 7
Description: Combined differential RNA-seq expression analysis including FPKM and normalized counts are provided. Pairwise DESeq2 was performed for differential analysis (*padj* is the FDR-corrected, two-tailed p-value using the Benjamini-Hochberg method).: Includes E10.5 and E14.5 *Shox2*^{trac} vs *Shox2*$^{\Delta early}$ entire forelimbs; E11.5 and E14.5 *Shox2*^{trac} vs *Shox2*$^{\Delta late}$ entire forelimbs; E14.5 maintained and decommissioned sorted cells from *Shox2*^{trac} vs *Shox2*$^{\Delta late}$.

File name: Supplementary Data 8

Description: sgRNAs for CRISPR/Cas9, primers for PCR genotyping, primers for qPCR genotyping and primers for RT-qPCR.

1. Andrey, G. *et al.* Characterization of hundreds of regulatory landscapes in developing limbs reveals two regimes of chromatin folding. *Genome Res* **27**, 223–233 (2017).
