# Supplementary material for: Temporal constraints on enhancer usage shape the regulation of limb gene transcription
Source: Nat Commun. 2026 Jan 12;17:5. doi: 10.1038/s41467-025-66055-6 (PMC12795824; doi:10.1038/s41467-025-66055-6)
Supplement: Supplementary file 11 — Reporting Summary [file 41467_2025_66055_MOESM11_ESM.pdf]

nature portfolio

Corresponding author(s): Guillaume Andrey

Last updated by author(s): Sep 19, 2025

Reporting Summary

Nature Portfolio wishes to improve the reproducibility of the work that we publish. This form provides structure for consistency and transparency in reporting. For further information on Nature Portfolio policies, see our Editorial Policies and the Editorial Policy Checklist.

Statistics

For all statistical analyses, confirm that the following items are present in the figure legend, table legend, main text, or Methods section.

n/a	Confirmed	
☐	☒	The exact sample size (*n*) for each experimental group/condition, given as a discrete number and unit of measurement
☐	☒	A statement on whether measurements were taken from distinct samples or whether the same sample was measured repeatedly
☐	☒	The statistical test(s) used AND whether they are one- or two-sided<br>*Only common tests should be described solely by name; describe more complex techniques in the Methods section.*
☒	☐	A description of all covariates tested
☐	☒	A description of any assumptions or corrections, such as tests of normality and adjustment for multiple comparisons
☐	☒	A full description of the statistical parameters including central tendency (e.g. means) or other basic estimates (e.g. regression coefficient) AND variation (e.g. standard deviation) or associated estimates of uncertainty (e.g. confidence intervals)
☐	☒	For null hypothesis testing, the test statistic (e.g. *F*, *t*, *r*) with confidence intervals, effect sizes, degrees of freedom and *P* value noted<br>*Give P values as exact values whenever suitable.*
☒	☐	For Bayesian analysis, information on the choice of priors and Markov chain Monte Carlo settings
☒	☐	For hierarchical and complex designs, identification of the appropriate level for tests and full reporting of outcomes
☒	☐	Estimates of effect sizes (e.g. Cohen's *d*, Pearson's *r*), indicating how they were calculated

Our web collection on statistics for biologists contains articles on many of the points above.

Software and code

Policy information about availability of computer code

Data collection

No commercial, open source and custom code was used to collect the data in this study.

Data analysis

1 - Custom Genome
ChIP-seq, RNA-seq and scRNA-seq datasets generated in this study were aligned to a customized version of the GRCm39/mm39 assembly incorporating the dmCherry-P2A-CRE and floxed-SV40pASTOP-EYFP cassettes as artificial chromosomes, that we termed as GRCmm39/ mm39_dsmCherry_P2A_CRE_EYFP genome and it is available on https://zenodo.org/records/11219861. NGS datasets downloaded from GEO and re-analyzed in this study (Andrey et al., 2017; Sheth et al., 2016) were aligned to the normal GRCm39/mm39 assembly. For annotation, GTF files sourced from ENSEMBL GRCm39 release 104 were used, with a filtering process applied to exclude read-through/overlapping transcripts. Only transcripts annotated as 'protein-coding' for their respective genes were retained, while those flagged as 'retained_intron', 'nonsense-mediated decay', etc., were discarded. This filtration aimed to retain only unambiguous exons, mitigating potential quantitative biases during data analysis conducted using STAR/Cufflinks (Amandio et al., 2016). This GTF file can be also found on https://zenodo.org/ records/11219861. C-HiC datasets were aligned to the regular NCBI37/mm9.

2 - scRNA-seq
Demultiplexing, alignment, filtering of barcodes, and UMI counting were executed using the 10× Genomics Cell Ranger software (version 6.1.2) in accordance with the manufacturer's guidelines, default settings and custom genome GRCmm39/ mm39_dsmCherry_P2A_CRE_EYFP built following using the cellranger mkref pipeline. Cell Ranger output files for each dataset were further processed using the velocyto run10x command from the velocyto.py tool (version 0.17.17) in Python (version 3.9.12) with our custom genome and the UCSC genome browser repeat masker.gtf file to mask expressed repetitive elements to generate a loom file for each sample. Each resulting loom matrix, comprising spliced/unspliced/ambiguous reads, was individually imported into R (version 4.1.2) using the Read Velocity function from the Seurat Wrappers package (version 0.3.0). Simultaneously, feature-filtered output matrices obtained from Cell Ranger were loaded into R separately

through the Read10X function of the Seurat package (version 4.2.1). Subsequently, the spliced, unspliced, ambiguous, and RNA feature data were combined into a single matrix for each dataset. Following this, each matrix was transformed into a Seurat object using the Seurat package. Consequently, for each sample, a single Seurat object was obtained, encompassing four assays. Three of these assays (spliced, unspliced, and ambiguous) were used for downstream RNA velocities estimations, while the RNA feature assay was employed for subsequent gene expression analysis among the samples, as detailed below. Quality control and pre-processing of each Seurat object for our samples were conducted based on the following criteria. Cells expressing fewer than 200 genes or exhibiting more that 7500 features were excluded from the analysis. Additionally, we calculated the proportion of reads mapping to the mitochondrial genome, filtering out cells with a mitochondrial content exceeding 5%, as elevated levels of mitochondrial mRNA have been linked to cell death. Conversely, cells with a mitochondrial content lower than 0.5% were also excluded, as our observations suggest that these cells likely originate from blood cells, possibly due to the dissection protocol. After filtering each dataset was individually normalized using the default parameters provided by Seurat for the LogNormalize method and applying it to the RNA features assay. Subsequently, we calculated the most variable features excluding the CRE, EYFP and dmCherry artificial genes added on our custom genome from the list of variable genes to avoid that they drive the PCA. Then, scaling was performed via linear transformation and scaled data were then employed for principal component analysis (PCA), utilizing the default 50 principal components (PCs). Additionally, non-linear dimensional reduction was conducted using Uniform Manifold Approximation Projection (UMAP) with 1:50 dimensions utilized as input. Cell doublet identification and features annotation: Pre-processed and normalized datasets were individually examined to detect putative doublet cells. Doublets identified in each dataset were subsequently excluded using the DoubletFinder R package (version 2.0.3). The doublet rate (nExp parameter) utilized was estimated based on the number of cells captured and pK parameter was estimated following the strategy defined in the package, resulting in the following values: Shox2trac Hindlimb E10.5, nExp= 89, pK=0.3; E11.5 nExp= 98, pK=0.16; E12.5 nExp= 71, pK=0.25; E13.5 nExp= 88, pK=0.1. After removing doublets, counts for CRE, dmCherry, Shox2, and EYFP per cell were estimated. Cells were then classified as positive for each of these genes if they had at least one count, and negative otherwise. Cells positive for Shox2, negative for EYFP, and either positive or negative for dmCherry-P2A-CRE were classified as initiating. Those positive for both Shox2 and EYFP, regardless of dmCherry-P2A-CRE status, were classified as maintaining. Cells negative for both Shox2 and dmCherry-P2A-CRE but positive for EYFP were considered decommissioned. Cells negative for all three genes were marked as inactive, and any remaining combinations of gene expression fell under the class "other". A new metadata column containing the classification of the cells was then created. All datasets were then merged into a single Seurat object without undergoing integration, allowing for ensemble downstream analysis of the four datasets. Subsequently, no batch effect was observed in this merged dataset. Afterward, we applied the SCTransform normalization protocol to our newly merged Seurat object, utilizing default parameters, over the spliced assay. Since we observed, during individual dataset analysis, that a portion of the variance was attributable to cell-cycle genes, we assigned cell cycle score using the CellCycleScoring function implemented in Seurat. As we also observed that sample variance by a stage effect we regress out the cell-cycle heterogeneity and stage variability by applying SCTransform normalization method to our merged object, using the spliced assay as the source, and incorporating the calculated cell-cycle scores (S.Score and G2M.Scores) and the stage metadata information as variables to regress, in addition to the default settings. Subseqeuntly, we excluded dmCherry, CRE and EYFP, if they were present from the variable genes to avoid that they drive the PCA. Following the regression step, cells were clustered using the standard steps of the SCTransform Seurat workflow. Briefly, PCA (npcs=50), UMAP (dims=1:50, n.neighbors = 50), and nearest neighbors were calculated. Clusters were identified using the Seurat FindClusters function with default parameters and a resolution of 0.7, resulting in the definition of 21 clusters. Clusters presenting similar features profiles were combined, reducing the final number of clusters identified to 6 clusters. The mesenchyme (comprising 13 out of the 21 clusters), epithelium (consisting of 3 out of 21), muscle (comprising 2 out 21), and endothelium, immune Cells, and blood Cells clusters each represented by only 1 cluster. The presence of expected identity markers in the new clustering was confirmed by running the FindMarkers function with default parameters and using grouping.var = "stage" and only.pos = TRUE. Given the focus of this study on populations expressing Shox2, we subsetted and re-clustering the mesenchyme cluster. To do, after applying subset function to the "Mesenchyme" UMAP embedding was computed with the following parameters: dims=c(1:10), n.neighbors=30L, min.dist= 0.5, metric="euclidean", spread=1, while keeping all other parameters at their default values. Subsequently, the cluster resolution after finding neighbors was set at 1.1 to reveal subpopulations. We observed 18 mesenchyme subpopulations, each named based on their identity genes. Identity markers were identified using the FindMarkers function on the RNA assay, with grouping.var = "stage", only.pos =TRUE, logfc.threshold=0.3, min.diff.pct=0.1, and all other parameters set to default values. Clusters presenting similar features profiles were combined, reducing the final number of clusters identified to 15 clusters. late proximal progenitors (comprised 3 out of the 18 clusters) and irregular connective tissue (contained 2 out the 18) the other clusters remained represented by 1 cluster. Final identity markers for the new clustering was assessed by running the FindMarkers function on the RNA assay, only.pos =TRUE, logfc.threshold=0.5, pseudocount.use = 0, min.diff.pct=0.1, and all other parameters set to default values. We then performed RNA-velocity analysis on all combined datasets by exporting Seurat object as h5Seurat files using the SeuratDisk package (version 0.0.0.90) and for using it as input in Scvelo (version 0.2.5) in Python (version 3.9.16). Then the standard protocol described in scVelo was followed, with the exception of using npcs=10 and n.neighbors= 30, to match the parameters used for UMAP embedding in Seurat. Shox2 velocity was computed by running velocyto package for R (version 0.6) with default parameters on the Seurat Object to generate an embedding file from which Shox2 was only plotted using ggplot2 (version 3.4.4). FeaturePlot and VlnPlot were generated from the RNA assay of the Seurat objects. FeaturePlot and Dimplot were produced using default Seurat parameters. Density UMAP plots were produced using the Nebulosa v1.4.0 package(Alquicira-Hernandez and Powell, 2021). Cell proportions were calculated using the prop.table tool from the base R package (version 4.1.2) followed by plotting using ggplot2 (version 3.4.4).

3 - RNA-seq

FASTQ files from FACS-sorted cells or entire limbs generated in this study were processed using CutAdapt v1.18 to trim NextSeq adapter sequences and low-quality bases employing the adapter sequence -a CTGTCTCTTATACACATCTCCGAGCCCACGAGAC with a quality cutoff of 30 (-q30) and a minimum length requirement of 15 bases (-m15). In the case of the samples from GEO datasets that we wanted to reanalyze (Andrey et al., 2017) CutAdapt was used to trim TruSeq adapter sequences and low-quality bases , using the following parameters -a GATCGGAAGAGCACACGTCTGAACTCCAGTCAC, -q30 and -m15). Unstranded reads were then mapped on the customized genome GRCm39/ mm39_dsmCherry_P2A_CRE_EYFP, in the case of the datasets produced in this study, or to the GRCm39/mm39, in the case of the reanalyzed dataset. The STAR version 2.7.2b was then used together with the filtered GTF file generated for this study (see Custom genome for NGS analyses in this Material and Methods section) for accurate gene quantification using tailored settings (--outSAMstrandField intronMotif --sjdbOverhang '99' --sjdbGTFfile $gtfFile --quantMode GeneCounts --outFilterType BySJout --outFilterMultimapNmax 20 --outFilterMismatchNmax 999 --outFilterMismatchNoverReadLmax 0.04 --alignIntronMin 20 --alignIntronMax 1000000 --alignMatesGapMax 1000000 --alignSJoverhangMin 8 --alignSJDBoverhangMin 1). FPKM values were then determined by Cufflinks version 2.2.1 using the filtered GTF file generated for this study and tailored settings (--max-bundle-length 10000000 --max-bundle-frags 100000000 -- multi-read-correct --library-type "fr-firststrand" --no-effective-length-correction -M MTmouse.gtf). Then, Normalized FPKM were computed by determining coefficients extrapolated from a set of 1000 house-keeping genes stably expressed across the series of compared RNA-seq datasets. Differential expression analysis was performed using DEseq2 R package (version 1.34.0) with the Wald test for comparisons across samples and multiple test correction using the FDR/Benjamini-Hochberg test.

4 - ChIP-seq

Reads from ChIP-seq sequencing either from datasets generated for this study or from GEO datasets that we wanted to reanalyze (Andrey et al., 2017; Sheth et al., 2016) were processed first using CutAdapt version 1.18 to trim TruSeq or Nextseq adapter sequences and low-quality bases , specifying the adapter sequence with -a GATCGGAAGAGCACACGTCTGAACTCCAGTCAC (for TruSeq) or -a CTGTCTCTTATACACATCTCCGAGCCCACGAGAC (for Nextseq), a quality threshold of 30 with -q30, and a minimum length of 15 bases with -m15. Then reads were mapped to our GRCm39/mm39_dsmCherry_P2A_CRE_EYFP customized mouse genome, in the case of the datasets generated for this study, or to the GRCm39/mm39, in the case of the reanalyzed datasets, using Bowtie2 version 2.3.5.1 with its default settings. Subsequently, only reads with a mapping quality score (MAPQ) of 30 or higher were retained, as filtered with SAMtools view version 1.10. For coverage and peak analysis, reads were extended by 200 base pairs and processed using MACS2 version 2.2.7.1 with the parameters --broad --nolambda --broad-cutoff 0.05 --nomodel --gsize mm --extsize 200 -B 2 for broad peak calling, in the case of H3K27ac and H3K27me3 ChIP-seq data produced from the FACS sorted cells for this study. For reanalyzed datasets coverage and peak analysis, reads were extended by 200 base pairs and processed using MACS2 version 2.2.7.1 with the parameters --call-summits --nomodel --extsize 200 -B 2 for narrow peak calling. The coverage normalization was performed by MACS2, adjusting for the total millions of tags used in the analysis.

5 - Early, common, late putative enhancers classification on entire forelimb datasets

RNA-seq FASTQ files from two replicates of entire forelimbs at E10.5 and E13.5 (Andrey et al., 2017) were re-analyzed following the RNA-seq pipeline previously described. Genes related to limb development were selected for downstream analysis. Average of normalized FPKM values was calculated and used to compute the ratio among E10.5 and E13.5 datasets. Then, since we were interested in genes having stable expression between E10.5 and E13.5, they were filtered to keep those with FPKM values bigger than 5, at both stages, and we excluded genes having a fold change larger than 3 between the two stages. By applying this filtering 90 genes were selected. To analyze putative enhancers ChIP-seq H3K27Ac datasets of entire forelimbs at E10.5 and E13.5 (Andrey et al., 2017) were first reanalyzed following the ChIP-seq pipeline previously described in this material and methods section. H3K27ac MACS2 narrowpeaks were then restricted within the interaction domain defined by promoter Capture-C (Andrey et al., 2017) of the 90 filtered genes, using bedtools (version v2.30.0) intersect function. Then, H3K27Ac peaks around gene promoters were excluded by filtering against a -2kb/500bp window centered at the transcription start site of coding genes using again bedtools intersect. At the Shox2 locus, peak called at the alternative Veph1 alternative promoter was manually excluded. Remaining peaks were extended by +/- 300bp, using bedtools slope and merge function. H3K27Ac peaks were then classified as putative common enhancers when present in both E10.5 and E13.5 using bedtools intersect. H3K27Ac peaks present only in the E10.5 dataset were classified as putative early enhancers while H3K27Ac peaks present only in the E13.5 dataset were classified as putative late enhancers. Putative enhancers were then assigned to gene interaction domain. In those cases, where putative enhancers were within the overlapping region of two domains putative enhancers were assigned to the two loci. H3K27Ac peaks classified in the three categories were then processed to calculate scores using the compareMatrix function from bedtools. This matrix was then used to produce a heatmap (tornado plot) of the H3K27Ac peaks per category using plotHeatmap function from bedtools.

6 - Early, common, late putative enhancers classification on FACS sorted maintaining forelimb datasets

MACS2 BroadPeak files from FACS sorted maintaining cells from forelimbs at E10.5, E11.5, E12.5 and E13.5 were used to build bed files. These files were used to merge peaks within 600bp of each other using bedtools (version v2.30.0). Then, bedops (version 2.4.41) --merge operation was used to flatten all disjoint, overlapping, and adjoining element regions into contiguous, disjoint regions peaks among the four different stages. Subsequently, peaks were extended by +/- 300bp, using bedtools slope function. Since, we wanted only to explore the putative enhancers of Shox2 locus using bedtools intersect we selected only the region with the following coordinates mm39 chr3:65,885,132-67,539,263, in that way we created a list of peaks of interest. Peaks falling on gene promoters were manually excluded. Then as we wanted to stablish our new early, late, common enhancer classification having into consideration the scores assigned to each peak for each stage analyzed, we used deeptools multiBigwigSummary function to compute the average scores for each peak in our curated list at each stage. Subsequently, peaks with a coverage lower than 0.3 and peaks smaller that 600bp were excluded. Finally, we analyze the slope of H3K27ac coverage across the four stages and we classified enhancers as early (<-0.6), common (>-0.6, <0.6), or late (>0.6).

7 - C-HiC

The preprocessing and alignment of paired-end sequencing data, along with the filtering of mapped di-tags, were conducted using HiCUP pipeline (version 0.6.1) using default parameters for the configuration file and adding Nofill: 1 parameter. Bowtie2 (version 2.3.4.2) was used by the pipeline for mapping. Subsequently, filtered di-tags were processed with Juicer Tools (v1.9.9) to generate binned contact maps (5kb and 10kb) from valid and unique reads pairs with MAPQ≥30 and normalized maps using Knights and Ruiz matrix balancing. For binning and normalization, only the genomic region mm39:chr3:65103500-68603411 covering the Shox2 locus and adjacent TADs was considered. Subtraction maps were produced from the KR normalized maps and scaled together across their subdiagonals. CHiC maps of count values, as well as subtraction maps, were visualized as heatmaps in which values above the 99-th percentile were truncated for visualization purposes.

8-Virtual capture-C

Virtual capture-C profiles were generated from the hicup.bam files obtained during C-HiC analysis prior Juicer normalization. A 5 kb viewpoint for the Shox2 promoter was defined (mm39:chr3:66885043-66890041) to be used for contact analysis over the captured region (mm39:chr3:65103500-6860341). A contact pair was considered when one interaction fragment was in the viewpoint and its pair mate was outside of it. Reads were counted per restriction fragment, then binned to a regular 1-kb grid. If a fragment spanned more than one bin, the count value was distributed proportionally to the overlaps. Profiles were smoothed averaging over a sliding window of five bins. Finally, coverage normalization was performed by dividing the profiles by the sum of counts in the enriched captured region on chr3 and multiplied by 10^3.

9 - CUT & RUN

Paired FASTQ files of each sample were processed to subsample 78 M read pairs using seqtk toolkit with a random seed of 100. Reads from these subsampled FASTQ files were then processed first using CutAdapt version 4.9 to trim TruSeq adapter sequences using the option -a GATCGGAAGAGCACACGTCTGAACTCCAGTCAC and to trim low-quality ends using a threshold of 30 with option -q 30, and a minimum length of 15 bases with option -m 15. Then reads were mapped to our GRCm39/mm39_dsmCherry_P2A_CRE_EYFP customized mouse genome using Bowtie2 version 2.5.4 with the following options --very-sensitive --no-unal --no-mixed --no-discordant --dovetail -X 1000. Then reads with a mapping quality score (MAPQ) below 30 were removed, with SAMtools view version 1.21 (-q 30). Then, PCR duplicates were removed with MarkDuplicates tool from Picard version 2.27.4 before the BAM conversion to BED using bamtobed bedtools version 2.31.1. MACS2 version 2.2.9.1 was used to process the output BED file and obtain the coverage using the options --nomodel --keep-dup all --shift -100 --extsize 200 --call-summits -B 2. MACS2 output file was then normalized by million reads.

10 - Flow cytometry analysis
Flow cytometry analysis to apply a homogeneous gating to the different experiments and to extract cell proportion and performing a comparative analysis across the different datasets was performed with the FlowJo Software (version 10.9.0). Statistical t-test for pair-wise comparison of cell proportion changes was performed using R and ggplot2 (version 3.4.4).

Custome code availability
scRNA-seq, FACS analysis, RT-qPCRs, RNA-seq, ChIP-seq, C-HiC and CUT & RUN processing codes scripts are available on GitHub https://github.com/RROUCO/Scripts_for_Rouco_et_al_2025/ and citable via the following DOI https://doi.org/10.5281/zenodo.17143322here: https://github.com/RROUCO/Scripts_for_Rouco_et_al_2024 . GRCm39/mm39_eGFP-SV40pA custom genome and the filtered GTF file are available on Zenodo: https://zenodo.org/records/14865689.

For manuscripts utilizing custom algorithms or software that are central to the research but not yet described in published literature, software must be made available to editors and reviewers. We strongly encourage code deposition in a community repository (e.g. GitHub). See the Nature Portfolio guidelines for submitting code & software for further information.

Data

Policy information about availability of data

All manuscripts must include a data availability statement. This statement should provide the following information, where applicable:

- Accession codes, unique identifiers, or web links for publicly available datasets
- A description of any restrictions on data availability
- For clinical datasets or third party data, please ensure that the statement adheres to our policy

The Ssequencing data generated in this study are is available in the GEO repository under the accession number GSE262006: [https://www.ncbi.nlm.nih.gov/geo/query/acc.cgi?acc=GSE262006]. The entire forelimb Capture-C, RNA-seq and ChIP-seq at E10.5 and E13.5 datasets were obtained from GEO accession number GSE84795 [https://www.ncbi.nlm.nih.gov/geo/query/acc.cgi?acc=GSE84795]. The proximal and distal ATAC-seq E12.5 datasets were obtained from GEO accession number GSE194114 [https://www.ncbi.nlm.nih.gov/geo/query/acc.cgi?acc=GSE194114]. HOXD13 E12.5 Distal Forelimb ChIP-seq datasets were obtained from GEO accession number GSE81358 [https://www.ncbi.nlm.nih.gov/geo/query/acc.cgi?acc=GSE81358]. RNA-seq replicates 1 and 2 of entire forelimb E12.5 embryos with a G4 control background were taken from previous lab publications, accession number GSM8093970 [https://www.ncbi.nlm.nih.gov/geo/query/acc.cgi?acc=GSM8093970] and GSM8093971 [https://www.ncbi.nlm.nih.gov/geo/query/acc.cgi?acc= GSM8093971].

Research involving human participants, their data, or biological material

Policy information about studies with human participants or human data. See also policy information about sex, gender (identity/presentation), and sexual orientation and race, ethnicity and racism.

Reporting on sex and gender	N/A
Reporting on race, ethnicity, or other socially relevant groupings	N/A
Population characteristics	N/A
Recruitment	N/A
Ethics oversight	N/A

Note that full information on the approval of the study protocol must also be provided in the manuscript.

Field-specific reporting

Please select the one below that is the best fit for your research. If you are not sure, read the appropriate sections before making your selection.

☒ Life sciences ☐ Behavioural & social sciences ☐ Ecological, evolutionary & environmental sciences

For a reference copy of the document with all sections, see nature.com/documents/nr-reporting-summary-flat.pdf

Life sciences study design

All studies must disclose on these points even when the disclosure is negative.

Sample size

Sample sizes are standard for the used technologies techniques and each replicate represents an average measurement across several pooled samples.

- scRNA-seq: two replicates for each stage of interest E10.5, E11.5 and E12.5 were produced, except from E13.5 that only one replicate was produced. For each replicate micro-dissected limb pairs was prepared For E10.5, E11.5, E12.5 at least two pairs of limbs were pulled together, for E13.5 only one pair of limb was dissociated per tube. After the dissociation procedure totaling a 700cells/ul suspesion was prepared and 7000 cells were loaded on average on the Chromium Chip.

- For FACS-sorted experiments, the minimum number of cells required varied by experiment type: 3.5×10^4 - 1.5×10^5 cells for RNA-seq, 0.5×10^6 fixed frozen nuclei for ChIP-seq, and 1×10^6 fixed frozen nuclei for C-HiC, with the number of pooled limbs adjusted accordingly.

	For E10.5, E11.5, E12.5 at least two pairs of limbs were pulled together, for E13.5 only one pair of limb was dissociated per tube and for E14.5 embryos each limb was processed individually. For RNA-seq at least two replicates were processed, and for ChIP-seq and C-HiC one replicate was performed for sample of interest.<br><br>- Bulk limb RNA-seq utilized at least 2 biological replicates per sample type containing 0.5-1 x 10^6 cells. For E10.5, E11.5, E12.5 at least two pairs of limbs were pulled together, for E13.5 only one pair of limb was dissociated per tube and for E14.5 embryos each limb was processed individually.<br><br>-Bulk C-HiC at least two pairs of limbs were pulled together for dissociation then 1 x 10^6 cells were fixed. One replicate was done per genotype of interest.<br><br>-CUT & RUN Proximal and distal forelimbs from E13.5 embryos were micro-dissected , dissociated to obtain 250K cells. One replicate was done per genotype of interest.<br><br>-For RT-qPCR 5 biological replicates per sample type from proximal and distal forelimbs at E13.5 type were used.<br><br>- Flow cytometry experiments recorded at least 100 x10 ^3 cells per sample using the Beckman Coulter Cytoflex.
Data exclusions	No data was excluded from this study.
Replication	Experiments were replicated when their outcomes were critical for interpreting results. FACS-sorted and bulk RNA-seq experiments were conducted in duplicates, while ChIP-seq, C-HiC, and CUT&RUN were performed as singlicates. Flow cytometry experiments were replicated as needed for FACS-based studies, with signals pooled for graphical representation. Whole-mount in situ hybridization experiments were also performed in duplicates, with only one replicate imaged. RT-qPCRs were performed in triplicates following standard procedure. All replication attempts were successful.
Randomization	The samples were not randomized. In this study, it was crucial to identify the genotypes and types of cells (dmCherry+/-; EYFP+/-) analyzed since they required pooling before being processed for experiments.
Blinding	Investigators were not blinded as the micro-dissection of mouse embryos and subsequent analyses necessitated awareness of the genotypes and types of cells (dmCherry+/-; EYFP+/-) involved.

Reporting for specific materials, systems and methods

We require information from authors about some types of materials, experimental systems and methods used in many studies. Here, indicate whether each material, system or method listed is relevant to your study. If you are not sure if a list item applies to your research, read the appropriate section before selecting a response.

Materials & experimental systems

n/a	Involved in the study
☐	☒ Antibodies
☐	☒ Eukaryotic cell lines
☒	☐ Palaeontology and archaeology
☐	☒ Animals and other organisms
☒	☐ Clinical data
☒	☐ Dual use research of concern
☒	☐ Plants

Methods

n/a	Involved in the study
☐	☒ ChIP-seq
☐	☒ Flow cytometry
☒	☐ MRI-based neuroimaging

Antibodies

Antibodies used	Polyclonal rabbit anti-H3K27ac antibody from Diagenode, Cat-No. c15410174 RRID:AB_2716835, was used at a dilution of 1/400; Rabbit Anti-Histone H3, trimethyl (Lys27) Polyclonal antibody, (Milipore Cat# 07-449, RRID:AB_310624) was used at a dilution of 1/330; Polyclonal rabbit Anti-HOXD13 antibody (Abcam AB229234)
Validation	The antibody anti-H3K27ac and Anti-Histone H3, trimethyl (Lys27) were validated for ChIP or Immunoprecipitation usage on the manufacturer's website. Additionally, we have extensively used this same antibody in a previous study and achieved high reproducibility. The Anti-HOXD13 antibody used for CUT&RUN has been validated for western blot and proposed by Abcam as a replacement for the previous antibody Ab19866, which was successfully used for CUT&RUN.

Eukaryotic cell lines

Policy information about cell lines and Sex and Gender in Research

Cell line source(s)	Male G4 mouse ESCs (Jorge et al., 2007)
Authentication	Genetically modified pluripotent mouse ESCs were authenticated through the production of fetuses via tetraploid aggregations (Artus and Hadjantonakis, 2011), and subsequent genotyping confirmed the presence of the desired mutations

	in the cells.
Mycoplasma contamination	All cell lines tested negative for mycoplasma contamination.
Commonly misidentified lines (See ICLAC register)	No commonly misidentied cell lines were used.

Animals and other research organisms

Policy information about studies involving animals; ARRIVE guidelines recommended for reporting animal research, and Sex and Gender in Research

Laboratory animals	Mouse (Mus musculus) embryos were obtained following the tetraploid complementation procedure of the genetically-engineered male G4 (129/sv x C57BL/6 F1 hybrid) mESCs (Artus and Hadjantonakis, 2011; George et al., 2007). In brief, two days before the aggregation procedure, desired clones were thawed, seeded on male and female CD1 feeders, and grown. Donor tetraploid embryos were provided from in vitro fertilization using c57bl6J x B6D2F1 backgrounds. Aggregated embryos were transferred into CD1 or B6CBA foster females. Animals were obtained from Janvier laboratories or from in house crosses. Embryos were collected in 1X DPBS (Gibco, 14190-094) at the desired stage depending on the downstream protocol.
Wild animals	There was no wild animals in this study.
Reporting on sex	Sex was not a factor in the study design. However, for the aggregations, embryos were generated using male G4 mouse ESCs (Jorge et al., 2007), resulting in male embryos
Field-collected samples	There was no field collected animals in this study.
Ethics oversight	Animal work performed in Geneva complied with all relevant ethical regulations of the University of Geneva and followed procedures approved by the animal care and experimentation authorities of the Canton of Geneva, Switzerland (animal protocol numbers GE/89/19 and GE192A).

Note that full information on the approval of the study protocol must also be provided in the manuscript.

Plants

Seed stocks	N/A
Novel plant genotypes	N/A
Authentication	N/A

ChIP-seq

Data deposition

- [x] Confirm that both raw and final processed data have been deposited in a public database such as GEO.
- [x] Confirm that you have deposited or provided access to graph files (e.g. BED files) for the called peaks.

Data access links *May remain private before publication.*	ChIP-seq dataset are available in the GEO repository under the accession number GSE262006 under the subseries GSE262005: https://www.ncbi.nlm.nih.gov/geo/query/acc.cgi?acc=GSE262005
Files in database submission	H3K27ac_FL_Shox2trac_E105_MAINTAIN_rep1_macs_SR200_norm.bw<br>H3K27ac_FL_Shox2trac_E115_MAINTAIN_rep1_macs_SR200_norm.bw<br>H3K27ac_FL_Shox2trac_E125_MAINTAIN_rep1_macs_SR200_norm.bw<br>H3K27ac_FL_Shox2trac_E135_MAINTAIN_rep1_macs_SR200_norm.bw<br>H3K27ac_HL_Shox2trac_E115_MAINTAIN_rep1_macs_SR200_norm.bw<br>H3K27ac_HL_Shox2trac_E125_MAINTAIN_rep3_macs_SR200_norm.bw<br>H3K27ac_HL_Shox2trac_E135_MAINTAIN_rep1_macs_SR200_norm.bw<br>H3K27ac_FL_Shox2trac_E115_INACTIVE_rep1_macs_SR200_norm.bw<br>H3K27ac_FL_Shox2trac_E115_DECOM_rep1_macs_SR200_norm.bw<br>H3K27ac_FL_Shox2trac_E125_DECOM_rep3_macs_SR200_norm.bw<br>H3K27ac_FL_Shox2trac_E135_DECOM_rep3_macs_SR200_norm.bw<br>H3K27ac_HL_Shox2trac_E115_INACTIVE_rep1_macs_SR200_norm.bw<br>H3K27ac_HL_Shox2trac_E115_DECOM_rep2_macs_SR200_norm.bw<br>H3K27ac_HL_Shox2trac_E125_DECOM_rep3_macs_SR200_norm.bw<br>H3K27ac_HL_Shox2trac_E135_DECOM_rep1_macs_SR200_norm.bw

H3K27ac_FL_Shox2trac_E125_INACTIVE_rep1_macs_SR200_norm.bw
H3K27ac_FL_Shox2trac_E135_INACTIVE_rep1_macs_SR200_norm.bw
H3K27ac_HL_Shox2trac_E125_INACTIVE_rep3_macs_SR200_norm.bw
H3K27ac_HL_Shox2trac_E135_INACTIVE_rep1_macs_SR200_norm.bw
H3K27ac_FL_Shox2trac_E105_MAINTAIN_rep1_macs_SR200_peaks.broadPeak
H3K27ac_FL_Shox2trac_E115_MAINTAIN_rep1_macs_SR200_peaks.broadPeak
H3K27ac_FL_Shox2trac_E125_MAINTAIN_rep1_macs_SR200_peaks.broadPeak
H3K27ac_FL_Shox2trac_E135_MAINTAIN_rep1_macs_SR200_peaks.broadPeak
H3K27ac_HL_Shox2trac_E115_MAINTAIN_rep1_macs_SR200_peaks.broadPeak
H3K27ac_HL_Shox2trac_E125_MAINTAIN_rep3_macs_SR200_peaks.broadPeak
H3K27ac_HL_Shox2trac_E135_MAINTAIN_rep1_macs_SR200_peaks.broadPeak
H3K27ac_FL_Shox2trac_E115_INACTIVE_rep1_macs_SR200_peaks.broadPeak
H3K27ac_FL_Shox2trac_E115_DECOM_rep1_macs_SR200_peaks.broadPeak
H3K27ac_FL_Shox2trac_E125_DECOM_rep3_macs_SR200_peaks.broadPeak
H3K27ac_FL_Shox2trac_E135_DECOM_rep3_macs_SR200_peaks.broadPeak
H3K27ac_HL_Shox2trac_E115_INACTIVE_rep1_macs_SR200_peaks.broadPeak
H3K27ac_HL_Shox2trac_E115_DECOM_rep2_macs_SR200_peaks.broadPeak
H3K27ac_HL_Shox2trac_E125_DECOM_rep3_macs_SR200_peaks.broadPeak
H3K27ac_HL_Shox2trac_E135_DECOM_rep1_macs_SR200_peaks.broadPeak
H3K27ac_FL_Shox2trac_E125_INACTIVE_rep1_macs_SR200_peaks.broadPeak
H3K27ac_FL_Shox2trac_E135_INACTIVE_rep1_macs_SR200_peaks.broadPeak
H3K27ac_HL_Shox2trac_E125_INACTIVE_rep3_macs_SR200_peaks.broadPeak
H3K27ac_HL_Shox2trac_E135_INACTIVE_rep1_macs_SR200_peaks.broadPeak
H3K27ac_FL_Shox2trac_E105_MAINTAIN_rep1_S29_L008_R1_001.fastq.gz
H3K27ac_FL_Shox2trac_E115_MAINTAIN_rep1_S30_L008_R1_001.fastq.gz
H3K27ac_FL_Shox2trac_E125_MAINTAIN_rep1_S31_L008_R1_001.fastq.gz
H3K27ac_FL_Shox2trac_E135_MAINTAIN_rep1_S32_L008_R1_001.fastq.gz
H3K27ac_HL_Shox2trac_E115_MAINTAIN_rep1_S4_L001_R1_001.fastq.gz
H3K27ac_HL_Shox2trac_E125_MAINTAIN_rep3_S15_L001_R1_001.fastq.gz
H3K27ac_HL_Shox2trac_E135_MAINTAIN_rep1_S2_L007_R1_001.fastq.gz
H3K27ac_FL_Shox2trac_E115_INACTIVE_rep1_S3_L004_R1_001.fastq.gz
H3K27ac_FL_Shox2trac_E115_DECOM_rep1_S33_L008_R1_001.fastq.gz
H3K27ac_FL_Shox2trac_E125_DECOM_rep3_S16_L001_R1_001.fastq.gz
H3K27ac_FL_Shox2trac_E135_DECOM_rep3_S17_L001_R1_001.fastq.gz
H3K27ac_HL_Shox2trac_E115_INACTIVE_rep1_S20_L001_R1_001.fastq.gz
H3K27ac_HL_Shox2trac_E115_DECOM_rep2_S18_L001_R1_001.fastq.gz
H3K27ac_HL_Shox2trac_E125_DECOM_rep3_S19_L001_R1_001.fastq.gz
H3K27ac_HL_Shox2trac_E135_DECOM_rep1_S5_L001_R1_001.fastq.gz
H3K27ac_FL_Shox2trac_E125_INACTIVE_rep1_S4_L004_R1_001.fastq.gz
H3K27ac_FL_Shox2trac_E135_INACTIVE_rep1_S5_L004_R1_001.fastq.gz
H3K27ac_HL_Shox2trac_E125_INACTIVE_rep3_S21_L001_R1_001.fastq.gz
H3K27ac_HL_Shox2trac_E135_INACTIVE_rep1_S22_L001_R1_001.fastq.gz
H3K27me3_HL_E135_Shox2trac_DECOM_Rep1_3ul_macs_SR200_norm.bw
H3K27me3_HL_E135_Shox2trac_DECOM_Rep1_3ul_macs_SR200_peaks.broadPeak
H3K27me3_HL_E135_Shox2trac_DECOM_Rep1_S4_L007_R1_001.fastq.gz
H3K27me3_HL_E135_Shox2trac_INACTIVE_Rep1_3ul_macs_SR200_norm.bw
H3K27me3_HL_E135_Shox2trac_INACTIVE_Rep1_3ul_macs_SR200_peaks.broadPeak
H3K27me3_HL_E135_Shox2trac_INACTIVE_Rep1_S7_L001_R1_001.fastq.gz
H3K27me3_HL_E135_Shox2trac_MAINTAIN_Rep1_3ul_macs_SR200_norm.bw
H3K27me3_HL_E135_Shox2trac_MAINTAIN_Rep1_3ul_macs_SR200_peaks.broadPeak
H3K27me3_HL_E135_Shox2trac_MAINTAIN_Rep1_S3_L007_R1_001.fastq.gz

Genome browser session
(e.g. UCSC)

N/A

Methodology

Replicates

ChIP-seq was performed in singlicates using FACS-sorted cells from pooled limbs.

Sequencing depth

H3K27ac_FL_Shox2trac_E105_MAINTAIN_rep1_S29_L008_R1_001.fastq.gz: 20936620 reads
H3K27ac_FL_Shox2trac_E115_MAINTAIN_rep1_S30_L008_R1_001.fastq.gz: 44815829 reads
H3K27ac_FL_Shox2trac_E125_MAINTAIN_rep1_S31_L008_R1_001.fastq.gz: 53922448 reads
H3K27ac_FL_Shox2trac_E135_MAINTAIN_rep1_S32_L008_R1_001.fastq.gz: 46595434 reads
H3K27ac_HL_Shox2trac_E115_MAINTAIN_rep1_S4_L001_R1_001.fastq.gz: 47541265 reads
H3K27ac_HL_Shox2trac_E125_MAINTAIN_rep3_S15_L001_R1_001.fastq.gz: 101334643 reads
H3K27ac_HL_Shox2trac_E135_MAINTAIN_rep1_S2_L007_R1_001.fastq.gz: 49899632 reads
H3K27ac_FL_Shox2trac_E115_INACTIVE_rep1_S3_L004_R1_001.fastq.gz: 55644434 reads
H3K27ac_FL_Shox2trac_E115_DECOM_rep1_S33_L008_R1_001.fastq.gz: 36640537 reads
H3K27ac_FL_Shox2trac_E125_DECOM_rep3_S16_L001_R1_001.fastq.gz: 91777208 reads
H3K27ac_FL_Shox2trac_E135_DECOM_rep3_S17_L001_R1_001.fastq.gz: 99465206 reads

H3K27ac_HL_Shox2trac_E115_INACTIVE_rep1_S20_L001_R1_001.fastq.gz: 67279378 reads
H3K27ac_HL_Shox2trac_E115_DECOM_rep2_S18_L001_R1_001.fastq.gz: 77701394 reads
H3K27ac_HL_Shox2trac_E125_DECOM_rep3_S19_L001_R1_001.fastq.gz: 100264452 reads
H3K27ac_HL_Shox2trac_E135_DECOM_rep1_S5_L001_R1_001.fastq.gz: 47656599 reads
H3K27ac_FL_Shox2trac_E125_INACTIVE_rep1_S4_L004_R1_001.fastq.gz: 75922628 reads
H3K27ac_FL_Shox2trac_E135_INACTIVE_rep1_S5_L004_R1_001.fastq.gz: 61893288 reads
H3K27ac_HL_Shox2trac_E125_INACTIVE_rep3_S21_L001_R1_001.fastq.gz: 104135077 reads
H3K27ac_HL_Shox2trac_E135_INACTIVE_rep1_S22_L001_R1_001.fastq.gz: 81314794 reads
H3K27me3_HL_E135_Shox2trac_INACTIVE_Rep1_S7_L001_R1_001.fastq.gz: 45102437 reads
H3K27me3_HL_E135_Shox2trac_MAINTAIN_Rep1_S3_L007_R1_001.fastq.gz: 55577564 reads
H3K27me3_HL_E135_Shox2trac_DECOM_Rep1_S4_L007_R1_001.fastq.gz: 54699934 reads

Antibodies

H3K27Ac ChIP-seq was performed using C15410174 (Diagenode) with 1/400 dilution of the antibody. H3K27me3 ChIP-seq was performed using Milipore Cat# 07-449 with 1/330 dilution of the antibody.

Peak calling parameters

For coverage and peak analysis from FACS sorted datasets generated for this study, reads were extended by 200 base pairs and processed using MACS2 version 2.2.7.1 with the parameters --broad --nolambda --broad-cutoff 0.05 --nomodel --gsize mm --extsize 200 -B 2 for broad peak calling, in the case of H3K27ac ChIP produced from the FACS sorted cells for this study. For reanalyzed datasets (Andrey et al., 2017; Sheth et al., 2016) coverage and peak analysis, reads were extended by 200 base pairs and processed using MACS2 version 2.2.7.1.with the parameters --call-summits --nomodel --extsize 200 -B 2 for narrow peak calling. The coverage normalization was performed by MACS2, adjusting for the total millions of tags used in the analysis.

Data quality

Data quality was manually confirmed through the observation of strong H3K27ac and H3K27me3 signal enrichment at known limb developmental loci, such as the Hox clusters and housekeeping genes like Actb or Foxa1.

Software

Reads from ChIP-seq sequencing either from datasets generated for this study or from GEO datasets that we wanted to reanalyze (Andrey et al., 2017; Sheth et al., 2016) were processed first using CutAdapt version 1.18 (Martin, 2011) to trim TruSeq or Nextseq adapter sequences and low-quality bases , specifying the adapter sequence with -a GATCGGAAGAGCACACGTCTGAACTCCAGTCAC (for TruSeq) or -a CTGTCTCTTATACACATCTCCGAGCCCACGAGAC (for Nextseq), a quality threshold of 30 with -q30, and a minimum length of 15 bases with -m15. Then reads were mapped to our GRCm39/mm39_dsmCherry_P2A_CRE_EYFP customized mouse genome, in the case of the datasets generated for this study, or to the GRCm39/mm39 in the case of the reanalyzed datasets, using Bowtie2 version 2.3.5.1 (Langmead and Salzberg, 2012) with its default settings. Subsequently, only reads with a mapping quality score (MAPQ) of 30 or higher were retained, as filtered with SAMtools view version 1.10 (Danecek et al., 2021). For coverage and peak analysis, reads were extended by 200 base pairs and processed using MACS2 version 2.2.7.1 (Zhang et al., 2008) with the parameters --broad --nolambda --broad-cutoff 0.05 --nomodel --gsize mm --extsize 200 -B 2 for broad peak calling, in the case of H3K27ac ChIP produced from the FACS sorted cells for this study. For reanalyzed datasets coverage and peak analysis, reads were extended by 200 base pairs and processed using MACS2 version 2.2.7.1 (Zhang et al., 2008) with the parameters --call-summits --nomodel --extsize 200 -B 2 for narrow peak calling. The coverage normalization was performed by MACS2, adjusting for the total millions of tags used in the analysis.

Early, common, late putative enhancers classification on entire forelimb datasets: RNA-seq FASTQ files from two replicates of entire forelimbs at E10.5 and E13.5 (Andrey et al., 2017) were re-analyzed following the RNA-seq pipeline previously described (see RNA-seq analysis in this Material and Methods section). Genes related to limb development were selected for downstream analysis. Average of normalized FPKM values was calculated and used to compute the ratio among E10.5 and E13.5 datasets. Then, since we were interested in genes having stable expression between E10.5 and E13.5, they were filtered to keep those with FPKM values bigger than 5, at both stages, and we excluded genes having a fold change larger than 3 between the two stages. By applying this filtering 90 genes were selected (Supplementary Table 1). To analyze putative enhancers ChIP-seq H3K27Ac datasets of entire forelimbs at E10.5 and E13.5 (Andrey et al., 2017) were first reanalyzed following the ChIP-seq pipeline previously described in this material and methods section. H3K27ac MACS2 narrowpeaks were then restricted within the interaction domain defined by promoter Capture-C (Andrey et al., 2017) of the 90 filtered genes, using bedtools (version v2.30.0) intersect function (Quinlan and Hall, 2010). Then, H3K27Ac peaks around gene promoters were excluded by filtering against a -2kb/500bp window centered at the transcription start site of coding genes using again bedtools intersect. Remaining peaks were extended by +/- 300bp, using bedtools slope and merge function (Quinlan and Hall, 2010). H3K27Ac peaks were then classified as putative common enhancers when present in both E10.5 and E13.5 using bedtools intersect. H3K27Ac peaks present only in the E10.5 dataset were classified as putative early enhancers while H3K27Ac peaks present only in the E13.5 dataset were classified as putative late enhancers. Putative enhancers were then assigned to gene interaction domain (Supplementary Table 1). In those cases, where putative enhancers were within the overlapping region of two domains putative enhancers were assigned to the two loci. H3K27Ac peaks classified in the three categories were then processed to calculate scores using the compareMatrix function from bedtools. This matrix was then used to produce a heatmap (tornado plot) of the H3K27Ac peaks per category using plotHeatmap function from bedtools.

Early, common, late putative enhancers classification on FACS sorted maintaining forelimb datasets
MACS2 BroadPeak files from FACS sorted maintaining cells from forelimbs at E10.5, E11.5, E12.5 and E13.5 were used to build bed files. These files were used to merge peaks within 600bp of each other using bedtools (version v2.30.0) (Quinlan and Hall, 2010). Then, bedops (version 2.4.41) (Neph et al., 2012) --merge operation was used to flatten all disjoint, overlapping, and adjoining element regions into contiguous, disjoint regions peaks among the four different stages. Subsequently, peaks were extended by +/- 300bp, using bedtools slope function. Since, we wanted only to explore the putative enhancers of Shox2 locus using bedtools intersect we selected only the region with the following coordinates mm39 chr3:65,885,132-67,539,263, in that way we created a list of peaks of interest. Peaks falling on gene promoters were manually excluded. Then as we wanted to stablish our new early, late, common enhancer classification having into consideration the scores assigned to each peak for each stage analyzed, we used deeptools (Ramirez et al., 2016) multiBigwigSummary function to compute the average scores for each peak in our curated list at each stage. Subsequently, peaks with a coverage lower than 0.3 and peaks smaller that 600bp were excluded. Finally, we analyze the slope of H3K27ac coverage across the four stages and we classified enhancers as early (<-0.6), common (>-0.6, <0.6), or late (>0.6).

Flow Cytometry

Plots

Confirm that:

- [x] The axis labels state the marker and fluorochrome used (e.g. CD4-FITC).
- [x] The axis scales are clearly visible. Include numbers along axes only for bottom left plot of group (a 'group' is an analysis of identical markers).
- [x] All plots are contour plots with outliers or pseudocolor plots.
- [x] A numerical value for number of cells or percentage (with statistics) is provided.

Methodology

Sample preparation	Forelimb or Hindlimb buds of E10.5, E11.5, E12.5, E13.5 or E14.5 control (Shox2dmCherry/+;RosaEYFP/+) or mutant embryos were micro-dissected in 1X DPBS (Gibco, 14190-094) and placed in 1.5ml tubes. For E10.5, E11.5, E12.5 at least two pairs of limbs were pulled together, for E13.5 only one pair of limb was dissociated per tube and for E14.5 embryos each limb was processed individually. After DPBS removal, each tube containing pairs of limb buds were incubated with 400µl trypsin-EDTA 0.25% (Thermo Fischer Scientific, 25300062) supplemented with 40µl of 5% BSA in PBS (Sigma Aldrich, A7906-100G), during 8-9 min for small embryos (E10.5 and E11.5) or 12-15 min for larger embryos (E12.5, E13.5 or E14.5) at 37°C in a Thermomixer with a resuspension step after the first 6 min and at the end of the rest of the incubation time. After Trypsin inactivation with one volume of 5% BSA, cells were passed through a 40µm cell strainer and another volume of 5% BSA was added to wash the cell strainer. Cells were spun at 400g for 5min at 4°C and resuspended in 1%BSA in PBS (5mM Na-Butyrate was added in case the cells were processed to be sorted and later used for downstream ChIP experiments). The single-cell suspension obtained from this process were later used for subsequent flow cytometry experiments.
Instrument	BD FACS Aria and Beckman Coulter Cytoflex analyzer
Software	Flow cytometry analysis from Cytoflex recorded data was performed with the FlowJo Software (version 10.9.0) to apply a homogeneous gating to the different experiments, and to extract cell proportions and perform a comparative analysis across the different datasets. Statistical two-sided t-test for pair-wise comparison of cell proportion changes was performed using R and ggplot2 (version 3.4.4).
Cell population abundance	The borders to determine population abundance are displayed in main and supplementary figures
Gating strategy	The gating stragegy is displayed in Supplementary Figure 4 (BD Aria) and 7 (Cytoflex).<br>Fluorescent-activated cell sorting (FACS) was used to isolate cell populations based on the dmCherry and EYFP fluorescent signal by using the BD FACS Aria fusion with a blue laser (488nm, filter 530/30) for the EYFP signal and with a YG laser (561nm, filter 610/20) for the dmCherry signal. When sorted of cells was not required and only recording of cell population proportion was needed, we used the Beckman Coulter Cytoflex analyzer with a blue laser (488nm, filter 525/40) for the EYFP signal and with a YG laser (561nm, filter 620/20) for the dmCherry signal. In both cases, a first FSC/SCC gating was set between 30/40 and 210/240 to exclude debris followed by dead cells removal using a viability dye (DAPI, AppliChem, #A10010010 for the BDAria or DRAQ7, Invitrogen, D15106 for the Cytoflex).

- [x] Tick this box to confirm that a figure exemplifying the gating strategy is provided in the Supplementary Information.
